# A molecular framework for grain number determination in barley

**DOI:** 10.1126/sciadv.add0324

**Published:** 2023-03-03

**Authors:** Yongyu Huang, Roop Kamal, Nandhakumar Shanmugaraj, Twan Rutten, Venkatasubbu Thirulogachandar, Shuangshuang Zhao, Iris Hoffie, Goetz Hensel, Jeyaraman Rajaraman, Yudelsy Antonia Tandron Moya, Mohammad-Reza Hajirezaei, Axel Himmelbach, Naser Poursarebani, Udda Lundqvist, Jochen Kumlehn, Nils Stein, Nicolaus von Wirén, Martin Mascher, Michael Melzer, Thorsten Schnurbusch

**Affiliations:** ^1^ Leibniz Institute of Plant Genetics and Crop Plant Research (IPK), Corrensstr. 3, OT Gatersleben, 06466 Seeland, Germany.; ^2^ Nordic Genetic Resource Center, Alnarp SE-23053, Sweden.; ^3^ Center for Integrated Breeding Research (CiBreed), Georg-August-University, Göttingen, Germany.; ^4^ German Centre for Integrative Biodiversity Research (iDiv) Halle-Jena-Leipzig, Leipzig, Germany.; ^5^ Martin Luther University Halle-Wittenberg, Faculty of Natural Sciences III, Institute of Agricultural and Nutritional Sciences, 06120 Halle, Germany.

## Abstract

Flowering plants with indeterminate inflorescences often produce more floral structures than they require. We found that floral primordia initiations in barley (*Hordeum vulgare* L.) are molecularly decoupled from their maturation into grains. While initiation is dominated by flowering-time genes, floral growth is specified by light signaling, chloroplast, and vascular developmental programs orchestrated by barley *CCT MOTIF FAMILY 4* (*HvCMF4*), which is expressed in the inflorescence vasculature. Consequently, mutations in *HvCMF4* increase primordia death and pollination failure, mainly through reducing rachis greening and limiting plastidial energy supply to developing heterotrophic floral tissues. We propose that *HvCMF4* is a sensory factor for light that acts in connection with the vascular-localized circadian clock to coordinate floral initiation and survival. Notably, stacking beneficial alleles for both primordia number and survival provides positive implications on grain production. Our findings provide insights into the molecular underpinnings of grain number determination in cereal crops.

## Teaser

Signaling from inflorescence vasculature via CCT MOTIF FAMILY4 determine distal floral fate and yield traits in barley.

## INTRODUCTION

**Fig. 1.**
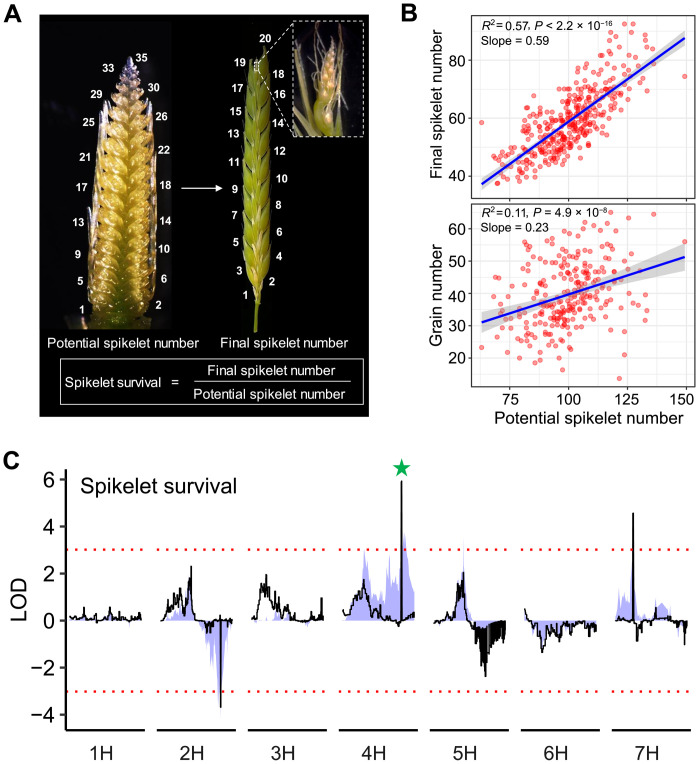
Natural variation of barley potential spikelet number and its survival. (**A**) Representative spike images depicting spikelet survival phenotypes in barley. Insert highlights dead spikelet. Numbers indicate rachis nodes, which equal to spikelet number when tripled. (**B**) Relationships of potential spikelet number with final spikelet number and grain number. (**C**) QTL mapping for spikelet survival. Colored shades and black lines indicate logarithm of the odds (LOD) scores based on single-marker analysis and composite interval mapping methods, respectively. Green star highlights the *HvCMF4*-related locus (see Fig. 2). Red dashed horizontal lines depict the suggestive thresholds based on 1000 permutations. LOD values above or below zero represent alleles with positive additive effects from BCC719 or BCC149, respectively.

Modifying inflorescences with higher grain capacity is vital for crop grain production. One recurring target is to select inflorescences with more branches or floral structures (*1*). Prominent examples include genes affecting floral identity or meristem determinacy, for which natural or induced variants profoundly change floral primordium number (*2*–*4*). However, for temperate cereal crops, such as wheat (*Triticum* spp.) and barley, excessive floral structures can result in a degeneration penalty due to the indeterminate nature of meristems (i.e., spikelet meristem in wheat and inflorescence meristem in barley) (Fig. 1A) (*5*, *6*). On the other hand, the manifestation of this reproductive potential can be accentuated by environmental fluctuations such as light, temperature, and nutrition (*7*, *8*). Increasing the fraction of surviving florets/spikelets may thus improve grain yield in cereals.

## RESULTS

### Grain number determination in barley is specified by both spikelet initiation and survival

We embarked on our study with the initial assumption that the final spikelet number is exclusively determined by the potential spikelet number by phenotyping 358 six-rowed spring barleys representing global diversity (table S1) (*9*). Notably, the potential spikelet number only accounted for ~57% of the observed variation in final spikelet number (*R*^2^ = 0.57) and ~11% for grain number (Fig. 1B), while the potential spikelet number itself was positively correlated with days to heading (fig. S1A, left). We therefore examined the potential spikelet number and dates to heading in 10 early maturity mutants (table S2) and one biparental mapping population segregating for days to heading (table S3). We found that early maturity mutants produced a lower potential spikelet number than wild-type Bowman (hereafter, BW) (fig. S1A, right). In contrast, plants in the segregating population with highly delayed heading conferred by epistatic interaction between VERNALIZATION 1 (*VRN-H1*) and *VRN-H2* (*10*) not only had a higher potential spikelet number but also aborted more (fig. S1, B to E). Together, these data suggest that flowering-time genes dominate the initiation phase to control the potential spikelet number, but the final spikelet number is conditioned on a subsequent growth phase that defines spikelet survival, a phenomenon whose genetic regulation in barley has not been explored.

**Fig. 2.**
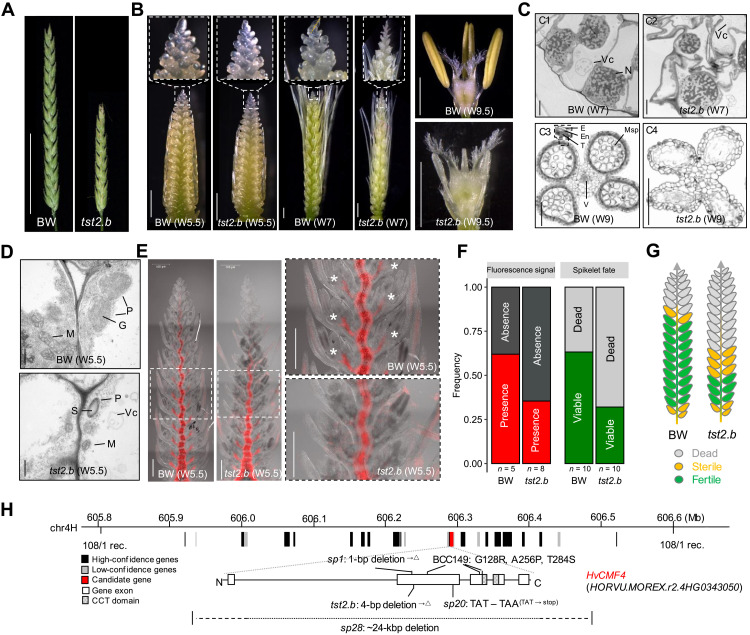
Inflorescence architectures of BW and *tst2.b* and gene isolation. (**A** and **B**) Inflorescences of BW and *tst2.b.* (A) Grain filling stage; (B) before anthesis stage. Carpels and anther from apical spikelets at W9.5 are shown on the right. (**C**) Cellular structures of inflorescence meristems from BW and *tst2.b* at W7 (C1 and C2) and anther from spikelet #14 in BW relative to spikelet #23 in *tst2.b* at W9 (C3 and C4; see also fig. S5). Vc, vesicle; N, nucleus; E, epidermis; En, endothecium; T, tapetum; Msp, microspore; V, vasculature. (**D**) TEM observation from transverse sections of BW rachis 13 relative to *tst2.b* rachis 23 at W5.5 stage (see also fig. S5). M, mitochondria; P, plastid; S, starch; G, grana. (**E**) Chlorophyll in situ distribution pattern in BW and *tst2.b* spikes at W5.5 stage revealed by autofluorescence. Asterisks highlight the presence of greening extension from rachis to spikelet, which is absent in *tst2.b* spike counterparts. (**F**) A percent stacked bar chart highlights the presence/absence of greening extension as a proxy of spikelet survival. (**G**) A schematic depiction of spikelet degeneration and death in BW and *tst2.b*. (**H**) Gene fine mapping, mutant study, and Cas9-mediated knockout. Rec., recombinant. “△” denotes frameshift mutation followed by a stop codon. Scale bars, 5 cm (A), 1 mm (B), 100 μm (C), 250 nm (D), and 500 μm (E).

Using the biparental population, we first mapped three quantitative trait loci (QTL) for spikelet survival on chromosomes 2H, 4H, and 7H (Fig. 1C and table S4), among which the 4H QTL interval was found to overlap with an introgression region in the classical backcrossed (BC_6_) mutant *tip sterile 2.b* (*tst2.b*; known as BW883; see Materials and Methods). In addition, we searched for mutants with a lowered spikelet number due to reduced spikelet survival and identified four in different genetic backgrounds (table S2), including three *short spike* (*sp*) mutants (*sp1*, *sp20*, and *sp28*). Compared with their wild-type backgrounds, all mutants produced shorter spikes with a varying degree of sterility in apical spikelets (see below). We continued to use *tst2.b* for our further studies as it was able to set grains under greenhouse conditions, whereas the three *sp* mutants appeared to be completely sterile (grain set only in the field). Dissection of developing apices revealed that spikelet initiation until Waddington stage 4.5 (W4.5) (*11*) remained the same between *tst2.b* and BW (fig. S2). Measurement of meristem size revealed that *tst2.b* spikes became developmentally arrested at W4.5 compared with BW (fig. S2, B and C). Later, at W5.5 stage, the inflorescence meristem dome collapsed in *tst2.b*, and subsequently, spikelets degenerated and died in a basipetal manner (Fig. 2, B to D, and fig. S2D). In addition, spikelet sterility after anthesis was higher in *tst2.b* because of anther developmental defects (Fig. 2, B and C, and fig. S3, A to D). Spikelet initiation in *sp1*, *sp20*, and *sp28* also appeared to be normal compared with their respective wild types, as potential spikelet number remained largely unchanged; however, final spikelet number, grain number, and spike length were drastically reduced (fig. S4, A and B). Because of their strong male sterility, we were only able to confirm allelism for *sp20* with *tst2.b*, for which we found alleles from either mutants that failed to complement each other in their F_1_ hybrids (fig. S4C). Thus, our phenotypic analysis revealed two distinct developmental problems in the mutant spikes: increased pre-anthesis tip degeneration and later male sterility (Fig. 2G).

The barley spike features an early appearance of greening during development that declines acropetally along the rachis. However, the functional relevance of greening to spikelet development is unknown. Under microscopic dissections, we observed a pale-green phenotype in *tst2.b* spikes at W5.5 and onward (Fig. 2B). Further chlorophyll autofluorescence imaging revealed that the acropetal declining pattern of greening was more pronounced in *tst2.b* spikes compared with BW at W5.5 stage. We counted individual spikelets with greening extension from the main rachis in both BW and *tst2.b* and found that greening is highly predictable for spikelet survival (Fig. 2, E and F; Pearson’s chi-square test, *P* > 0.05). We confirmed this by directly measuring chlorophyll content along the spike (fig. S4D). Transmission electron microscopy (TEM) analyses revealed that plastid differentiation was impaired in *tst2.b* rachises at W5.5 and onward. For example, plastid sizes were smaller in *tst2.b* rachis cells and tended to contain more starch, as is typically seen in amyloplasts (Fig. 2D and fig. S5, A to D). Similar plastidial defects were observed at around stages 5 and 6 of anther development (*12*) in cells of the endothecium and tapetum (fig. S5, E to H), which are two critical tissues for anther functioning. TEM observation also revealed signs of stress response in *tst2.b* rachis and anther cells, such as the appearances of vesicles and lipid droplets (*13*). We applied the photosynthesis inhibitors norflurazon and lincomycin to BW to ascertain whether imbalanced chloroplast development is the immediate cause for spikelet abortion (see Materials and Methods). Crucially, both chemicals suppressed spike growth in a dose-dependent manner (fig. S4, E and F). Together, these data indicate that *TST2* plays a pivotal role in ensuring proper plastid development and spike greening after the W4.5 stage to sustain spikelet growth; it also immediately highlights the involvement of chloroplast developmental gradients in the quantitative variation of spikelet survival, especially when considering the quantitative nature of chlorophyll accumulation among genotypes.

### *TST2* is a CCT motif family gene expressed in the spike vasculature

Whole-genome sequencing revealed seven chromosomal introgressions from the originally mutagenized cultivar (Ackermann’s Donaria) that retained the *tst2.b* genome, including an introgression on 4H coinciding with the spikelet survival QTL (fig. S6A). Further map-based cloning revealed a frameshift mutation [4–base pair (bp) deletion] specific to *tst2.b* in the second exon of *HORVU.MOREX.r2.4HG0343050* on 4H, which was independently mutated in *sp1*, *sp20*, and *sp28* (Fig. 2H, fig. S6B, table S6, and Materials and Methods). The causality was further validated by Cas9-triggered site-directed mutagenesis (fig. S6, D to F). Moreover, we found that BCC149, one of the two parental lines of the QTL mapping population, differed from BCC719 and BW by three amino acid substitutions. Two of which (A256P and T284S) altered highly conserved sites close to the CCT [CONSTANS (CO), CO-like, and TIMING OF CAB1 (TOC1)] domain (fig. S6C), suggesting that natural missense mutations in *HORVU.MOREX.r2.4HG0343050* may underpin the 4H QTL for spikelet survival. *HORVU.MOREX.r2.4HG0343050* encodes HvCMF4, a barley member of the CCT Motif Family proteins (*14*) that only contains a single CCT domain at its C terminus without additional domains commonly seen in other CCT genes, such as PSEUDO-RESPONSE REGULATOR (PRRs) and B-box domain (CONSTANS and CONSTANS-like). We identified six additional putative *CMF* genes from the Morex V2 annotation (*15*), including two HvCMF4-like proteins (HvCMF4L1 and HvCMF4L2) that likely arose from partial gene duplication after divergence of the Triticeae (fig. S7, A and B). We found that gene deletion at *HvCMF4L2* (fig. S7C) in the barley pan-genome (*16*) and loss-of-function alleles for *HvCMF4L1* did not (or mildly) influence spikelet degeneration and death. Notably, spikelet death of *hvcmf4l1*/*hvcmf4* double mutants was similar to that of *hvcmf4* single mutants (fig. S7, D and E), suggesting a probable neofunctionalization of *HvCMF4L1*. A phylogenetic tree of HvCMF4 homologs in gramineous and eudicot species showed that CMF4 appears to be grass specific (fig. S8A). Notably, the molecular functions of most HvCMF4 homologs are unknown: Some CMFs are involved in starch synthesis (CMF6a clade) or activation of sugar-inducible genes (*17*, *18*). Paralogs of CMF4 in the Pooideae (wheat, barley, rye, and *Brachypodium*) are structurally separable from other subfamilies such as Ehrhartoideae (rice) and Panicoideae (maize, sorghum, and millet). We inspected immature inflorescences in representatives of these taxa and found that early inflorescence greening was only observed in members of the Pooideae species but not in rice or sorghum (fig. S8B). This indicates that early inflorescence greening may be an innovation during the evolution of Pooideae species and that neofunctionalization of CMF4 has been essential for it.

**Fig. 3.**
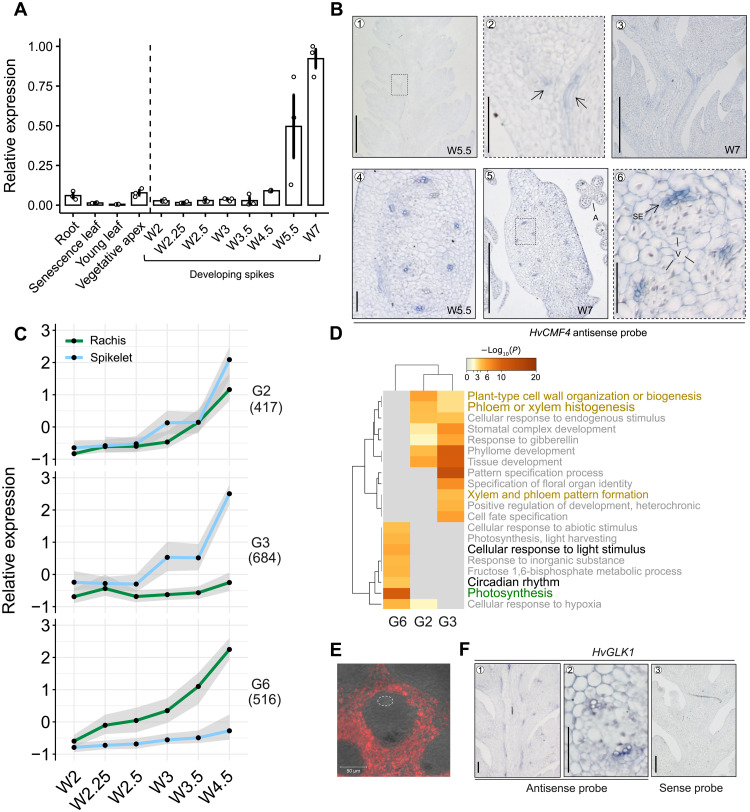
*HvCMF4* expression pattern and the coupled transcriptional programs. (**A**) Relative expression (RT-qPCR) of *HvCMF4* in different tissues. Data are shown as means ± SEM; *n* = 3 biological replicates; each replicate was the pool of 5 to 20 individuals. (**B**) ISH of *HvCMF4* in developing spikes. Spike longitudinal (B1 to B3) or transverse sections (B4 to B6) are shown. B2 and B6 are zoom-in views from B1 and B5 dashed frames. Arrows indicate the junctional region of a spikelet and the main rachis, where spikelet-rachis intervascular connection is going to be established. Putative diffuse vascular bundles (left arrow) of the spikelet axis and enlarged vascular bundles (right arrow) of the rachis are indicated. A, anther; SE, sieve element; V, vasculature. (**C**) The coexpressed clusters identified on the basis of 4783 DYGs (see also fig. S10). Shown are three clusters with peak expression at W4.5 stage in rachis or spikelet. Gene number from each cluster is given below each cluster. (**D**) The enriched GO terms of genes from the three clusters in (C). (**E**) Chlorophyll autofluorescence imaging from a transverse section of BW spike at W4.5 stage. White dashed circle highlights cells where *HvCMF4* is expressed. (**F**) ISH of *HvGLK1* from longitudinal (F1) and transverse (F2) sections of BW spikes at W5.5 stage. Scale bars, 200 μm (B1, B3, and B5), 50 μm (B2, B4, and B6), 50 μm (E and F1 and F3), and 20 μm (F2).

*HvCMF4* expression was limited to developing spikes at W4.5 stage and later, peaked at W7 stage (Fig. 3A), the time when developmental defects in *tst2.b* became manifested. Higher *HvCMF4* transcription was detected in the apical parts of the spike after W4.5 stage (fig. S9A). mRNA in situ hybridization (ISH) signals confined *HvCMF4* expression to spike vasculatures in all tissue types assayed, including the rachis, spikelet, lemma, anther, and the spikelet-rachis junction (Fig. 3B and fig. S9B). Specifically, *HvCMF4* mRNA was detected in the sieve element of phloem cells, a specialized cell type that functions in nutrient and signal transduction. Furthermore, we found HvCMF4-YFP (yellow fluorescent protein) fusion proteins targeted to nuclear bodies (fig. S9C), which are specialized subnuclear structures that emerged as key players in cellular signal transduction (*19*). To understand the molecular signatures coupled with the transcriptional activation of *HvCMF4* around W4.5, we investigated a transcriptome atlas of developing barley spikes that we had previously compiled and which spans six developmental stages from W2 until W4.5 (fig. S10, A and B) (*20*). We identified 4783 dynamically expressed genes (DYGs) caused by developmental stages or tissue types (spikelets versus rachis) and further grouped them into eight sequential clusters (G1 to G8) (fig. S10C and Materials and Methods), which then fell into two sequential transcriptional waves intersecting at the W3 stage, and two relatively stable clusters associated with spikelet (G4) and rachis (G5). Gene ontology (GO) analysis for the first wave, including G1, G7, and G8, was consistent with the spikelet initiation process, showing enrichment of flower histogenesis terms (fig. S10D, right). The second wave (G2, G3, and G6), which showed the highest expression at W4.5 stage and thus entrained biological processes *HvCMF4* was associated with, was enriched in processes related to circadian rhythm, photosynthesis, and vascular patterning (Fig. 3D). Moreover, G4 and G5 (two stable clusters), which were specific to rachis and spikelet, were enriched in terms related to flower development and photosynthesis (fig. S10D, left). Greening first appears at the main rachis and then extends to the spikelet close to W4.5 stage (fig. S10E), suggesting a functional relevance of the second transcriptional wave for spikelet greening, reinforcing the idea that one of HvCMF4’s functionalities is to control spikelet greening. We found that the rachis and spikelet vascular bundles were devoid of chlorophyll autofluorescence signals (Fig. 3E), indicating a likely cell nonautonomous regulation of spike greening via vascular signaling conferred by *HvCMF4*. To follow this lead, we used ISH to detect transcript localizations of selected transcription factors (TFs), including *VASCULAR-RELATED NAC-DOMAIN PROTEIN1* (*HvVND1*; for xylem development), *ALTERED PHLOEM DEVELOPMENT* (*HvAPL*; for phloem development), or *GOLDEN2-LIKE 1* (*HvGLK1*; for chloroplast development) (*21*, *22*). Expression of all selected genes peaked at W4.5 and was enriched in the spike vasculature (Fig. 3F and fig. S11). Thus, *HvCMF4* may deliver phloem-derived molecular signals to control spike greening and spikelet fate.

### *HvCMF4* orchestrates transcriptome reprogramming to fuel spikelet growth

We conducted transcriptome studies of spike sections and anther at developmentally defined stages after W4.5 in BW and *tst2.b* (fig. S12A). A principal components analysis (PCA) revealed trajectories corresponding to developmental gradient (PC1: apical, central, and basal) and growth stage (PC2: W4.5, W5.5, and W7) (fig. S12B), coinciding with the dead versus viable spikelets in *tst2.b* and BW and their localization. We identified 14,216 DYGs from the spike sections whose expression covaried with at least one of the three factors: genotype (BW versus *tst2.b*), developmental gradient, and growth stage. Among these, 8610 DYGs were affected by the allelic status of *HvCMF4* (genotype), of which 8239 (~95%) were simultaneously influenced by at least one of the other two factors. The number of DYGs affected by *HvCMF4*’s allelic status coincided with the basipetal progression of spikelet degeneration and death (fig. S12, C and D), suggesting that loss of *HvCMF4* function rearranges the transcriptional programs associated with the spikelet developmental gradient.

**Fig. 4.**
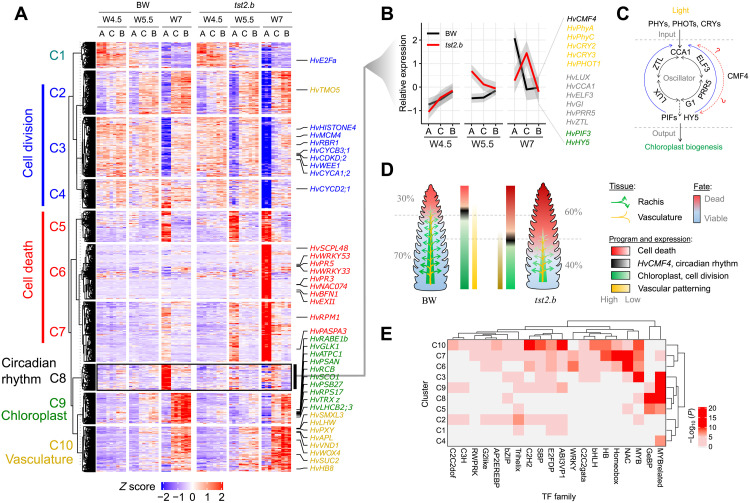
*HvCMF4* orchestrates transcriptome reprogramming after W4.5 stage. (**A** and **B**) Coexpressed gene clusters of spike sections across three developmental stages. Selected overrepresented functional classification terms from the clusters and the representative barley orthologs are shown on the left and right sides of the heatmap in (A). (B) highlights the expression pattern of *HvCMF4*-coexpressed gene cluster (C8), which includes light receptors (orange), circadian clock genes (gray), and the light signaling TFs (green). “A,” “C,” and “B” correspond to “apical,” “central,” and “basal,” respectively. (**C**) A fuzzy model, based on (*31*, *35*, *81*), summarizes a molecular signature wherein an input signal (light) transmitted by photoreceptors to the circadian clock (oscillator) can lead to chloroplast biogenesis (output), a process whose peak expression is synchronized with the spikelet degeneration and death process. Circle arrows represent the interlocked transcriptional-translational feedback loop involving the clock, input, and output. HvCMF4 may directly or indirectly modulate the process at different regulatory nodes. (**D**) Proposed biological implications for spikelet survival, greening, and vascularization due to the loss of *HvCMF4*. (**E**) Enriched motifs from upstream of each of the 10 clusters from spike sections. Associated TF families with *P* < 0.001 in at least two clusters are hierarchically clustered.

**Fig. 5.**
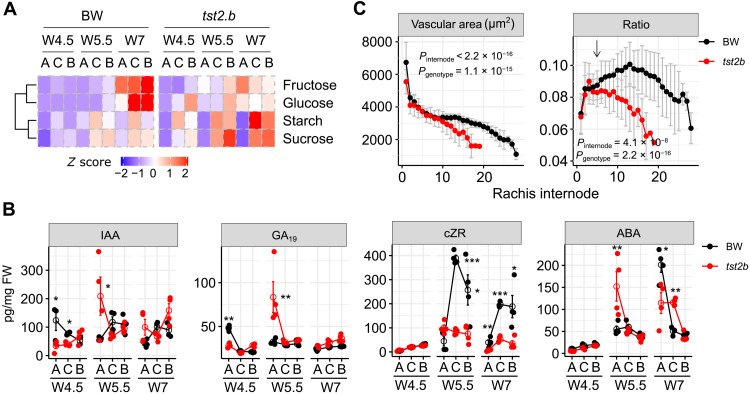
Comparison of vascular patterning, phytohormones, and photosynthates in BW and *tst2.b*. (**A** and **B**) Photosynthate (A) and phytohormone (B) profiling from different spike sections across three developmental stages. Heatmap is shown as the scaled mean value of each product. Data in (B) are shown as means ± SEM, *n* = 4 or 5 biological replicates. IAA, indole-3-acetic acid; GA_19_, gibberellic acid; cZR, *cis*-zeatin 9-riboside (cytokinin); ABA, abscisic acid. Raw data are given in table S7. See also fig. S16G for other derivatives. Significance levels are determined from two-tailed Student’s *t* test. *P < 0.05; **P < 0.01; ***P < 0.001. FW, Fresh Weight. (**C**) Vascular size comparison at the tipping stage. Ratio is determined from the division of vascular area by total tissue area. Arrow points to the junction between sterile and fertile spikelets. Data are shown as means ± SD. *P* values from internode and genotypic effects are determined with two-way analysis of variance (ANOVA), *n* = 10 (BW) or 9 (*tst2.b*) biological replicates.

Of the 14,216 DYGs from spike sections, we retrieved 10 coexpression clusters (C1 to C10) (Fig. 4A and fig. S13A). Expression of genes involved in photosynthesis-related pathways (represented by C9, e.g., for plastid division and pigmentation) recapitulated the greening process in BW and was down-regulated in *tst2.b* at W4.5 stage and onward (Fig. 4, A and D, and figs. S13B and S14, A to C). Those genes were also highly and stably expressed before *HvCMF4* expression was detectable, indicating a sequential transcriptional control for chloroplast biogenesis in the rachis, similarly found in wheat leaves (*23*). We validated the down-regulated expression of a *LIGHT-HARVESTING CHLOROPHYLL B–BINDING* gene (*HvLHCB2;3*) in the *tst2.b* rachis and spikelet by ISH (fig. S14, D and E). Since meristem maintenance and development require nutrient signaling derived from photosynthesis-related processes (*24*), we therefore hypothesized that the early appearance of rachis greening might serve as an energy source to sustain floral meristem growth. This notion was supported by our previous analysis of our transcriptome spike atlas dataset, in which genes for starch metabolism or sugar transporters were highly expressed, either in the rachis or in the spikelet. Consistent with this, we found that genes for central carbon and energy metabolism pathways exerted lower expression in *tst2.b* apical sections of spikes at W5.5 and W7 stages compared with BW (fig. S14, B and C, bottom). Moreover, metabolite profiling revealed that *tst2.b* spikes tended to overaccumulate starch and sucrose in the central and basal parts at W5.5 and W7 stages compared with BW; consequently, fructose and glucose were less abundant (Fig. 5A). We found that apical spikelets from BW lacking rachis greening, and thus most likely deprived of energy supply, had lower expression of cell cycle genes (represented by C2 to C4) starting from W5.5 stage and subsequently activated cell death genes (represented by C5 to C7) at W7 stage. In the case of *tst2.b*, however, expression of both cell cycle and cell death genes displayed heterochronic and heterotopic shifts, leading to premature spikelet degeneration and death (Fig. 4, A and D, and fig. S15, A and B). ISH of a cell cycle marker gene (*HISTONE4*) and a cell death gene (*SCPL48*) (*25*) supported this finding (fig. S15, C and D). In line with these processes, we found that levels of the stress hormone abscisic acid and its derivatives phaseic acid and dihydrophaseic acid were synchronized with the progressing degeneration process in both BW and *tst2.b* (Fig. 5B and fig. S16G). Overall, these results suggest that photosynthates from the rachis fuel the growth of developing spikelets during early growth stages. We conclude that plastidial defects in *tst2.b* prevent starch remobilization and utilization destined to support the energy demand that drives meristematic growth in developing heterotrophic floral tissues.

Similar to the cluster for chloroplast development (i.e., C9; see above), the expression of genes for vascular patterning (represented by C10) increased during development and declined acropetally along the spike in both BW and *tst2.b*, which was opposite to the direction of degeneration, implying that a reduced vascularization toward the distal end of a spike may account for apical spikelet degeneration and death. Unexpectedly, we found that genes from C10 were more highly expressed in the central and basal parts of the *tst2.b* spikes (Fig. 4, A and D), indicating a positive feedback control for vascular patterning likely due to intensified tip degeneration. We tested this by comparing the vascular size in BW and *tst2.b* rachises and found that vascular sizes in both genotypes displayed an acropetally descending pattern; however, vascular sizes in the spike apex of *tst2.b* were much smaller, while the overall rachis area remained largely unchanged (Fig. 5C and fig. S16, A to C), suggesting a vascular-specific developmental arrest in the mutant. Notably, the spatial pattern of the vascular area per rachis unit (ratio of dividing the vascular area by the total rachis area) in BW was positively correlated with the known proximal-distal reduction of grain sizes from the central spike toward the apical and basal spike parts (*26*), which is commonly seen in Triticeae. Such a pattern was greatly disturbed in *tst2.b* and coupled with a reduction in grain size (Fig. 5C and fig. S16D), suggesting that the balanced establishment and growth of the vasculature along the spike are key for proper spikelet and grain development. Since vascular size is driven by periclinal divisions controlled by cytokinin with auxin as an inhibitory feedback (*21*), we detected lower cytokinin levels (iPR and cZR) but higher auxin levels (indole-3-acetic acid) in *tst2.b* spikes (Fig. 5C and fig. S16G). We thus hypothesized that *HvCMF4* may control vascular morphogenesis via canonical hormonal signals and that the *HvCMF4*-expressing protophloem sieve elements likely represent meristematic phloem cells. Expression of barley *WUSCHEL-RELATED HOMEOBOX 4* responsible for vascular proliferation (*27*) was highly enriched in the same tissue type as *HvCMF4* (fig. S16, E and F).

Our phenotypic analysis suggested that while apical degeneration involved cell degradation of whole organs such as spikelet, inflorescence meristem, and rachis, grain abortion in *tst2.b*, however, was mainly due to anther defects that failed to pass W7 stage. To further address how *HvCMF4* may contribute to anther greening and development, we analyzed anther transcriptomic data collected from W8 and W9, during which anther greening became evident. We found that chloroplasts are important for anther development (fig. S17), which was supported by the photosynthesis inhibitor treatment assays and the TEM observation. Likewise, insufficient energy supply due to lack of greening might have suppressed cell division, which resulted in anther developmental defects. Because *HvCMF4* transcripts were only detected in anther vasculature, we conclude that vascular signals in the anther are essential for anther greening and subsequent pollen maturation. Together, these findings suggest that the protophloem sieve elements could serve multiple purposes or adopt several cell states, i.e., directing vessel and plastid differentiation to control spikelet growth and survival, a proposed function clearly beyond its textbook role in carbon loading (*28*).

### *HvCMF4* connects vascular circadian clock, thereby maintaining spikelet growth

**Fig. 6.**
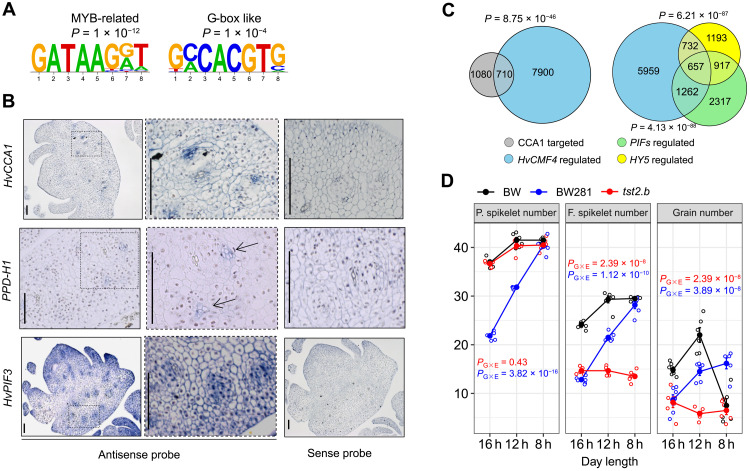
HvCMF4 may regulate spikelet growth via the light signaling pathway that entrains the circadian clock. (**A**) Two enriched motifs from the *HvCMF4*-coexpressed cluster (C8). (**B**) ISH of barley *PPD-H1*, *CCA1*, and *PIF3* orthologs in spike transverse sections at W5.5 stage. Scale bars, 50 μm. (**C**) Venn diagrams showing overlaps of barley orthologs of *Arabidopsis* CCA1–targeted, *PIF*-, and *HY5*-regulated genes with *HvCMF4*-regulated genes from spike sections. *P* values are defined on the basis of Fisher’s exact test. (**D**) Effect of day length on potential (P.) spikelet number, final (F.) spikelet number, and grain number variations. Data are shown as means ± SEM, *n* = 6. The significance of genotype (G) × environment (E) interaction is determined by ANOVA.

To interrogate potential signaling pathways from the spike vasculature that control tissue greening, we investigated the enriched DNA motifs [false discovery rate (FDR)–adjusted *P* ≤ 0.001] from each of the 10 gene clusters identified with spike sections (table S12). TFs, such as WRKY, NAC, and Homeobox families that are involved in stress responses or cell death, were highly enriched in C6 and C7 (activated in apical sections at W5.5 and W7) (Fig. 4E), which was consistent with the tip degeneration phenotypes and suggested a sufficient specificity of our analysis. Other enriched motifs include a “GATAA” sequence from C3, C8, and C9 and a G-box motif (CACGTG) from C7, C8, and C10 (Fig. 6A). The GATAA sequence resembled the motif bound by MYB-related TFs such as CIRCADIAN CLOCK ASSOCIATED 1 (CCA1) (*29*), while the G-box was commonly recognized by circadian oscillators and output TFs, such as PHYTOCHROME-INTERACTING FACTORS (PIFs) and ELONGATED HYPOCOTYL 5 (HY5). Since chloroplast biogenesis is closely integrated with light signals that entrain the circadian clock and outputs (*30*), we hypothesized that *HvCMF4* might integrate the circadian system to orchestrate the transcriptional processes for chloroplast development and spikelet survival (Fig. 4C). In support of this notion, we found that *HvCMF4* was coexpressed with C8 genes (see Materials and Methods), whose peak expression was synchronized with the degeneration and death process and comprised core components of circadian clock genes, light receptors, and output TFs (Fig. 4, A and B), suggesting that they might act in the same process. Reverse transcription quantitative polymerase chain reaction (RT-qPCR) revealed that *HvCMF4*, together with clock genes *HvCCA1* and *HvTOC1* and two clock output genes *HvPIF3* and *HvLHCB2;3* (*LIGHT-HARVESTING CHLOROPHYLL A/B–BINDING PROTEIN2;3*), showed oscillating expression patterns in BW spike sections at W5.5 stage in a diel cycle (fig. S18A). Expression of *HvCCA1* peaked at 3 hours after light, whereas *HvTOC1* was antiphasic to *HvCCA1*. Expression of *HvCMF4*, *HvPIF3*, and *HvLHCB2;3* peaked at 6 hours after light, suggesting a coherent regulatory link. Noticeably, except for *HvTOC1*, cyclic amplitudes of the other five genes examined differed between the two spike sections, implying the existence of a vascular-specific clock within the spike. We found that transcripts of clock genes *HvCCA1* and *PPD-H1* and an output gene, *HvPIF3*, colocalized in the same cell type as *HvCMF4*, despite *HvPIF3* being more broadly expressed (Fig. 6B). Because most of the circadian clock and output pathways are conserved between grasses and *Arabidopsis* (*31*), we used orthologs of downstream targets of *Arabidopsis CCA1* (*32*), *PIFs* (*33*), and *HY5* (*34*) as proxies to test whether they were commonly regulated by *HvCMF4*, for which we found a large degree of overlap with each set of orthologs (Fig. 6C). Noticeably, in addition to lack of greening, we observed early elongated rachis internodes and elevated gibberellin levels in the *hvcmf4* mutants (Fig. 4D and fig. S6E), a symptom mimicking skotomorphogenesis mediated by the HY5-PIF regulatory module (*35*). We hypothesized that an altered circadian clock regime in *tst2.b* may reduce its sensitivity to light-mediated chloroplast biosynthesis and survival. We found that both spikelet survival and spike chlorophyll content from two short-period mutants were reduced under long-day conditions (16 hours) compared with BW (fig. S18, B to D). Furthermore, spikelet survival in BW281, one of the short-period mutants carrying the *PPD-H1*–sensitive allele, was fully restored under short-day conditions (8 hours) (Fig. 6D and fig. S19A). In addition, shifting from 16 to 8 hours largely restored the expression of chloroplast and vascular genes from the spikes of another sensitive *PPD-H1* carrier (fig. S19, D and E) (*36*). Loss of *HvCMF4*, however, abolished the photoperiod sensitivity for the final spikelet number compared with BW, although both potential spikelet number and days to heading remained sensitive (Fig. 6D and fig. S19, A and B). In summary, the photoperiod sensitivity for spikelet growth is reduced by the loss of *HvCMF4* but is enhanced by a functional *PPD-H1* allele compared with the wild-type BW. These results indicate that *HvCMF4* can act as a sensory factor under environmental fluctuations (e.g., light) to coordinate spikelet initiation and growth via signals from the vascular-localized circadian clock, a robust mechanism that, in turn, entrains circadian regulation in other nonvascularized floral tissues (*37*).

### *HvCMF4* natural variations can be exploited to modulate spikelet survival

**Fig. 7.**
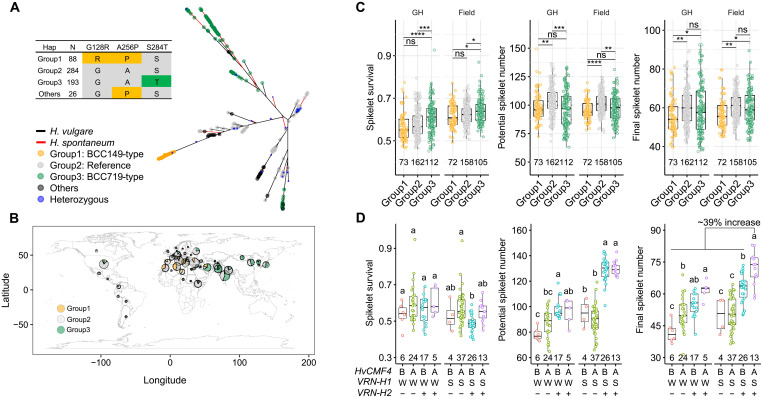
Natural *HvCMF4* variants increase spikelet number by enhancing spikelet survival. (**A**) An unrooted neighbor-joining phylogenic tree of the *HvCMF4* locus. SNPs (138) within 3 kb upstream until 1 kb downstream of *HvCMF4* from 102 wild barleys (*Hordeum spontaneum*) and 518 domesticated barleys (*Hordeum vulgare*) are considered. Haplotypes are colored according to the three amino acid substitutions. (**B**) Geographic frequency distribution of the three major groups of the 518 domestication barleys. Capital latitude and longitude are used to coordinate in the map. (**C**) Phenotypic comparisons among the three major haplotype groups (minor allele frequency ≥ 0.05). Significance levels are determined from two-tailed Student’s *t* test. *P < 0.05; **P < 0.01; ***P < 0.001; ****P < 0.0001. ns, not significant. (**D**) Gene pyramiding from a DH population. “B,” “W,” and “+” represent alleles from BCC149; “A,” “S,” and “−” represent alleles from BCC719. Letters above the boxplot represent statistical significance from ANOVA followed by Tukey’s post hoc test.

We next interrogated natural sequence variation in *HvCMF4* and their contributions to spikelet survival and spikelet number. Haplotype analysis considering single-nucleotide polymorphisms (SNPs) from the *HvCMF4* locus revealed signs of diversifying selection. SNPs that resulted in missense mutations showed different geographical distributions (Fig. 7, A and B). While potential spikelet number was similar, the BCC719 type (or group 3, mostly from Eastern countries) clearly outperformed the BCC149 type (or group 1, mostly from Western countries) in terms of spikelet survival and spikelet number based on phenotypic data collected from both controlled greenhouse conditions and field environments (Fig. 7C) (*38*). Therefore, our results suggest that natural *HvCMF4* variations influence spikelet survival and thus spikelet number.

Next, we asked to what extent the favorable *HvCMF4*^BCC719^ allele can potentially benefit barley yields. We tested this by isolating eight allelic combinations of *HvCMF4*, *VRN-H1*, and *VRN-H2* from the 130 lines of the BCC719 × BBC149 doubled-haploid (DH) population by PCR genotyping. We found that the *VRN-H2*/*VRN-H1* epistatic module increased the potential spikelet number and spikelet number by ~43 and ~21%, respectively (fig. S1E). When combined with the *HvCMF4*^BCC719^ allele, the positive effect on spikelet number was elevated from ~21 to ~39%, while the potential spikelet number remained unchanged (Fig. 7D). Therefore, different sets of allelic variants associated with either the number of initiated or survived spikelets are stackable, facilitating selective breeding.

## DISCUSSION

Mechanistically, we show that *HvCMF4* acts in the spike vasculature to control organ greening via circadian regulation. CCT motif family genes are well known for their control of flowering time and floral initiation, many of which are expressed in the vasculature (*39*–*41*), implying an ancestral character of vascular functioning. It has been established that the vascular-localized circadian clock can robustly control the physiological response of neighboring cells, e.g., in *Arabidopsis* cotyledons via circadian entrainment (*37*). It is therefore conceivable that the initiation and the growth phase are both controlled by the circadian clock; thus, clock signaling from the vasculature of the developing inflorescence may arise as a major coordinator of the growth-death tradeoff during the growth phase. Potential spikelet number in both BW and *tst2.b* remains sensitive to day length, but increased final spikelet number is only observed in BW and BW281 (Fig. 6D). Evolutionarily, the subfamily Pooideae within grasses, including wheat, barley, rye, and *Brachypodium*, has been ecologically dominant in seasonally colder climates to temperate areas (*42*). Given that high chlorophyll levels positively regulate cold tolerance (*43*), it is tempting to speculate that the evolution of CMF4 might have helped Pooideae to adapt to colder climates by protecting their reproductive meristems during cold periods with more chlorophyll. In this scenario, *HvCMF4* may function as a thermosensor to regulate chloroplast development and reproductive success. This idea can be supported by the findings that photosensory and thermosensory pathways are highly interconnected to control development (*8*, *44*, *45*). Another paralogous pair, i.e., *HvCMF3*/*7* encoding plastid-localized proteins, has recently been reported to control leaf greening (*46*, *47*) but with little or no effects on spike morphology (fig. S19, F and G), indicating distinct mechanisms for the control of spike and leaf greening.

Our study evokes an alternative avenue for boosting grain yield, highlighting the possibility of increasing grain number not only by gaining more floral primordia but also by convoying them until maturity. In a world of climate change and enhanced demand for food, insights into how plants cope with ever-changing environments to guarantee reproductive success may help us to design future crops with improved flexibilities in the face of erratic stresses.

## MATERIALS AND METHODS

### Plant materials, growth conditions, and phenotyping

The 358 six-rowed spring barleys (table S1) were selected from the Federal Ex-situ Gene Bank hosted at Leibniz Institute of Plant Genetics and Crop Plant Research (IPK) (*9*). Selections were initially made to maintain the maximum genetic diversity of the whole population informed by a PCA. PCR genotyping was conducted to select lines that carry the sensitive allele of *Photoperiod-H1* (*PPD-H1*) based on SNP22 located in the CCT domain (*48*). The 130 DH lines (table S3) were produced by crossing BCC149 (winter barley) and BCC719 (spring barley); both were selected from a previous barley collection used for association studies (*49*) and were ordered from IPK Gene Bank. Barley mutants including *tst2.b* (BW883), three *short spike* mutants (*sp1*, *sp20*, and *sp28*), and early maturity mutants (*eam* and *mat*) were ordered from the NordGen Seed Bank. Candidates for *eam5.x* (*HvPHYTOCHROME C*), *eam10.m* (*HvLUX*), and *mat-c* (*HvCENTRORADIALIS*) have been reported before (*50*–*52*). Barley *hvcmf3* and *hvcmf7* were reported elsewhere (*46*, *47*). To reduce background introgressions from the originally mutagenized genotype (cv. Ackermann’s Donaria), we further backcrossed BW883 (*tst2.b*) to BW to generate BC_6_F_4–5_ plants for most of the studies. All barley mutants used in this study are given in table S2. Cultivar Golden Promise (two-rowed, spring type) was used as the recipient for genetic transformation.

Most experiments were conducted under greenhouse conditions (photoperiod: 16 hours/8 hours, light/dark; temperature: 20°/16°C, light/dark) between 2018 and 2022 at IPK. For experiments that required phenotyping of mature spikes, barley grains were germinated in a 96-well planting tray for 2 weeks, vernalized at 4°C for 4 weeks, acclimatized at 15°C for a week, and lastly potted into 9- or 14-cm^2^ pots until maturity. In experiments that required dissection of immature spikes at early developmental stages, such as transcriptomic study and chlorophyll, hormone, and photosynthetic product profiling, barley grains were directly germinated in 24-well planting trays or 9-cm^2^ pots until sample collection without further potting. For sample collection of the transcriptomic study (66 samples), we grow plants under the same controlled phyto-chamber condition as in (*20*) (photoperiod: 12 hours/12 hours, light/dark; temperature: 12°/8°C, light/dark). We conducted one candidate gene (*HvCMF4*) mapping experiment and one for phenotyping the *short spike* mutants under field conditions in 2019 and 2021, respectively, in which plants were grown in rows with a 15-cm distance between rows and with eight plants in each row.

All phenotypic data were collected from the main culm. Potential spikelet number was determined according to (*53*), and heading date was determined when half of the spike appeared from the leaf sheath. Final spikelet number was counted between heading and grain filling stage, and grain number was counted after harvesting. Spikelet survival was deduced by dividing the final spikelet number by potential spikelet. Four to five randomly selected plants were used for every trait to phenotype the 358 barleys and the 130 DH populations. Because of greenhouse space limitation, we conducted two independent experiments to phenotype the full set of the 358 barleys (experiment #1, 128 accessions; experiment #2, 256 accessions), with 25 accessions overlapping between each experiment (1 accession was removed for analysis because of admixture). Both experiments were conducted in the same greenhouse. The timeline for experiment #1 was from December 2018 to May 2019, and the timeline for experiment#2 was from December 2019 to May 2020. Barley accessions were grown in a completely randomized design to reduce residual variances. We obtained the individual phenotypic variance components by using a linear mixed-effect model.

For the 130 DH lines, a single experiment was conducted from May to October 2019 in the same glasshouse. For spikelet-tracking experiments, barley mutants and corresponding wild types were dissected every 2 to 3 days under a light-sheet microscope. To compare meristem size (length and width), images taken by AxioVision (SE64 Rel. 4.9.1) were directly measured in the same software. All phenotypic data analyses were performed in R (R-3.6.1).

### BCC149 × BCC719 DH population

Generation of the DH population was done according to (*54*). Linkage map construction was done from the genotyping-by-sequencing data of the 130 DH lines (table S3). QTL analysis was done with QTL ICiMapping v4.2 (*55*). Both inclusive composite interval mapping (ICIM-ADD method) and single-marker analysis were used to detect QTL. Population type was set as F1DH (F_1_-derived DHs). Permutation tests (1000 times) were used to determine the significance threshold at *P* < 0.05. QTL results are summarized in table S4.

### Treatments

The photosynthesis inhibitors, norflurazon and lincomycin, used were found to disrupt chloroplast integrity by reducing the expression of nuclear genes for chloroplast proteins (NGCPs) (*56*–*58*). Since the first spike morphological difference (i.e., greening) between *tst2.b* and BW appeared at ~W4.5 and onward, our spike transcriptome analysis suggested that many of the NGCPs that were activated close to W3.5 (stamen primordium stage) were down-regulated in *tst2.b* spikes compared with BW; besides, it has been suggested that the molecular signatures underlying spikelet initiation (which determine the potential spikelet number) may be established before W3.5 (*59*); consequently, molecular and cellular changes imposed by treatments after W3.5 may not have major impacts on the potential spikelet number but rather on spikelet development; we therefore performed our treatment assay starting from W3.5 of spike development. Barley treatment assay was performed according to a previous report (*60*) with minor modifications. Briefly, stock solutions for norflurazon (10 mM; Sigma-Aldrich) and lincomycin (100 mM; Sigma-Aldrich) were prepared by dissolving in 95% ethanol and H_2_O, respectively, and diluted in water containing 0.02% Silwet L-77 (PlantMedia) before application. All treatments were done by applying 30 ml of working solution to BW plants grown in 9-cm^2^ pots. Treatments were applied daily starting from W3.5 for the first 2 weeks and subsequently every 2 days until the completion of the experiment.

### Gene fine mapping

We used *tst2.b* for gene fine mapping as (i) it was a backcrossed material that contained reduced background mutations compared with the other three independent *short spike* mutants, (ii) it facilitated mapping marker development within the introgression when backcrossed with BW, and (iii) compared with *tst2.b*, the three *short spike* mutants were completely sterile under greenhouse conditions. To facilitate marker development and identification of causal mutation, we sequenced (~10 × coverage) BW883 (*tst2.b*) and Ackermann’s Donaria. Read alignment and variant detection were done according to (*16*). SNPs detected from the comparisons were converted to restriction enzyme–based CAPS with SNP2CAPS algorithm (http://pgrc.ipk-gatersleben.de/snp2caps/). We identified six putative introgressions from the original mutagenesis of Ackermann’s Donaria in *tst2.b* through pairwise comparisons of the variants between each of the two genotypes.

We adopted a map-based cloning strategy to study the genetic basis that underpins the *tst2.b* mutant phenotype by generating two F_2_ populations from crosses of *tst2.b* with BW (108 individuals) and Ackermann’s Donaria (1205 individuals, including 588 grown under field conditions), respectively. The segregating pattern for spikelet number (high versus low) and spike length (long versus short) in the F_2_ populations fitted into a ratio closed to 3:1, suggesting a monogenic recessive mutation in *tst2.b*. We initially mapped the causal mutation to the long arm of 4H between markers MAP4H_39 (physical position: 605,090,607) and MAP4H_42 (physical position: 607,879,736) with the 108 individuals from *tst2.b* × BW. By screening all types of recombinants with nine additional markers evenly distributed within this interval and subsequently phenotyping the offspring (*F*_3–4_), we narrowed down the causal mutation to a 750-kb interval based on Morex reference V2 (table S5). Surveying all types of mutations in this interval from the above-called variants identified a 4-bp deletion in *HvCMF4* gene that is specific to *tst2.b*. This 4-bp deletion gave rise to a codominant CAPS marker, and we used it to genotype the 1205 individuals from *tst2.b* × Ackermann’s Donaria and found it well cosegregating with the phenotypes. Primers used for recombinant screening were given in table S15.

### Tissue collection, RNA extraction, and RT-qPCR

In this study, we used BW for the RT-qPCR assay. For testing rhythmic gene expression in immature spikes, spike tissues at W5.5 stage were collected every 4 hours across a 24-hour period, and five samples were pooled to make one replicate. For investigating gene expression pattern in diverse organs, tissues were always collected at the same time slot (11:00 a.m. to 2:00 p.m.) during the day, and 5 to 20 samples were pooled to make one replicate, depending on the tissue amounts. Total RNA was extracted with TRIzol (Invitrogen) and precipitated with 2-propanol. Genomic DNA residues were removed with deoxyribonuclease I (NEB, M0303L), and first-strand complementary DNA (cDNA) was synthesized from total RNA with the SuperScript III Reverse Transcriptase Kit (Invitrogen, 18080-051). Real-time PCR was performed with SYBR Green Master Mix (Thermo Fisher Scientific, A46112) under the ABI Prism 7900HT sequence detection system (Applied Biosystems). For each RT-qPCR, three technical replicates were performed for each of the three biological replicates. Barley *HvActin* was used for normalization. Primers for RT-qPCR are given in table S15.

### Cas9-mediated knockout

Guide RNA (gRNA) design was based on the genome sequence of barley cv. “Golden Promise.” Selection of target motifs was conducted (i) using CRISPR-P 2.0 (http://crispr.hzau.edu.cn/CRISPR2/), (ii) ensuring that the cognate gRNAs are specific to the target gene via blastn and (iii) form suitable secondary structures as predicted with the RNAfold webserver (http://rna.tbi.univie.ac.at/cgi-bin/RNAWebSuite/RNAfold.cgi).

The construct targeting to HvCMF4 (three guides) and HvCMF4L1 (one guide) was generated using the hierarchical Golden Gate cloning strategy (*61*). Oligos (table S15) with complementary target sequences and overhangs for cloning into Bsa I restriction sites were annealed and cloned into Bsa I–linearized vectors pIK1 to pIK4 containing OsU3 promoter. The resultant four vectors containing four gRNA units were digested with Esp 3I enzyme and assembled into one single vector pIK19. To combine the multiple gRNA fragments with the Cas9 expression unit (pIK83) and an auxiliary unit vector (pIK155) into vector pIK22, Bsa I restriction digest followed by ligation was used. Last, all expression units were mobilized into binary vector p6-d35S-TE9 (DNA Cloning Service) via Sfi I restriction digest. Resultant vector was *Agrobacterium*-mediated delivered into immature embryos of barley cv. Golden Promise (*62*). Following a series of hygromycin selection and regeneration, transgenic plantlets were screened for the presence/absence of transferred DNA insertion. Target amplicons were then subjected to Sanger sequencing for detection of mutations.

### Subcellular localization

Full-length coding region of *HvCMF4* was PCR-amplified from wild-type BW spikes, directionally cloned into the pENTR/D-TOPO entry vector (Thermo Fisher Scientific, K240020), and then released and ligased into the transient expression vector pIPKTA49 fused with C-terminal YFP (https://figshare.com/articles/dataset/Sequence_information_of_pIPKTA48_and_pIPKTA49/6652415/1). The resultant vector was delivered into barley leaf epidermis via microparticle bombardment, followed by 12 hours of incubation, and then scanned for fluorescence signals under an LSM780 confocal laser scanning microscope (Carl Zeiss MicroImaging). Primers for vector construction for subcellular localization are given in table S15.

### RNA ISH

For probe preparation, gene-specific fragments (300 to 500 bp) were PCR-amplified from the total cDNA of BW spikes and cloned into the pGEM-T cloning vector. After sequencing validation, vectors were used as templates for the preparation of sense (negative control) and antisense probes. A fusion primer set containing a 20-bp T7 promoter sequence (5′-TAATACGACTCACTATAGGG-3′) before the forward primers of sense probes or reversed primer of antisense probes was used to generate templates for in vitro reverse transcription with T7 RNA polymerase. For ISH, spikes were hand-dissected and fixed overnight with FAA (50% ethanol, 5% acetic acid, and 3.7% formaldehyde) at 4°C, followed by dehydration with series of ethanol (50, 70, 85, 95, and 100%), and then embedded into Paraplast Plus (Kendall, Mansfield, MA). Sections (8 μm thick) were prepared on a microtome and mounted onto Superfrost plus slides. Tissue pretreatment, hybridization, washing, and coloration were performed as previously described (*63*). Primers for amplifying the probe sequences are given in table S15.

### Phylogeny

We reannotated barley CCT genes by blasting CCT domains from HvCMF4 (301 to 345 residues) and Pfam database (http://pfam.xfam.org/) (PF06203) against barley Morex V2 proteome (*e* value: 0.005). Full-length protein sequences from the retrieved genes were domain-annotated with hmmscan under Pfam database to identify additional domains such as B-box–type zinc finger domain (PF00643), response regulator receiver domain (PF00072), GATA zinc finger (PF00320), or tify domain (PF06200). Gaps present in ≥80% of the aligned sequences were removed. Phylogenetic trees were built with RAxML (*64*). We carried out rapid bootstrapping and best­scoring ML tree searching in the same run (-f a) with an extended majority rule (-# autoMRE). The resultant tree was visualized with Evolview v3 (*65*). During the analysis, we noticed two CMFs (HvCMF4L1 and HvCMF4L2) with high amino acid sequence identity (~95%) compared with HvCMF4, which may indicate gene duplication. To ascertain this, we aligned the genomic sequences from *HvCMF4L1*/*L2* against a ~5.5-kb genomic sequence surrounding *HvCMF4* under the Sequence Alignment Viewer (https://ncbi.nlm.nih.gov/projects/msaviewer/) and found that both *HvCMF4L1* and *HvCMF4L2* arose from a partial duplication (~2 kb, with ~97% identity) of the *HvCMF4* locus. We blasted the ~2-kb genomic sequences from both *HvCMF4L1* and *HvCMF4L2* against the remaining 19 barley reference genomes (*16*). While *HvCMF4L1* was present in all of the 19 reference genomes assayed, for *HvCMF4L2*, only 14 (including Golden Promise) of the 19 barley reference genomes showed a hit in the syntenic region. Notably, no hits were found in syntenic regions of wheat and rye genomes for both genes (*HvCMF4L1*/*L2*), suggesting that these duplication events happened after the divergence of these Triticeae species and that the *HvCMF4L2* duplication is younger than *HvCMF4L1*.

To construct a HvCMF4-specific phylogenetic tree, its full-length protein sequence was queried against proteomes from 14 species downloaded from Phytozome v12.1 (https://phytozome-next.jgi.doe.gov/), which included eight grasses and six eudicots. *E* value cutoff was set as 1 × 10^−10^, which retrieved 195 genes. After filtering genes without CCT domain or with the additional domains mentioned above, 186 were retained for building the tree. We followed the same procedure mentioned above to construct and visualize the tree. To ascertain true homology relationships with *HvCMF4* inferred from the tree, we blasted back genes from the *HvCMF4* clade against barley proteome.

### Population structure and haplotype analysis

SNPs within 3 kb upstream until 1 kb downstream of *HvCMF4* from the 358 spring barleys (109 in total) and previously reported 100 wild and 200 domesticated barleys (*16*) (104 in total) were merged. A neighbor-joining clustering based on the distance matrix was done with PHYLIP 3.68 (https://evolution.genetics.washington.edu/phylip). The output tree was visualized and annotated on the basis of the three missense mutations found between BCC149 and BCC719 with ggtree (*66*). *HvCMF4* haplotype information is given in table S1.

### Microscopic observations

For scanning electron microscopy, immature barley spikes were fixed in 50 mM cacodylate buffer (pH 7.2) containing 2% glutaraldehyde and 2% formaldehyde at 4°C. Samples were washed with distilled water and dehydrated in an ascending ethanol series and point-dried in a Bal-Tec critical point dryer (https://leica-microsystems.com). Dried specimens were gold-coated in an Edwards S150B sputter coater (http://edwardsvacuum.com) and examined in a Zeiss Gemini30 scanning electron microscope (https://zeiss.de) at an acceleration voltage of 10 kV. For TEM, samples were embedded in Spurr’s resin. Ultrathin sections (70 nm) were cut with a microtome (Leica Ultracut, Leica Microsystems, Bensheim, Germany) and subjected to TEM (Tecnai Sphera G2, FEI, Eindhoven, The Netherlands) at 120 kV. For confocal laser scanning microscopy (CLSM) observation of chlorophyll, immature spikes were dissected and embedded in 8% agarose placed in a flat-bottomed mold. After concreting, agarose blocks were removed from the mold and cut into 80 μm with a microtome (Leica VT1000 S vibrating blade microtome). Autofluorescence was recorded under the LSM780 CLSM (Carl Zeiss MicroImaging) with a 640-nm laser line and 650- to 720-nm emission. For measuring the vascular area, transverse sections through all rachis of a barley spike were recorded by CLSM with autofluorescence of a 405-nm laser line and 406- to 715-nm emission. The surface area of rachis internodes and vasculature was measured with the open source Fiji software (*67*). The cross-sectional surface area of individual veins was determined as the area surrounded by a bundle sheath. The distinction between central, lateral, and peripheral bundles followed (*68*). Because of their aberrant distribution and different functions in metabolite transportation (*68*, *69*), peripheral bundles were not included in the present investigation, which thus involves lateral and central bundles only.

### Physiological analyses

Sample collections for chlorophyll, phytohormone, and carbohydrate measurements were essentially the same. Briefly, developing spikes were manually dissected and divided into apical, central, and basal sections in a 2-ml Eppendorf tube prefilled with two stainless steel beads. Samples were frozen immediately in liquid nitrogen and stored at −80°C until used. For each biological replication, ~15 (W4.5), ~10 (W5.5), and 5 (W7) were pooled. Sample collection was done between 11:00 a.m. and 1:00 p.m. of a day.

Chlorophyll concentration was determined according to (*70*). After grinding the samples into fine power, samples were added with 1.8 ml of *N*,*N*-dimethylformamide under the fume hood, vortexed, and kept in the dark at 4°C for ~48 hours. Supernatants were then taken after centrifugation at 14,000*g* for 10 min. Then, the absorbance of each sample was read at 647 and 664 nm using a spectrophotometer. Chlorophyll content was calculated by the following equation: chlorophyll a = (12 × *A*^664^ − 3.11 × *A*^647^) × 1.8 ÷ 20; chlorophyll b = (20.78 × *A*^647^ − 4.88 × *A*^664^) × 1.8 ÷ 20.

For phytohormone measurements, samples were placed in precooled (−80°C) TissueLyser II (QIAGEN, USA) racks and homogenized at a frequency of 30 Hz for 1.5 min. The fresh weights of the samples were measured using a precision balance. Samples were weighed into 2-ml safe lock tubes (Eppendorf AG, Germany). Before extraction, two 3-mm ceria-stabilized zirconium oxide beads were placed into each tube. For phytohormone extraction, 1 ml of ice-cold 50% aqueous (v/v) Acetonitrile (ACN) (Th. Geyer GmbH & Co. KG, Germany) containing the internal standards (OlChemim s.r.o., Czech Republic) was added to each tube. Samples were homogenized in a MM 301 vibration mill (Retsch GmbH, Germany) operating at a frequency of 27 Hz for 5 min and afterward sonicated for 3 min at 4°C using a Sonorex ultrasonic bath (BANDELIN electronic GmbH, Germany). Samples were subsequently extracted using a Reax 32 overhead shaker (Heidolph Instruments GmbH, Germany) for at least 30 min. The supernatant was transferred to clean Eppendorf tubes after 10 min of centrifugation at 14,000 rpm and 4°C (CT 15 RE centrifuge, Himac, Japan). All samples were purified using Oasis PRIME HLB RP (1 cm^3^/30 mg) polymer-based Solid-Phase Extraction (SPE) cartridges (Waters, USA). After loading the supernatant, the flow-through fraction was collected in a clean tube. The cartridge was then eluted with 1 ml of 30% (v/v) ACN, and the elute was collected in the same tube as the flow-through fraction. After this single-step SPE, the samples were evaporated to dryness at 40°C in an RVC 2-33 IR vacuum concentrator (Martin Christ GmbH, Germany) and stored at −20°C until analysis. For ultrahigh-performance liquid chromatography (UHPLC)–electrospray ionization (ESI)–tandem mass spectrometry (MS/MS) analysis, the samples were dissolved in 50 μl of 30% ACN (v/v) and transferred to insert-equipped vials. Ten microliters of purified extracts was injected into an Acquity Ultraperformance LC system coupled with a Xevo TQ mass spectrometer (Waters, USA). All targeted phytohormones but gibberellins were separated on an Acquity UPLC BEH C18 1.7 μm, 2.1 × 100 mm column coupled to a VanGuard precolumn BEH C18 1.7 μm, 2.1 × 5 mm. The column temperature was set to 40°C. The autosampler temperature was set to 4°C. The mobile UPLC phase consisted of a gradient of methanol (MeOH; Th. Geyer GmbH & Co. KG, Germany) with 0.1% (v/v) formic acid (Biosolve Chimie, France) (A) and 0.1% (v/v) aqueous formic acid (B), flowing at 0.4 ml/min. A 10-point external calibration curve was used for quantification. MassLynx software (version 4.1; Waters) was used to control the instrument and data acquisition. MS data were processed by using TargetLynx V4.1 SCN 904. Gibberellins’ baseline separation was achieved on a reversed-phase Acquity UPLC HSS T3 column (100 Å, 2.1 × 150 mm, 1.8 μm; Waters) using a gradient elution of A [water and 0.1% formic acid (FA)] and B (MeOH and 0.1% FA) as follows: 0 to 0.3 min, 10% B; 0.3 to 0.7 min, 10 to 30% B; 0.7 to 2 min, 30 to 50% B; 2 to 4 min, 50 to 60% B; 4 to 8 min, 60 to 80% B; 8 to 9.5 min, 80 to 99% B; 9.5 to 10.4 min, 99% B. A guard column (130 Å, 2.1 × 5 mm, 1.8 μm; Waters) was also used to preserve the integrity of the column. The column temperature was set at 45°C, and the flow rate was set at 0.3 ml/min. The injection volume was 5 μl. The UHPLC system was coupled to Q Exactive Plus mass spectrometer (San Jose, CA, USA) equipped with a HESI source operating in negative ion mode. Source values were set as follows: spray voltage, 2.5 kV; capillary temperature, 255°C; S-lens RF level, 40; aux gas heater temperature, 320°C; sheath gas flow rate, 47; aux gas flow rate, 11. For spectra acquisition, a full-MS/dd-MS^2^ experiment was performed. Resolution in full scan was set as 70,000. For MS/MS experiments, a resolution of 17,500 and a normalized collision energy of 40 were used. MS data were acquired and processed by Trace Finder Software (v.4.1, Thermo Fisher Scientific, San Jose, CA, USA). The 12-point curve was prepared from standard mix solutions in the range of 0.5 to 1000 nM. The peak area on the extracted ion chromatogram of the deprotonated molecule ion [M-H] was measured to generate the calibration curve. A least-square linear regression was used to fit the linearity curve best. The identification of compounds found in extracts was based on comparing its retention time, high-resolution mass/charge ratio spectrum, and isotope pattern with standards. In addition, generated MS^2^ spectra were searched into a custom spectral library to confirm compound identification.

For carbohydrate measurements such as starch and soluble sugars, samples were measured enzymatically as described in (*71*) using methanolic extracts of the samples. Raw data of chlorophyll, phytohormone, and carbohydrate measurements are given in table S7.

### RNA sequencing and DYG identification

Tissue collection and RNA extraction were mentioned above. Library construction and sequencing were done at Novogene (UK) Company Limited. Briefly, RNA integrity and quantitation were determined with the Agilent 2100 Bioanalyzer. Total mRNA was enriched with oligo(dT) beads and randomly fragmented with fragmentation buffer. A stranded specific library was then prepared for each sample and sequenced with a NovaSeq 6000 PE150 platform, yielding about 50 million high-quality reads per sample. To quantify gene transcripts, RNA sequencing reads were first trimmed for TruSeq3-PE adaptors with Trimmomatic v.0.39 (*72*) using a maximum of two seed mismatches, a palindrome clip threshold of 30, and a simple clip threshold of 10, and reads shorter than 36 bp were removed. We used Morex genome annotation V2 as a reference to estimate read abundance with Kallisto software (*73*) by using strand-specific mode with the first read reversed (--rf-stranded) parameter. Only genes with read counts of ≥10 in at least three samples were retained for differentially expressed gene (DEG) analysis under DESeq2 (*74*). A pairwise comparison was used to determine DEG, i.e., by considering variances that were contributed by (i) genotypic effect (BW versus *tst2.b*, both in spike sections and anther), (ii) developmental effect (W4.5 versus W5.5, W4.5 versus W7, and W5.5 versus W7 for spike sections and W8 versus W9 for anther), and (iii) positional effect (apical versus central, apical versus basal, and central versus basal for spike sections only). A gene with |log_2_ fold change (FC)| ≥ 1 or |log_2_ FC| ≥ 0.5 (W4.5 comparison between BW and *tst2.b*) in expression and a Benjamini-Hochberg FDR-adjusted *P* value of <0.05 was considered as a DEG. This in total identified 14,216 DYGs for spike sections (table S9) and 10,564 for anther (table S10). For the spike atlas dataset, read abundances were reestimated with Morex genome annotation V2 using Kallisto. After removing lowly expressed genes (same as above), averaged Transcripts Per Kilobase Million (TPMs) from four biological replicates were *Z*-scored, and genes with a coefficient of variation of ≥0.4 over the 12 samples (W2, W2.25, W2.5, W3, W3.5, and W4.5 in two tissue types) were considered as DYGs (table S8).

### Clustering, cis-motif enrichment, GO enrichment analyses, and gene co-regulation analysis

Averaged TPMs from the identified DYGs were *Z*-scored and clustered using the K-medoids method with the PAM algorithm implemented in the R package cluster 2.1.3 (*75*). Euclidean distance was used. The number of partitions to be clustered was determined on the basis of gap statistics. Because of its low expression (TPM < 0.4 for all samples), *HvCMF4* was excluded during the initial clustering analysis. However, a subsequent hierarchical clustering based on the expression patterns of the 10 clusters and *HvCMF4* (normalized TPM values) placed *HvCMF4* to C8 cluster, which was also supported by the RT-qPCR results (fig. S9A). Barley cis-regulatory elements (CREs), which usually reside in accessible chromatin regions (ACRs), are prevalently enriched in distal gene regions (i.e., up to 100 kb) (*76*). To predict cis-motifs that are enriched in different clusters, we thus considered the ACR within the two flanking high-confidence (HC) genes of a DYG as probably putative CREs (on average, ~150-kb distance between two HC genes in barley genome). For this, reads from barley leaf ATAC-seq (*76*) were processed and mapped to Morex reference V2, and ACR identification was done according to (*76*). Overlapped ACRs and the DYG flanking regions were extracted with bedtools (https://bedtools.readthedocs.io/en/latest/). HOMER (v.4.10) (http://homer.ucsd.edu/homer/) “findMotifsGenome.pl” function (*77*) was used for cis-motif enrichment analysis with the parameter “-mset plants.” ACRs from genes that were not present in the DYG list but were expressed in spikes (6060 genes) were used as a background (table S12). We used the corresponding *Arabidopsis* homologs for GO term enrichment analysis. For this, the deduced protein sequences of barley HC genes were used to query *Arabidopsis* protein dataset in The Arabidopsis Information Resource (TAIR) 10. Genes with the highest hits of blastp (*e* value < 1 × 10^−5^) were considered as the closest homologs in *Arabidopsis*. GO term enrichment was done and summarized with Metascape (http://metascape.org) (*78*) using default parameters (table S11). Heatmaps of gene expression clusters and pathways (tables S13 and S14) were generated using ComplexHeatmap (*79*). To inspect commonly regulated genes by *HvCMF4* and the clock pathways, we first defined orthogroups between barley and *Arabidopsis* with OrthoFinder 2.4.1 (*80*) with default parameters. Orthogroups that contain both barley genes and *Arabidopsis* genes regulated by CCA1 (*32*), *PIFs* (*33*), and *HY5* (*34*) were considered as putative commonly regulated genes.
