# Supplementary material for: A molecular framework for grain number determination in barley
Source: Sci Adv. 2023 Mar 3;9(9):eadd0324. doi: 10.1126/sciadv.add0324 (PMC9984178; doi:10.1126/sciadv.add0324)
Supplement: Supplementary file 1 — Figs. S1 to S19 Tables S1, S2, S4 to S7, and S15 Legends for tables S3, S8 to S14 [file sciadv.add0324_sm.pdf]

Science Advances
AAAS

Supplementary Materials for

A molecular framework for grain number determination in barley

Yongyu Huang *et al.*

Corresponding author: Yongyu Huang, huang@ipk-gatersleben.de; Thorsten Schnurbusch, thor@ipk-gatersleben.de

Sci. Adv. **9**, eadd0324 (2023)
DOI: 10.1126/sciadv.add0324

The PDF file includes:

Figs. S1 to S19
Tables S1, S2, S4 to S7, and S15
Legends for tables S3, S8 to S14

Other Supplementary Material for this manuscript includes the following:

Tables S3, S8 to S14

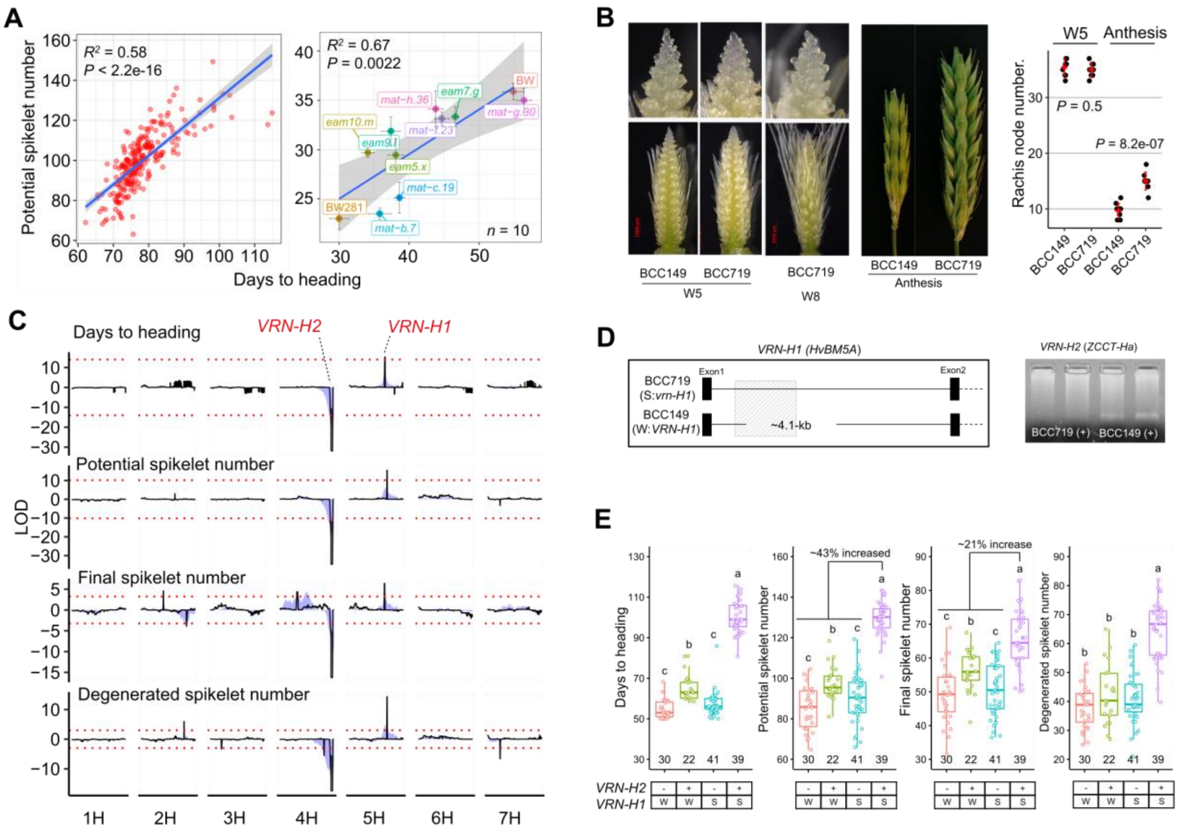

Fig. S1. Barley spikelet initiation is dominated by flowering time genes. (**A**) Days to heading and potential spikelet number relationship in 358 spring barleys and ten independent early heading mutants in the BW background. (**B**) BCC149 and BCC719 spike phenotypes. Spike images from BCC149 and BCC719 at different developmental stages are shown. Note that the collapse of inflorescence meristem can be observed at ~W5 in BCC149 relative to ~W8 in BCC719. Scale bars: 1 mm for developing spikes and 1 cm for the mature spike. A quantitative comparison for rachis node number at maximum yield potential stage (~W5) or anthesis stage is shown in the right panel. Values are shown as mean ± S.E.M. N = 8 – 10. *P* values defined by two-tailed Student's *t*-tests are shown. (**C**) QTL mapping for days to heading and spike morphologies in the BCC149×BCC719 DH population. Colored shades and black lines indicate LOD scores based on single-marker analysis and composite interval mapping methods, respectively. Red dashed horizontal lines depict the suggestive thresholds based on 1,000 permutations. LOD values above or below 0 represent alleles with positive additive effects from BCC719 or BCC149, respectively. (**D**) Variations of the two flowering time genes (*VRN-H2* and *VRN-H1*) in BCC719 and BCC149. The *VRN-H2* locus (flowering repressor, including *ZCCT-Ha* gene) is absent (-) in the BCC719 genome, but present (+) in BCC149 genome; whereas BCC719 and BCC149 contain recessive and dominant alleles at the *VRN-H1* locus (*HvBM5A*, repressor of *VRN-H2*), respectively, which is characterized by a ~4.1-kb insertion/deletion in the first intron of *HvBM5A* gene. W, winter-type (or dominant *VRN-H1*); S, spring-type (or recessive *vrn-H1*). Grey dashed frame indicates the 2.8-kb barley-wheat conserved region (*10*). (**E**) Phenotypic comparison from different

allelic combinations of the *VRN-H1*/*VRN-H2* loci. Letters above boxplot represent statistical significance from ANOVA with Tukey's HSD test, $P < 0.05$.

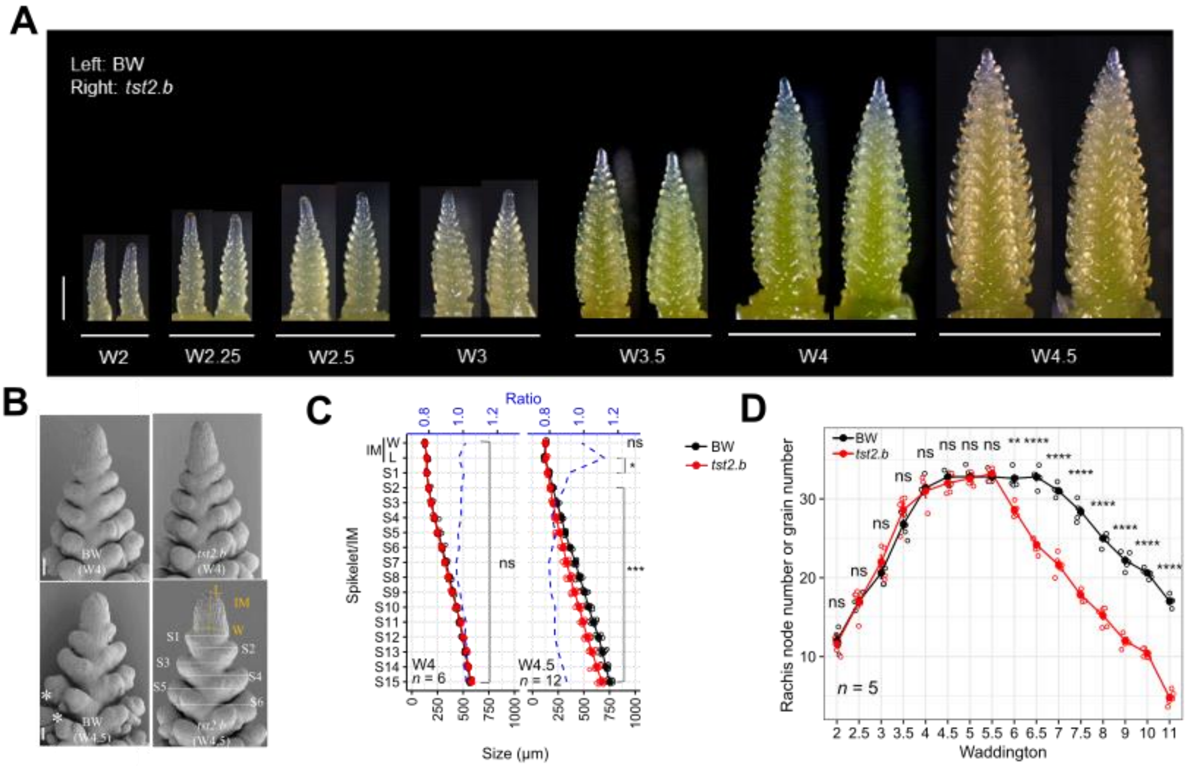

Fig. S2. Spike architecture of BW and *tst2.b*. (**A**) Representative spike images from BW and *tst2.b* before W4.5 stage (W4.5). (**B**, **C**) Quantitative comparisons of spike diameter in BW and *tst2.b* at W4 and W4.5. Representative Scanning Electron Microscope imaging of spikes (B) at W4 and W4.5 from BW and *tst2.b* are shown. Asterisks highlight lemma and glume primordium, which are invisible from the *tst2.b* counterparts. Dashed lines illustrate the size measurements for inflorescence meristem (IM, orange) and spike diameter (S, white). L, length; W, width. Blue dashed line in (C) denotes the mean size ratio of *tst2.b* and BW from each counterpart of the rachis node. (**D**) Quantitative comparison for rachis node number (before W10) or grain number (W11). Scale bars: 500 μm in (A); 50 μm in (B). Significance levels are determined from two-tailed Student's *t*-test. *$P < 0.05$; **$P < 0.01$; ***$P < 0.001$, ****$P < 0.0001$ and ns, not significant.

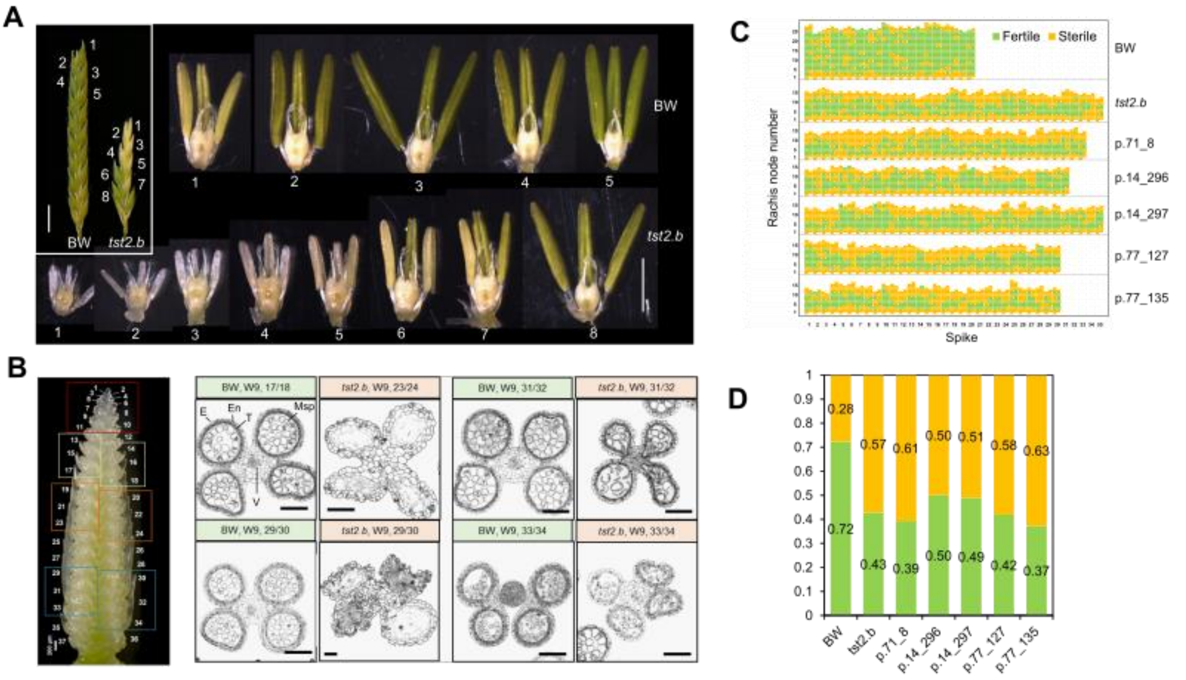

Fig. S3. Pattern of anther fertility in BW and *tst2.b*. (**A**) Representative images of anthers from different positions of a spike at W9. (**B**) Transverse sections showing anther development in BW and *tst2.b* from different positions of a spike at W9. E, epidermis; En, endothecium; T, tapetum; Msp, microspore; V, vasculature. Note that spikelet 1 – 18 in *tst2.b* are prematurely aborted before anthesis; whereas this number is 1 – 11 in BW. Thus spikelet 19 – 24 and 13 – 18 are the topmost ones from a mature spike in *tst2.b* and BW, respectively. (**C**, **D**) Pattern of anther fertility from BW, *tst2.b* and five homozygous recombinant families (see below gene mapping). Main culm spikes at grain filling stage are used for phenotyping. Fertility in (D) was deduced by dividing number of fertile or sterile spikelets by total spikelet number. Scale bars: 1 cm and 2 mm in whole spikes and anthers sections in (A); 200 µm and 50 µm in whole spike and transverse sections in (B).

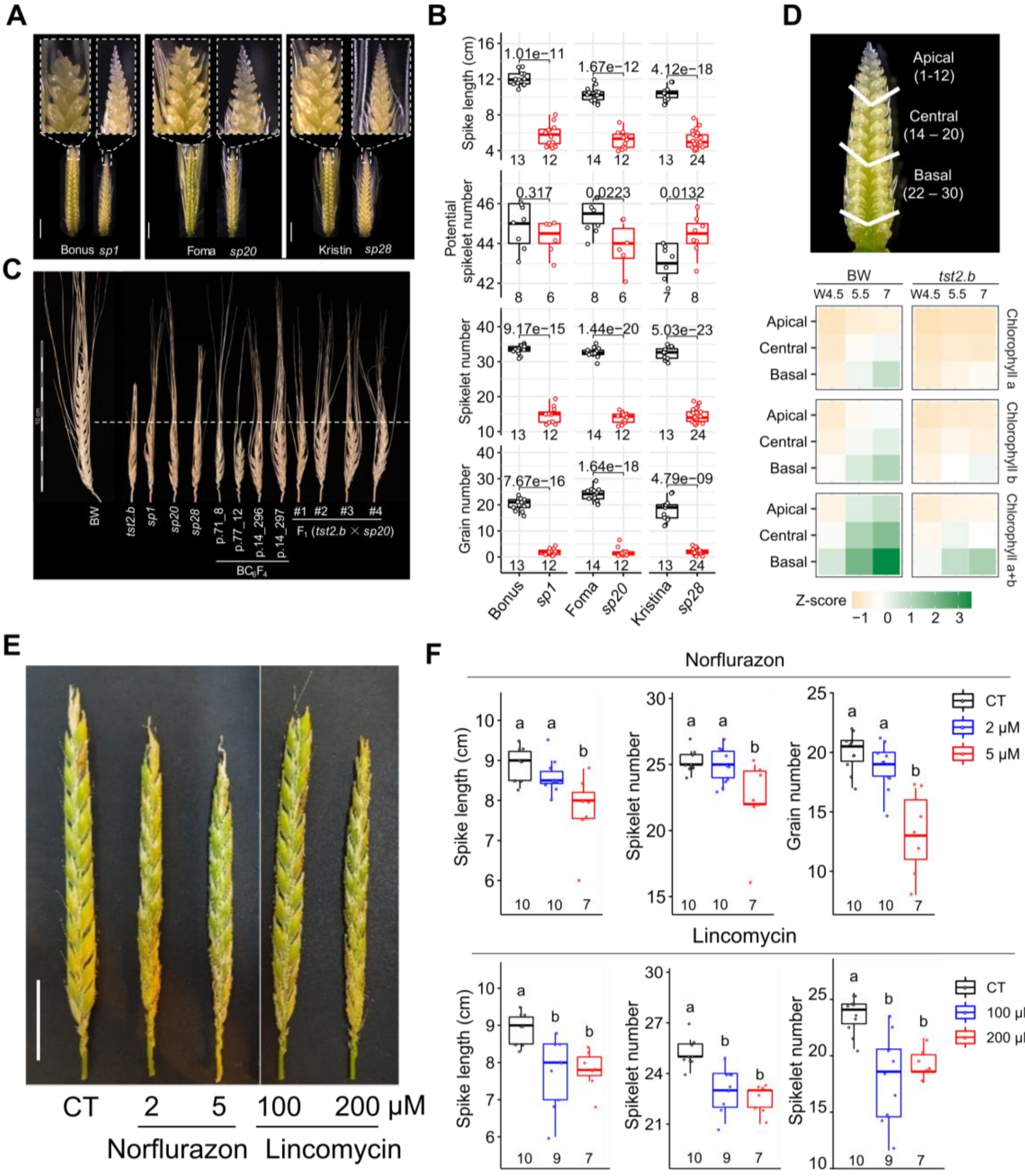

Fig. S4. Short spike mutant phenotypes, allelism test, chlorophyll measurement and chemical treatments. (**A**) Spike images at maximum yield potential stage. Inserts highlight premature tip degeneration in the mutants compared with the WT. (**B**) Quantitative comparison for spike phenotypes. Significance levels are determined from two-tailed Student's *t*-test. (**C**) Mature spikes from the *short spike* mutants, BW, *tst2.b* and F_1 of *tst2.b* × *sp20*. *tst2.b* and *sp20* are used as paternal and maternal parents, respectively. Four positive F_1 hybrids did not recover from the short spike phenotypes, suggestive of allelism between *tst2.b* and *sp20*. Dashed line highlights the approximate tip positions from the four F_1 spikes. (**D**) Quantification of spike chlorophyll content in BW and *tst2.b*. Spike sectioning methodology is shown in the upper panel. Relative mean (5 replicates) chlorophyll contents from different spike sections and developmental stages are shown in the heatmap. Source data are given in (table S5). (**E**) Spike images from main culms of barley plants treated with different concentrations of chloroplast biogenesis inhibitors norflurazon and lincomycin, or control (CT). (**F**) Quantitative

comparison of spike phenotypes from plants treated with different chemicals and concentrations. Letters above plots represent statistical significance by ANOVA with Tukey's HSD, $P < 0.05$. Scale bars: 2 mm in (A); 10 cm in (C) and 2 cm in (E).

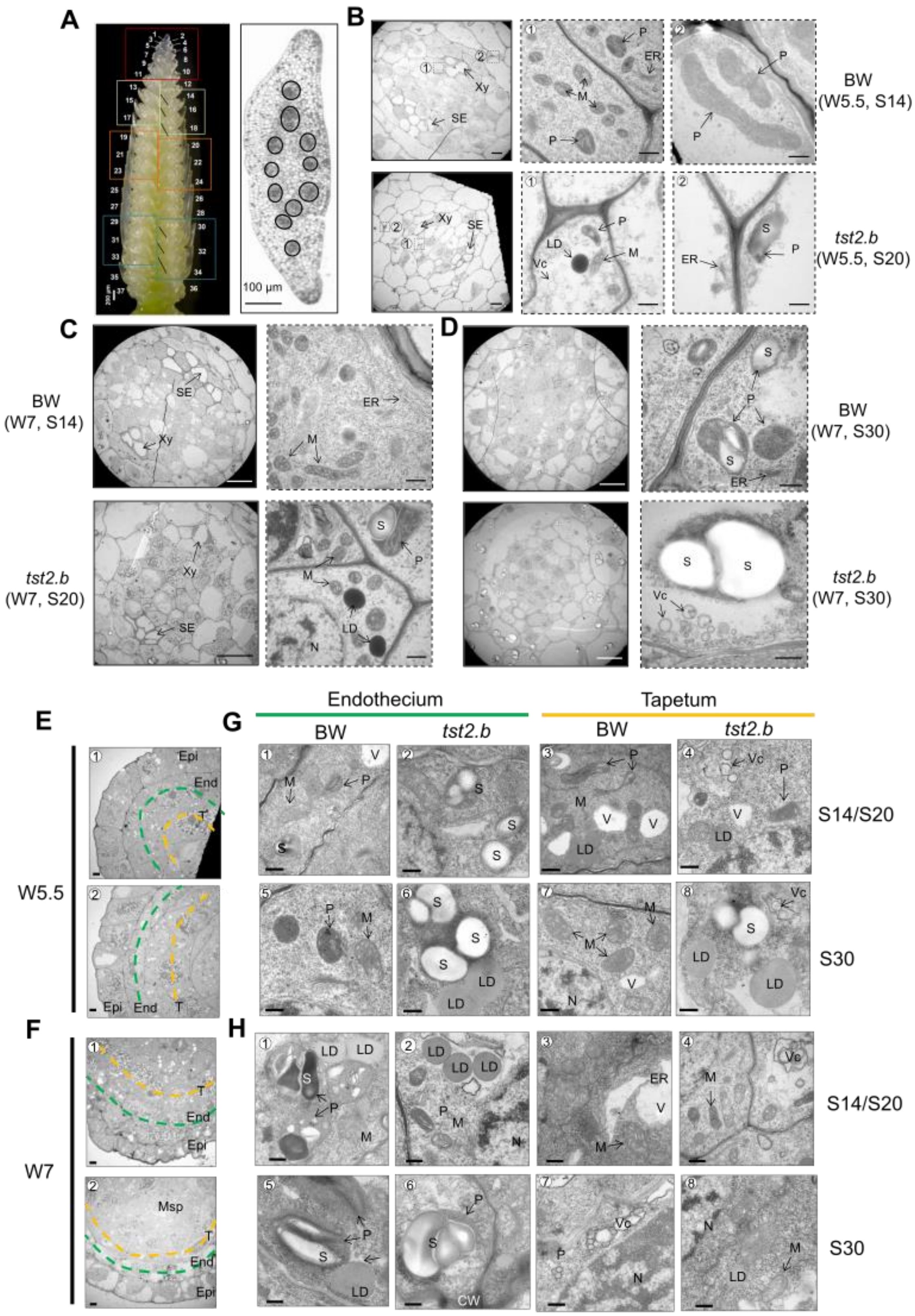

Fig. S5. Ultrastructure of plastids from rachis and anthers revealed by TEM observation. **(A)** Representative image showing the spikelet position for Transmission Electron Microscope (TEM) observation. Note that due to early suppression of development in *tst2.b*, rachis nodes 1 – 18 in *tst2.b* are highly suppressed and unelongated. Therefore, rachis nodes from the orange box (19 – 24) of *tst2.b* are used to compare with those from the lightgreen box (13 – 18) in BW; whereas rachis nodes from

blue box (29 – 34) of both *tst2.b* and BW are used for comparison. Black lines show the approximate position for sectioning. Black circles mark vasculatures from a representative section. (**B** – **H**) Representative TEM images showing the ultrastructure of plastids at W5.5 (B) and W7 (C and D) stages from rachis or anthers (E – H) at different spikelets indicated in (A). Colored dashed lines in (E and F) indicate the cell layers of endothecium and tapetum. Images in (G and H) are arranged according to the top and right annotation. SE, sieve element; Xy, xylem; P, plastid; M, mitochondria; ER, endopoplasmatic reticulum; S, starch; LD, lipid droplet; Vc, vesicle. Scale bars: 200 µm and 100 µm in (A) left and right panels; 10 µm in (B – D) left panels; 500 nm in (B) middle and right panels, and in (C and D) right panels; 2 µm in (E and F); 250 nm in (G and H).

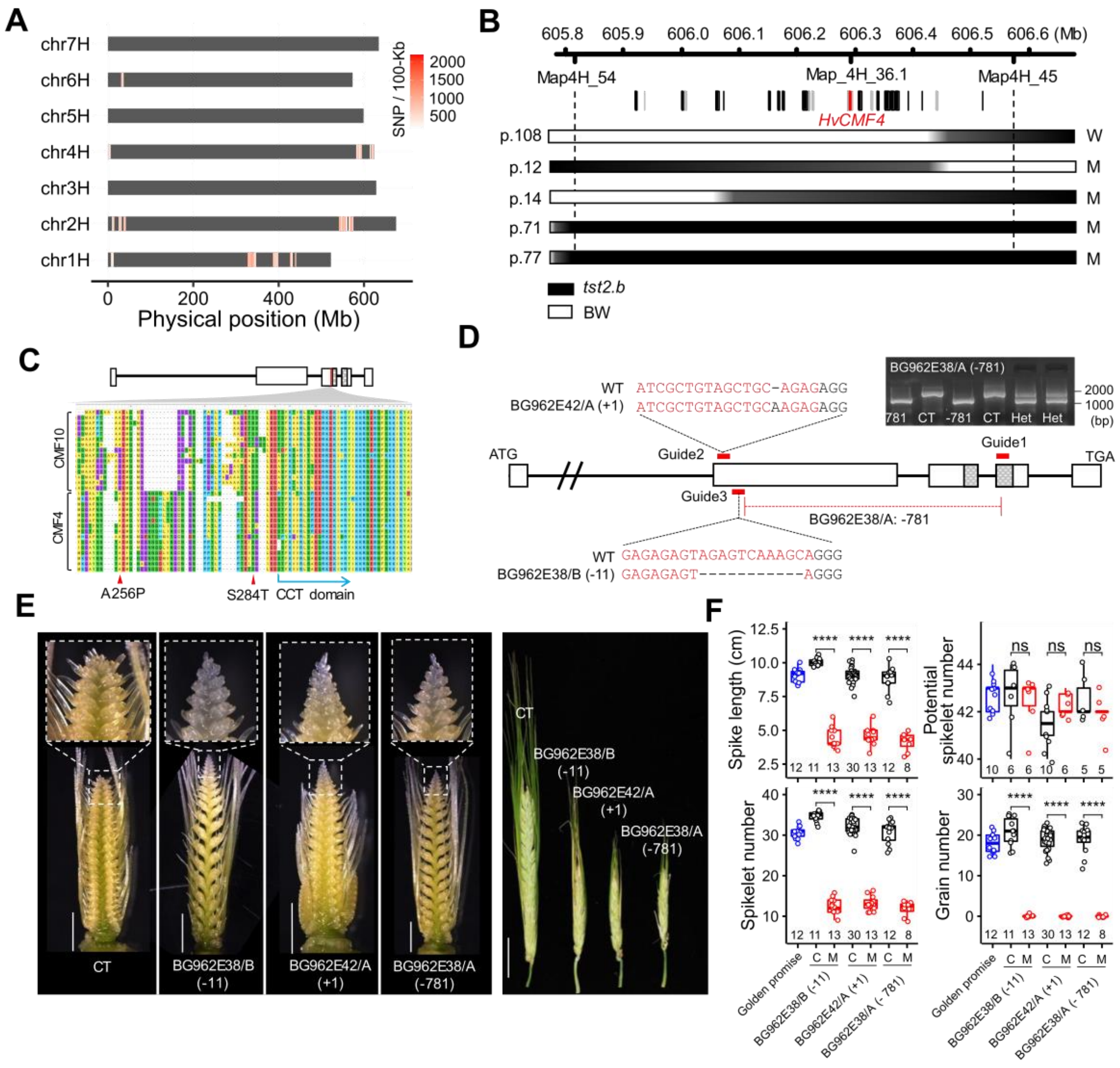

Fig. S6. Map-based cloning and gene knock-out. (**A**) Chromosomal introgressions from Ackermann's Donaria in the *tst2.b* mutant background revealed by whole genome sequencing. (**B**) Graphical genotypes and phenotypes of five recombinant families. Three key recombinants (p.108, p.12 and p.14) delimited the final mapping interval to a ~750-kb region. Detail genotypic and phenotypic data are listed in (table S4a). (**C**) Protein sequence alignment surrounding two conserved amino acids substitutions between BCC149 and BCC719. CMF10 is paralogous to CMF4 (fig. S8) and is used as a control. Sequences from 15 species are used. (**D**) Schematic representative of the positions of three gRNA sequences (Guide1 – 3) and different type of mutations. White boxes are gene exons, black solid lines are gene introns. PCR genotyping for detection of a homozygous 781-bp deletion presents in a M_2 plants of BG962E38/A line is shown. (**E**) Representative spike images at maximum yield potential stage or heading stage from three knock-out mutants and control plant (CT). Note that azygous with two copies of wild-type allele from the M_2 plants are used as CT. Inserts highlight prematurely spikelet abortion in the mutants compared with the CT. Scale bars in (C): 200 µm for developing spikes and 2 cm for spikes at heading stage. (**F**) Quantitative comparison for spike phenotypes from the three knock-out mutants and the corresponding wild type segregant (CT) in M_2 plants. Wild type Golden promise (GP) is used as an additional control. Significance levels are determined from two-tailed Student's *t*-test.

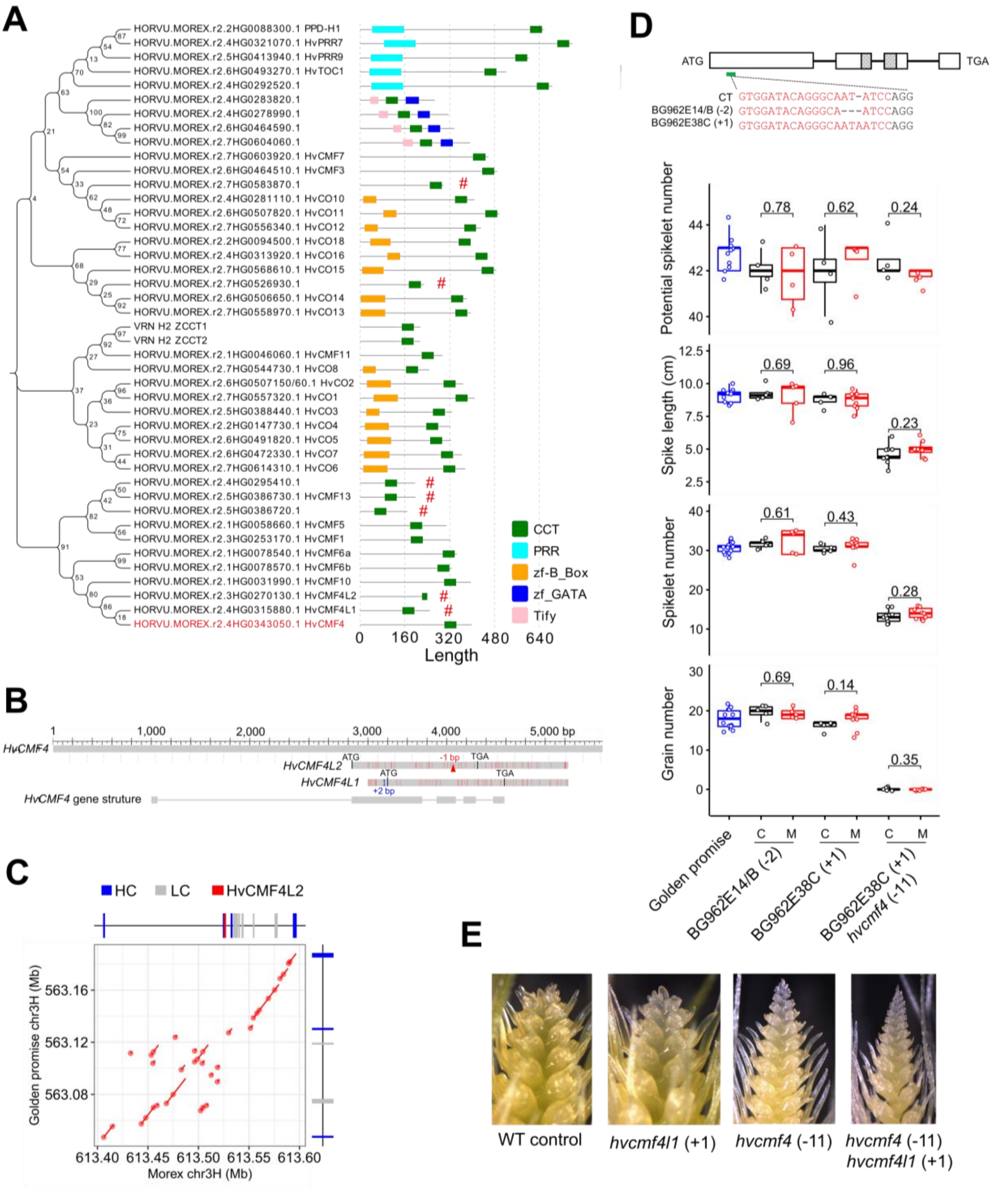

Fig. S7. Reannotation of CCT proteins in the barley genome and *HvCMF4* partial gene duplications. (**A**) CCT genes from the barley genome. Six newly identified putative CMF proteins are marked with red '#'. Two CCT proteins (ZCCT-Ha and ZCCT-Hb) from the *VRN-H2* locus of wild barley FT11 are included for phylogenetic analysis. HvCMF4 is highlighted in red. (**B**) Sequence alignment of the genomic sequences from *HvCMF4*, *HvCMF4L1* and *HvCMF4L2*. Red lines indicate SNPs polymorphism, blue line indicates a 2-bp insertion. Both genes likely encode a truncated version of the HvCMF4 protein: in *HvCMF4L1*, a 2-bp insertion in exon2 could result in a shift of start codon towards the middle part of exon2; in *HvCMF4L2*, a 1-bp deletion in the CCT coding region creates frame shift mutation. (**C**) A dotplot of Morex and Golden Promise genomic sequences from the

HvCMF4L2 region showing a chromosomal rearrangement/deletion including *HvCMF4L2*. HC and LC, high- and low- confidence genes. Note that *HvCMF4L2* is not present in 6 out of the 20 barley reference genomes using BLAST analyses, including Golden promise. (**D, E**) *HvCMF4L1* gene knock out. The position of the gRNA sequence and two type of mutations within *HvCMF4L1* at T_1 generation are shown in (D) top panel. Quantitative comparison for spike phenotypes from *hvcmf4l1* single mutants or *hvcmf4l1*/*hvcmf4* double mutants and the wild type segregants (CT) in T_1 generation is shown in (D) bottom panel. Wild type Golden promise (GP) is used as an additional control. Significance levels are determined from two-tailed Student's *t*-test. Representative apical spike images from the mutants and WT control at W5.5 are shown in (E).

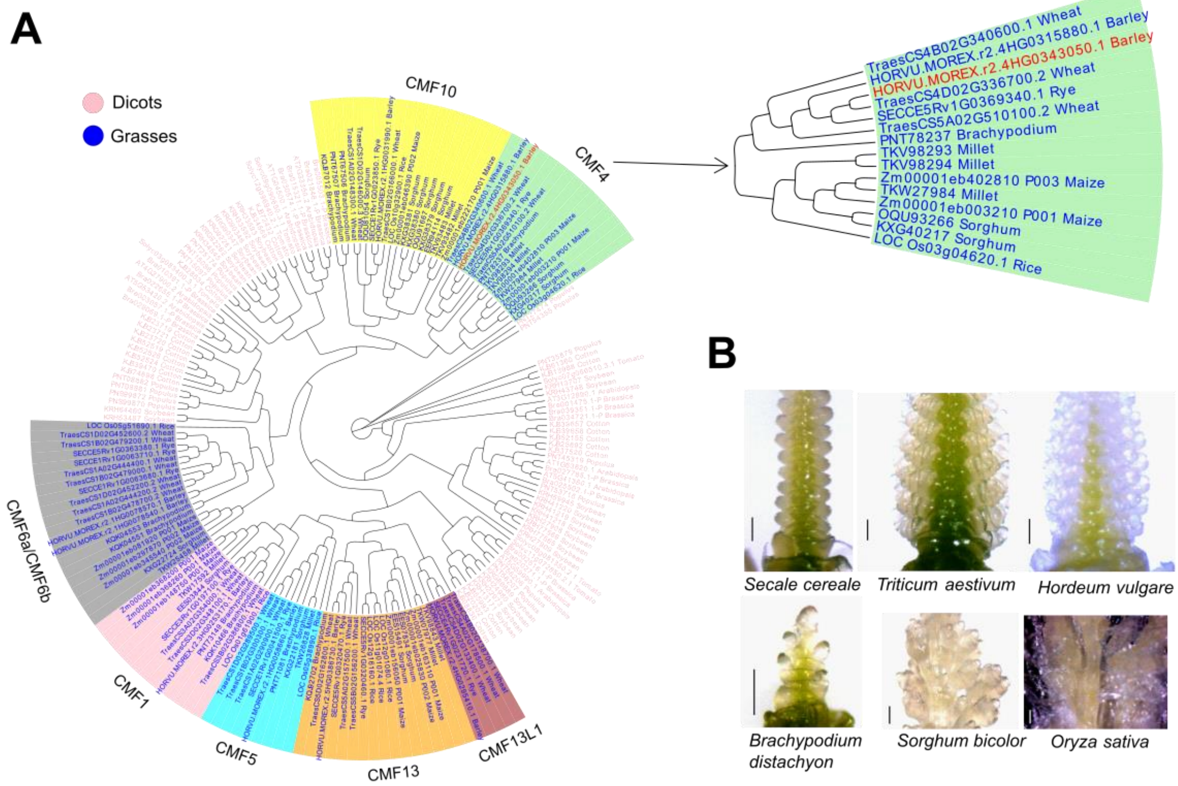

Fig. S8. CMF4-based phylogenic reconstruction in 14 plant species. (**A**) A maximum-likelihood (ML) phylogenetic tree showing the evolutionary relationship of CMF4 proteins among 14 plant species, including six Eudicots and eight grasses (Monocots). Grass specific CMF4 clade is highlighted with light green background, in which barley CMF4 is marked with red color. (**B**) Inflorescences of six grass species at spikelet initiation or differentiation stages, including rye Lo7 (*Secale cereale*), wheat (*Triticum aestivum*), barley Morex (*Hordeum vulgare*), Brachypodium Bd21 (*Brachypodium distachyon*), sorghum BTx623 (*Sorghum bicolor*) and rice Nipponbare (*Oryza sativa*). Scale bars: 200 µm.

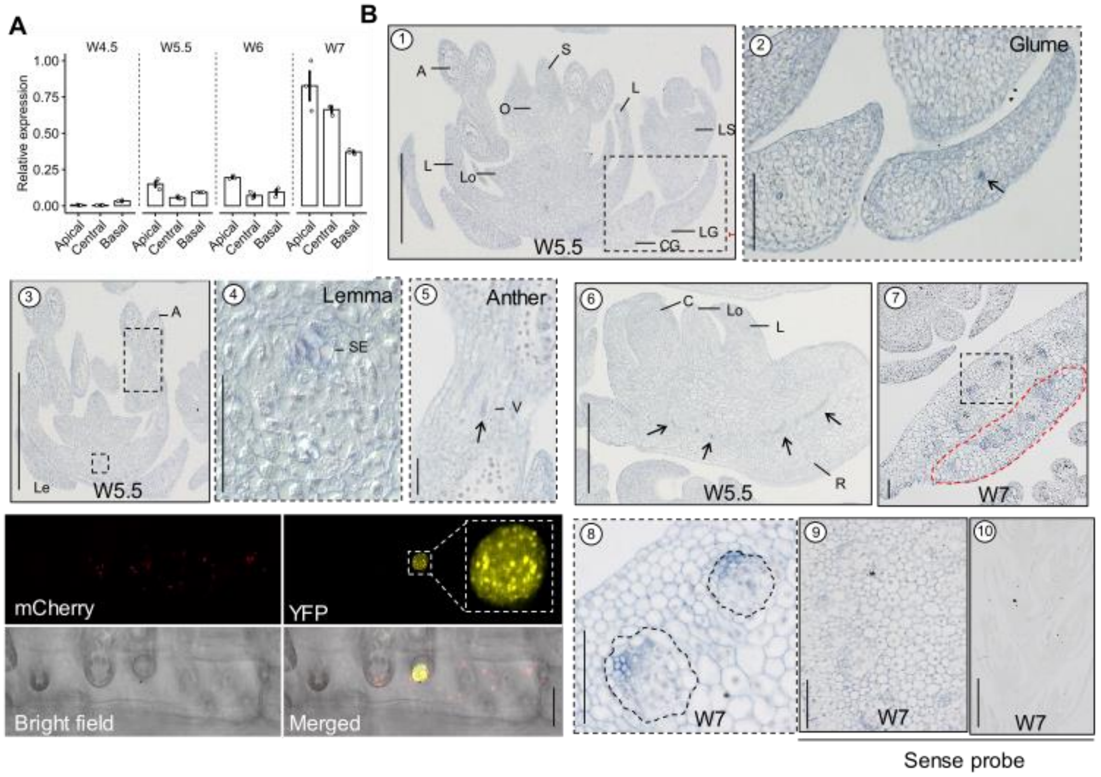

Fig. S9. mRNA expression profile of *HvCMF4* and protein localization. (**A**) Relative expression of *HvCMF4* in spike tissues at different sections and stages. Actin was used for normalization. Data are shown as mean ± s.e.m.; $n = 3$ biological replicates; each replicate was from the pool of 5-20 individuals. (**B**) *In situ* hybridization of *HvCMF4* at different spike sections. Images with dashed frame are zoom in views. (B1 – B6) show different longitudinal sections at parasagittal plane from the middle part of a spike at W5.5 stage. (B1 and B2): glume, arrow points to the vasculature; (B3 – B5): lemma (B4) or anther (B5), arrow points to the vasculature; (B6): rachis – spikelet junction, arrows point to the vasculature. (B7 and B8) show cross sections at W7 stage. The vasculatures that will extend to next spikelet are marked with red dashed circle. Dashed circles in (B8) mark the vascular cylinders from a rachis. (B9 and B10) show cross sections and longitudinal sections (frontal plane) hybridized with a *HvCMF4* sense probe, which are negative controls. Abbreviations: A, anther; S, style; O, ovule; Lo, lodicule; L, lemma; CG, central glume; LG, lateral glume; LS, lateral spikelet; C, carpel; R, rachis. (**C**) HvCMF4-YFP fusion protein targets to nuclear body in barley leaf epidermal cell. Scale bars: 50 µm in (B2, B4, B5 and B7 – B9); 200 µm in (B1, B3, B6, and B10) and 20 µm in (C).

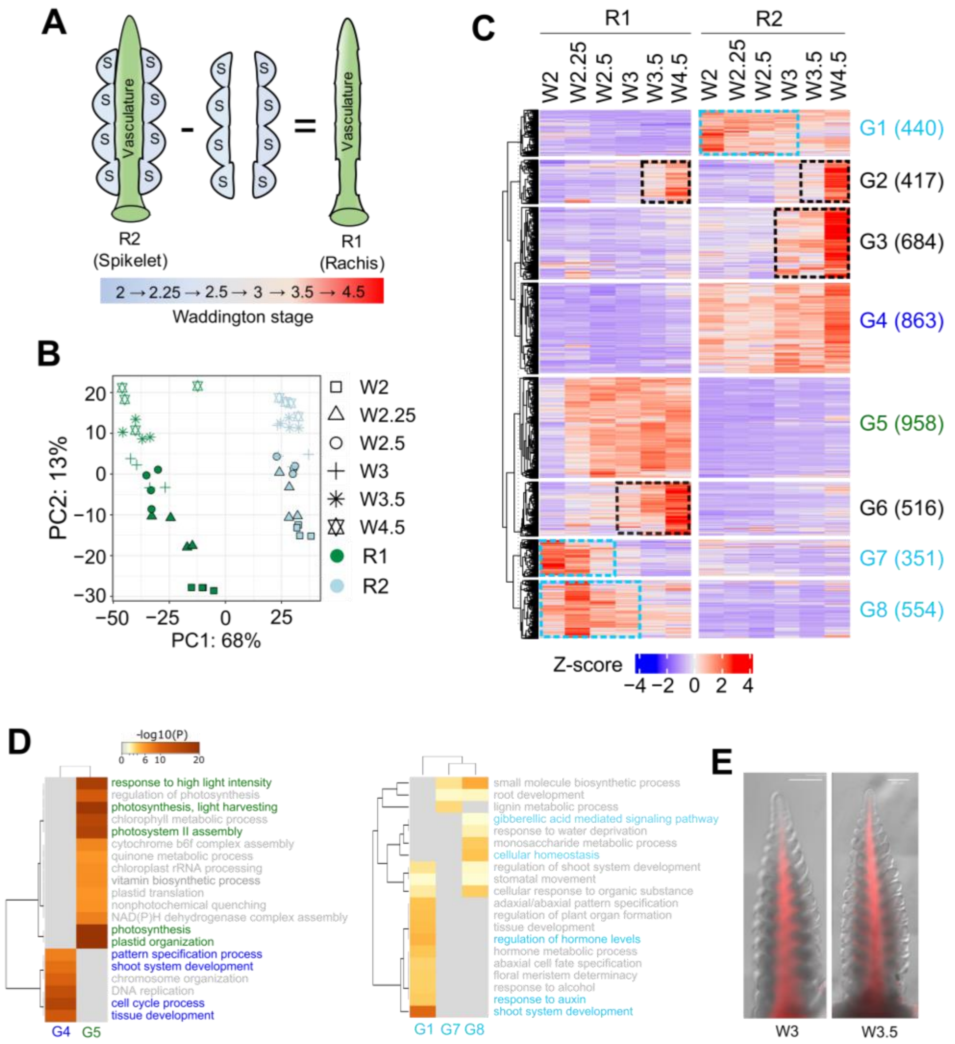

Fig. S10. Transcriptional programs associated with rachis and spikelet development. (**A**) Schematic of the two tissue types (R1 and R2) across six developmental stages (W2 – W4.5). Note that the amount of transcripts associated with R2 tissue (whole spike section) can be disentangled into two sources originating from spikelet (S) and main rachis (mostly vasculature). (**B**) The first two components (PC1 and PC2) from a principle component analysis (PCA). PC1 and PC2 correspond to the two tissue types (PC1) and developmental stage (PC2), respectively. (**C**) Heatmap showing the co-expressed clusters identified from a K-medoids clustering of the DYGs from R1 and R2 across six developmental stages. Gene numbers from each cluster are given. Note that two sequential transcriptional waves intersecting at ~W3 can be observed, with the first wave peaks at ~W2 (blue dashed frames) and the second wave peaks at ~W4.5 (black dashed frames). (**D**) The enriched GO terms of genes from either of the three clusters in the first wave (G1, G7 and G8), or the two relative stable clusters (G4 and G5). Top non-redundant enriched terms are hierarchically clustered and visualized using Metascape. Full GO term lists are given in table S9. (**E**) Chlorophyll autofluorescence at W3 (lemma primordia) and W3.5 (stamen primordia) stages. Scale bars: 200 μm.

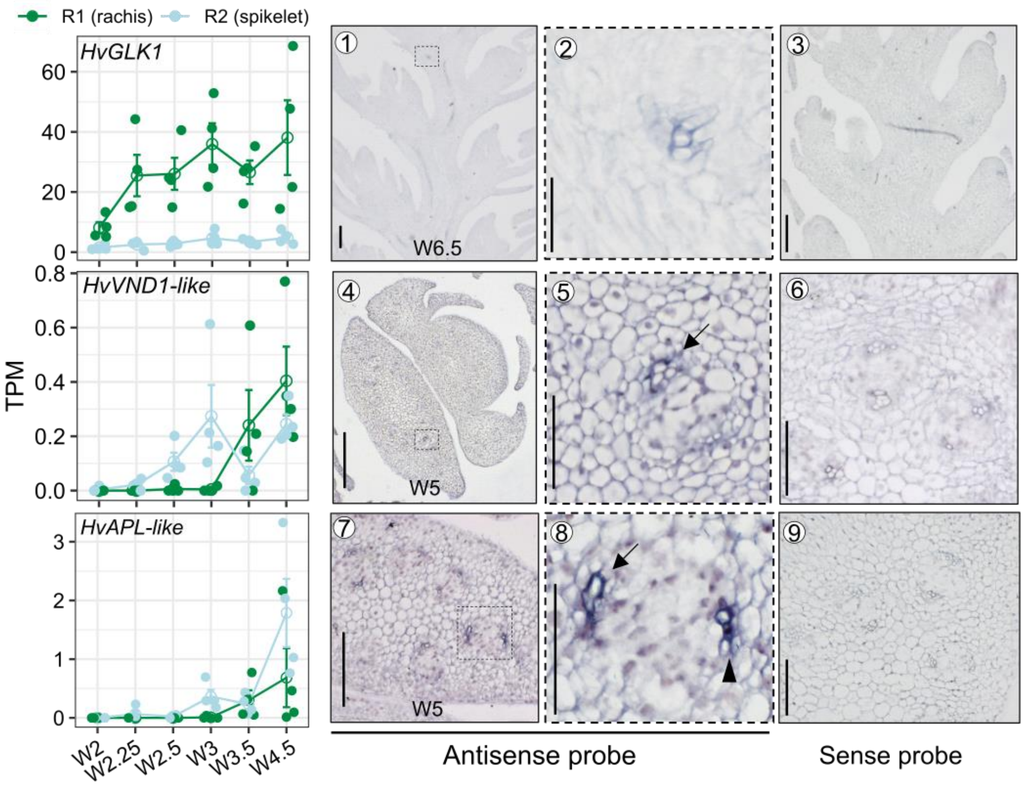

Fig. S11. Patterns of vascular and chloroplast genes expression. *In situ* hybridization of selected transcription factors (TFs, peak expression at ~W4.5) involved in chloroplast development (*HvGLK1*), secondary cell wall formation (*HvVND1-like*, a NAC-domain TF required for xylem vessel formation) and phloem development (*HvAPL-like*, a MYB coiled-coil-type TF required for phloem identity). Protoxylem and protophloem are indicated with arrows and arrowhead, respectively. Values are shown as mean ± s.e.m. Scale bars: 100 µm in (A1, A3, A4 and A7); 20 µm in (A2, A5, A6, A8 and A9).

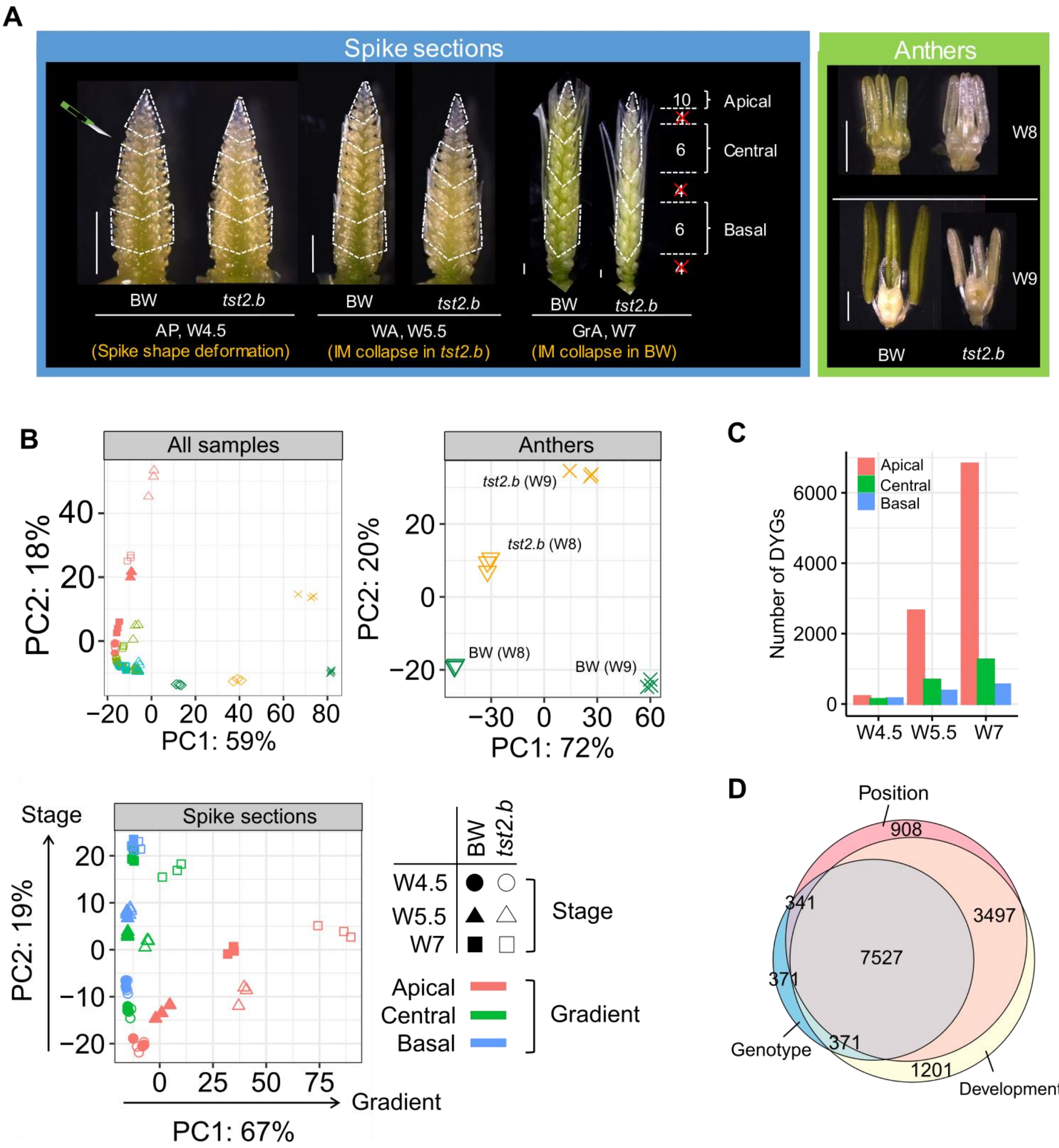

Fig. S12. *HvCMF4* orchestrates transcriptome reprogramming. (**A**) Sampling details in the current transcriptomic study. Spike sections or anthers are hand-dissected with a scalpel. Scale bars: 1 mm. (**B**) Principal component analysis (PCA) of the samples. Note that the first two components account for most of the variances in the current dataset (92% in anthers and 86% in spike sections, respectively). PC1 largely separates the three spike sections (developmental gradient) apart, whereas PC2 accounts for different growh stages. (**C**) Number of dynamically expressed genes (DYGs) affected by the loss of *HvCMF4* (genotypic effect). (**D**) A Venn Diagram depicts the overlap of the 14,216 DYGs perturbed by loss of *HvCMF4* (genotype), developmental gradient (position) and growth stage (stage).

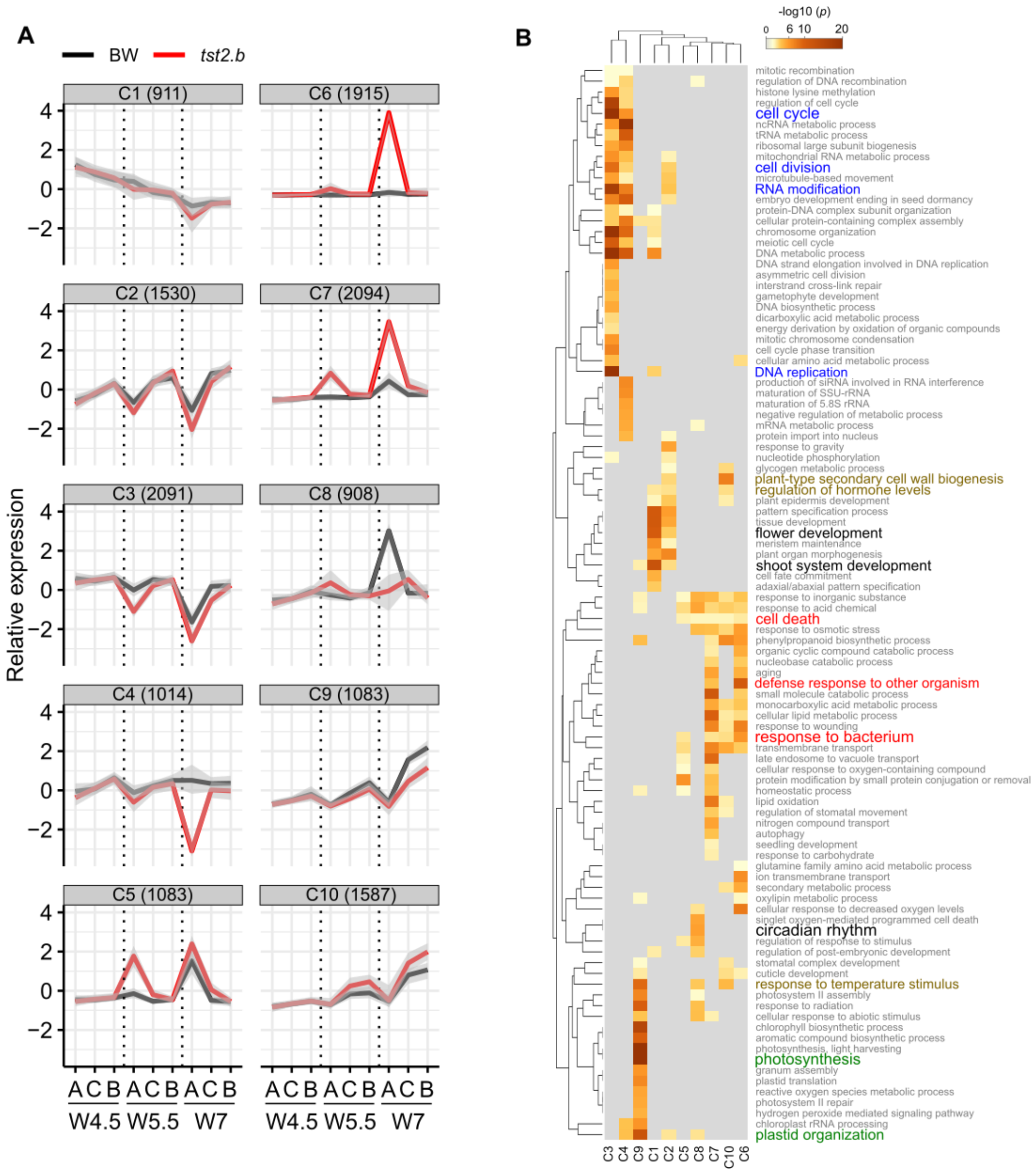

Fig. S13. Gene CO-expression clusters and their enriched biological pathways. (**A**) Trend plot summarizing the expression pattern of each of the ten clusters. "A", "C" and "B" correspond to "Apical", "Central" and "Basal", respectively. (**B**) The enriched GO terms from each of the ten clusters. Top 100 enrichment terms are hierarchically clustered and visualized with Metascape. Selected functional categories are highlighted in bold and colored according to Fig. 4A.

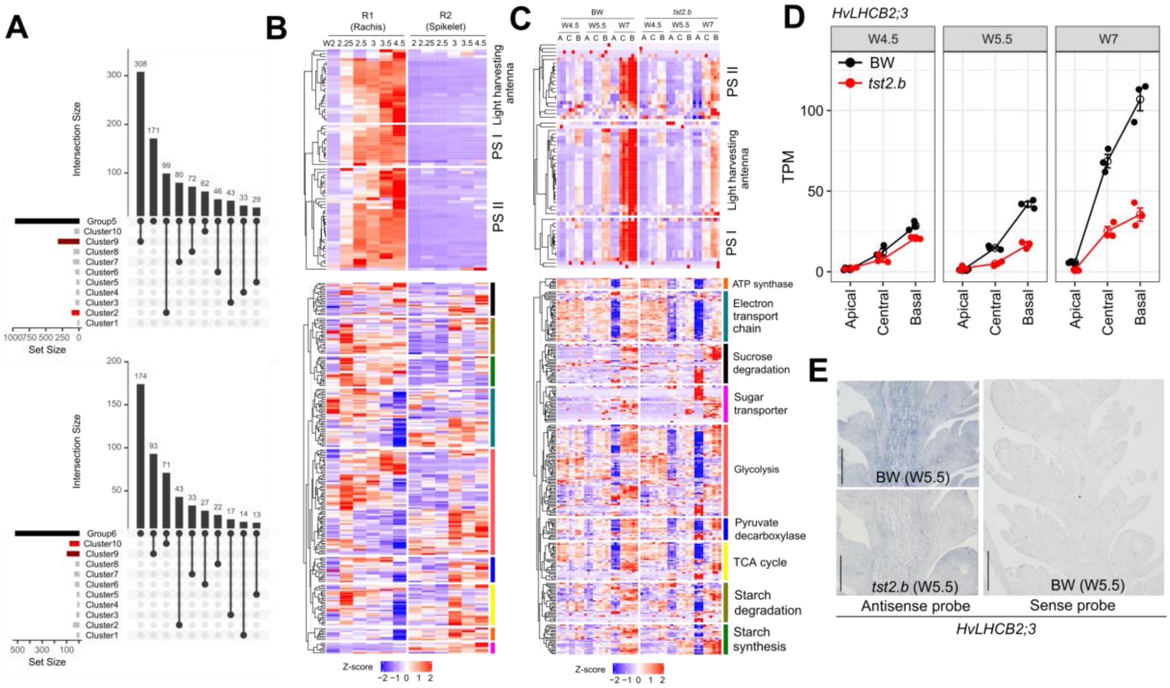

Fig. S14. Expression of genes involved in the assembly of photosystem or energy metabolism. (**A**) UpSet plots showing genes for chloroplast development are mostly from G5 and G6 before awn primordia stage, and C9 after awn primordia stage. The top ten intersections are shown. **(B, C)** Expressions of genes for photosystem assembly (top two) or ATP and starch metabolism (bottom two) before (left) and after (right) awn primordia stage. Whole set of photosystem assembly genes are extracted from map00195 of Kyoto Encyclopedia of Genes and Genomes database (http://www.genome.jp/kegg/); barley energy metabolism genes are downloaded from Plant Metabolic Network database (https://plantcyc.org/). Colored bars from the lower left heatmap are named according to the lower right heatmap with the same colors. PS I and II, photosystem I and II. (**D**, **E**) Expression of an exemplified LHCB gene *HvLHCB2;3*. A trend plot from the spike sections after awn primordia stage or *in situ* hybridization are shown. Values are shown as mean ± s.e.m. Scale bars: 200 µm.

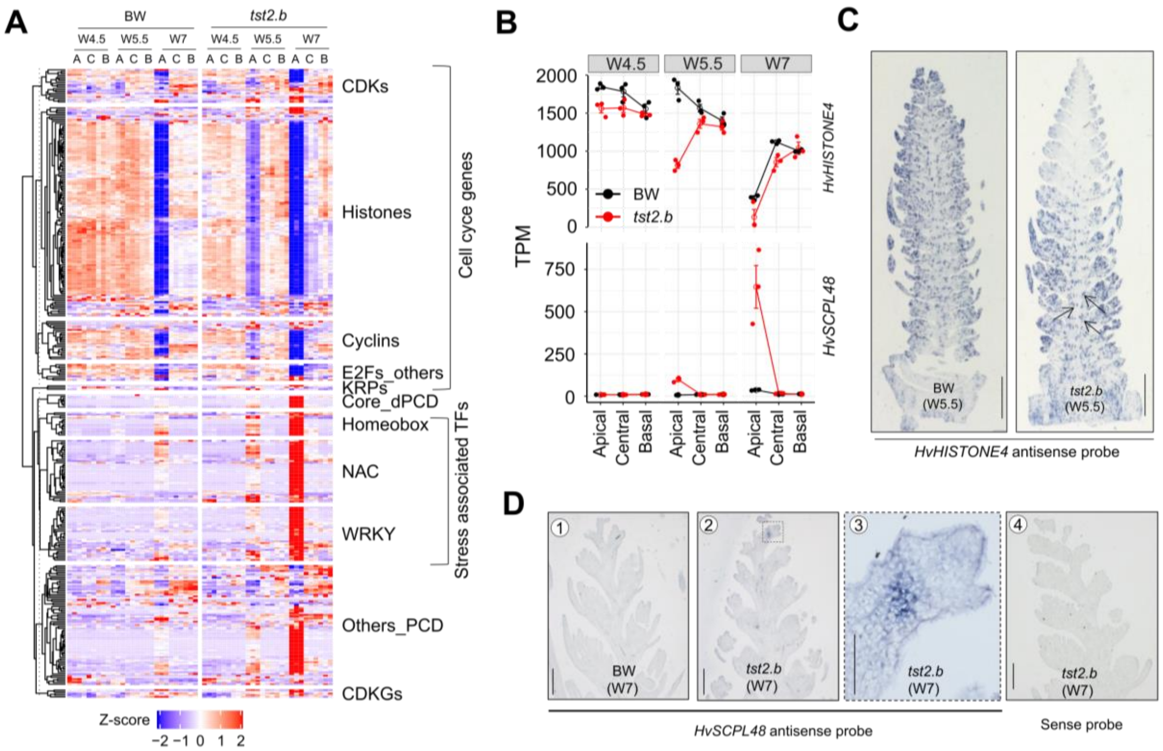

Fig. S15. Expressions of both cell death and cell cycle genes show heterochronic and heterotopic shifts. (**A**) Heatmap showing expression of genes from stress associated TF families (including NAC, WRKY, and homeobox), programmed cell death (PCD, including core set and others) and cell cycle from spike sections after awn primordia stage. (**B** – **D**) Expressions of a cell cycle marker gene (*HvHISTONE4*) and a cell death indicator (*HvSCPL48*). (B) shows a trend plot from the spike sections after awn primordia stage for both genes. Values are shown as mean ± s.e.m. (C and D) show *in situ* hybridization from spike longitudinal sections for *HvHISONE4* (C) at W4.5 stage or *HvSCPL48* (D) at W7 stage. Values are shown as mean ± s.e.m. Scale bars: 500 µm in (C), 200 µm in (D1, D2 and D4); 50 µm in (D3).

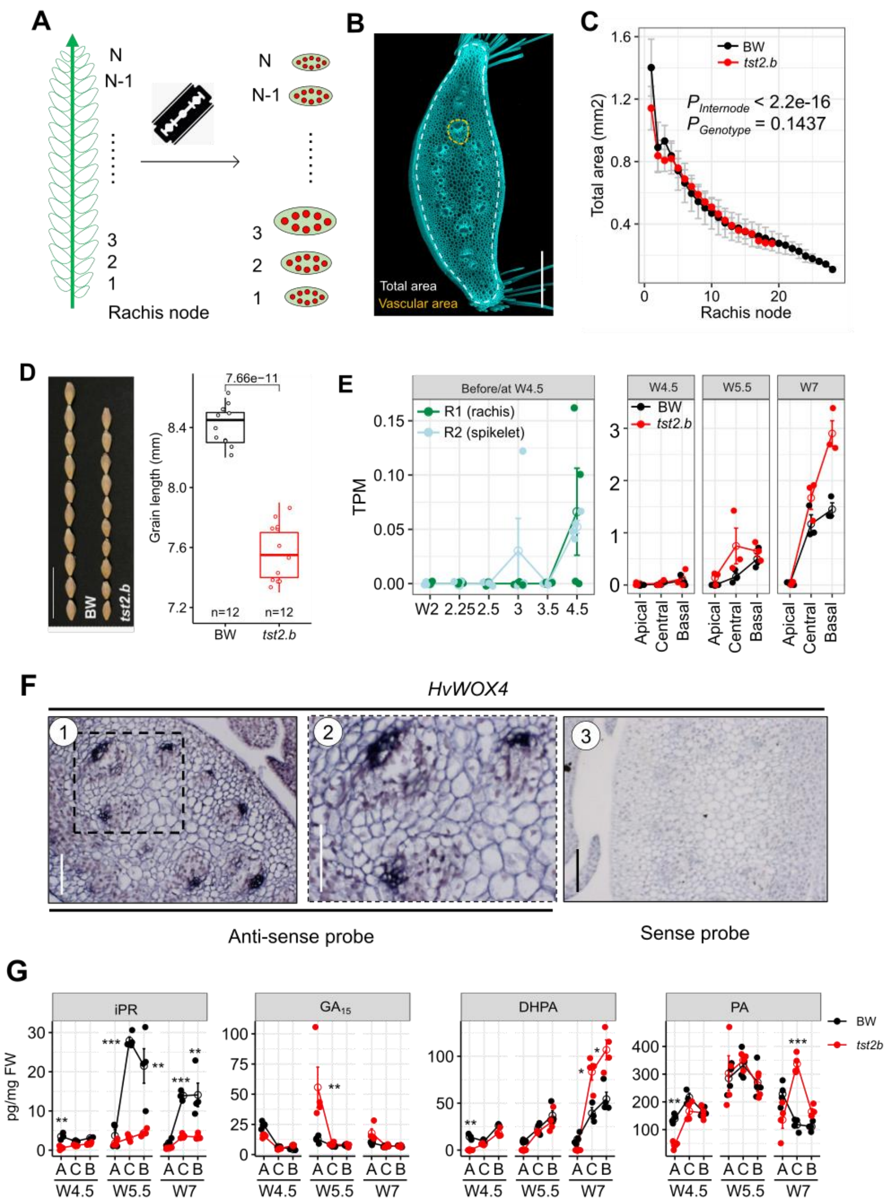

Fig. S16. Comparison of vascular patterning, vascular gene expression and phytohormones in BW and *tst2.b*. (**A**) A schematic illustration of serial transverse sectioning for spike vascular size comparison between BW and *tst2.b* at anthesis stage. Red colors represent vasculatures. (**B**) A representative rachis transverse section image depicts the measurements of tissue areas. (**C**) Total tissue area is not affected by the loss of *HvCMF4*. Data are shown as mean ± SD. P value from internode and genotypic effects are determined with two-way ANOVA. (**D**) Grain size comparison. Significance levels are determined from two-tailed student's *t*-test. (**E**) *HvWOX4* expressions before and after W4.5 stage. Values are shown as mean ± S.E.M.. (**F**) *In situ* hybridization of *HvWOX4* in spike transverse sections at W5.5 stage. Scale bars: 200 µm in (B); 2 cm in (D) and 50 µm in (F). (**G**) Phytohormone profiling from different spike sections across three developmental stages. iPR, N^6-(Δ^2-isopentenyl) adenosine (cytokinin); GA_{15}, gibberellic acid; DHPA and PA, dihydrophaseic acid and phaseic acid (both are ABA catabolites). Data are shown as mean ± S.E.M. *n* = 4 or 5 biological replicates. Significance levels are determined from two-tailed Student's *t*-test. **P* < 0.05; ***P* < 0.01; ****P* < 0.001.

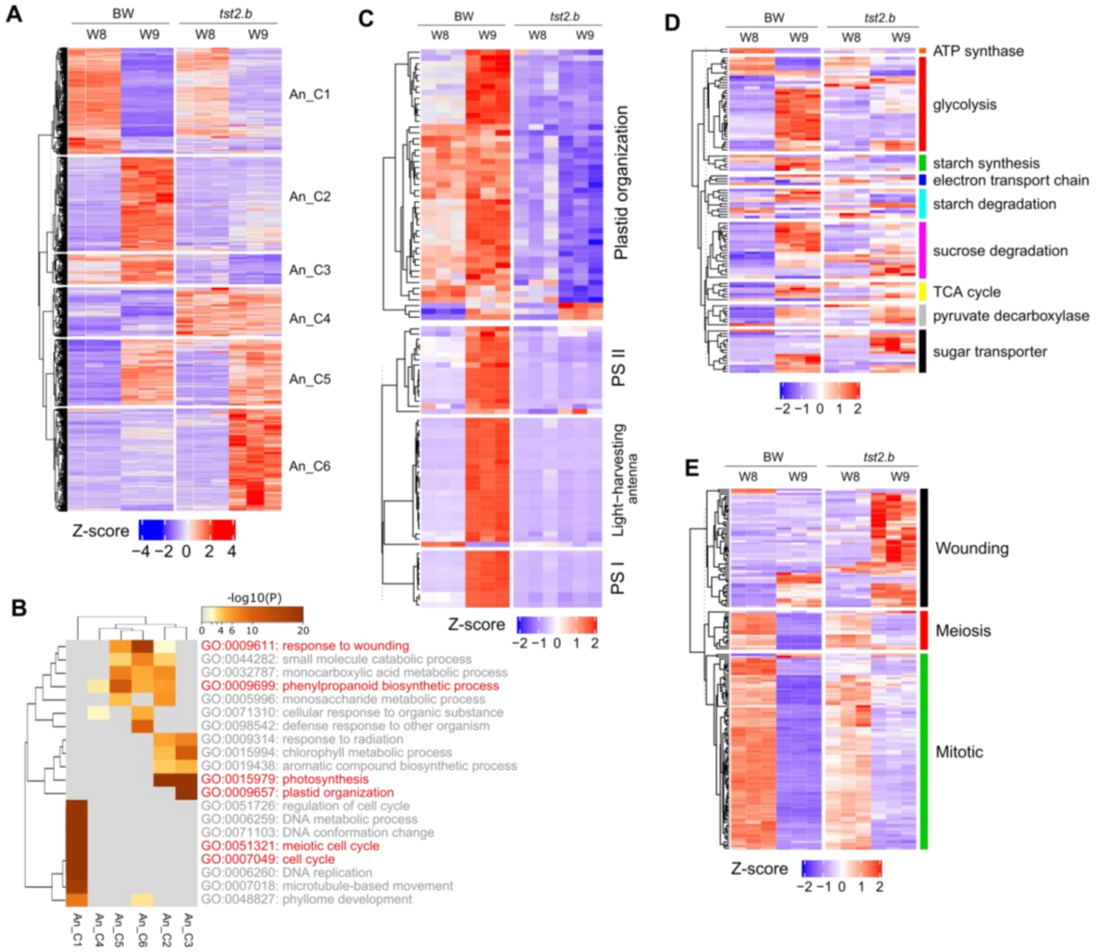

Fig. S17. Co-expressed genes from anther transcriptome of BW and *tst2.b*. (**A**) Heatmap showing the co-expression clusters (An_C1 – An_C6) from a transcriptomic study of anthers at W8 and W9. (**B**) The enriched GO terms from each of the ten clusters. Top non-redundant enrichment terms are hierarchically clustered and visualized with Metascape. Terms related to (Figure 4F) are highlighted i red. Full GO term lists are given in (Table S9). (**C** – **E**) Heatmaps showing *HvCMF4* controls plastid differentiation thereby promoting energy generation to fuel pollen maturation.

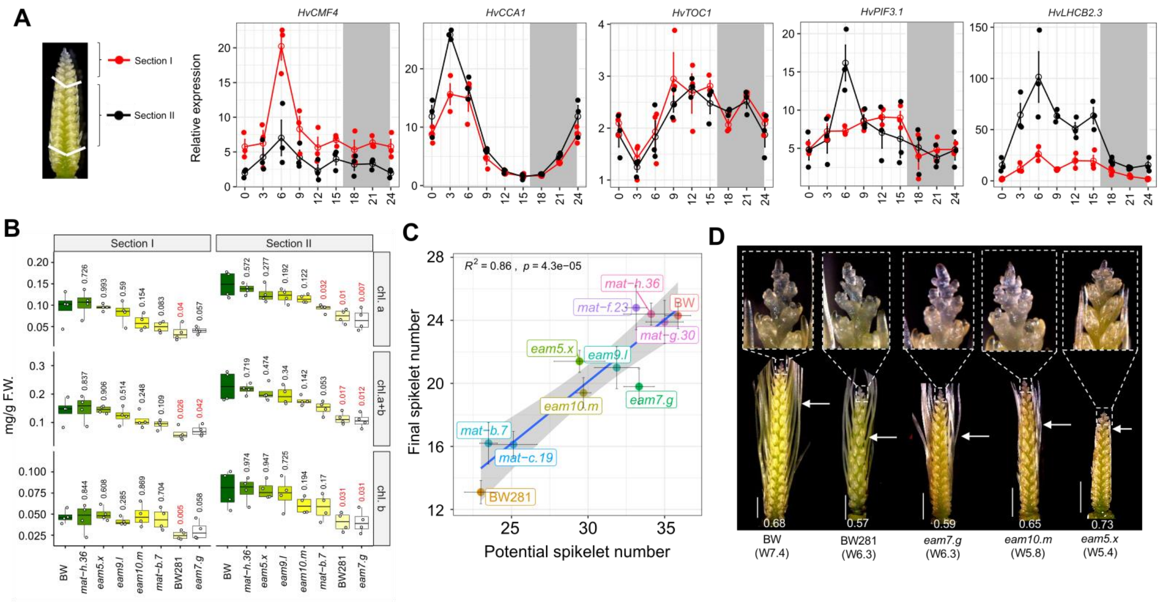

Fig. S18. Tissue-specific clock and clock output genes rhythmic expression is associated with variations of chlorophyll content and spikelet survival. (**A**) RT-qPCR results showing rhythmic expression patterns of *HvCMF4*, two exemplified clock genes (*HvCCA1* and *HvTOC1*) and clock output genes (*HvPIF3* and *HvLHCB2;3*) in WT spike sections at W5.5 stage in a diel cycle (LD). Grey shadow denotes night condition. Spike sections were collected every 4h within 24h under a light:dark condition of 16:8. Data are shown as mean ± s.e.m from three biological replicates, with each replicate from a pool of 5 spike sections. Sample collection for each time point was done within 30 min (~2 min per spike) to minimize the effects of extra light exposure under the microscope. Actin was used for normalization. (**B**) Quantification of chlorophyll content from spike sections at W5.5 stage in seven early flowering genotypes and the wild-type BW. Relative position used for spike sectioning is done according to (A). Significance levels above boxplots are determined from two-tailed Student's *t*-test using BW as the reference group, with each genotype containing 4 biological replicates. The significant changes are highlighted with red color. (**C**) Relationship between potential spikelet number and final spikelet number in the ten early maturity mutants mentioned above (Fig. S1A). Note that both BW281 and *eam7.g* deviate below the squares line, therefore have lower spikelet survival than BW. (**D**) Representative spike images from BW and 4 early flowering genotypes at MYP or abortion stage under LD condition. Arrows point to the breakpoint between survive and dying spikelets. While spikelet survival in *eam10.m* (*hvlux*) is comparable with BW, it shows early triggering of the spikelet degeneration and death program as seen in *tst2.b*; on the other hand, *eam5.x* (*hvphyc*), which shows higher spikelet survival compared with BW, also shows early promotion of spikelet development from the apical part of a spike. Scale bars = 2 mm.

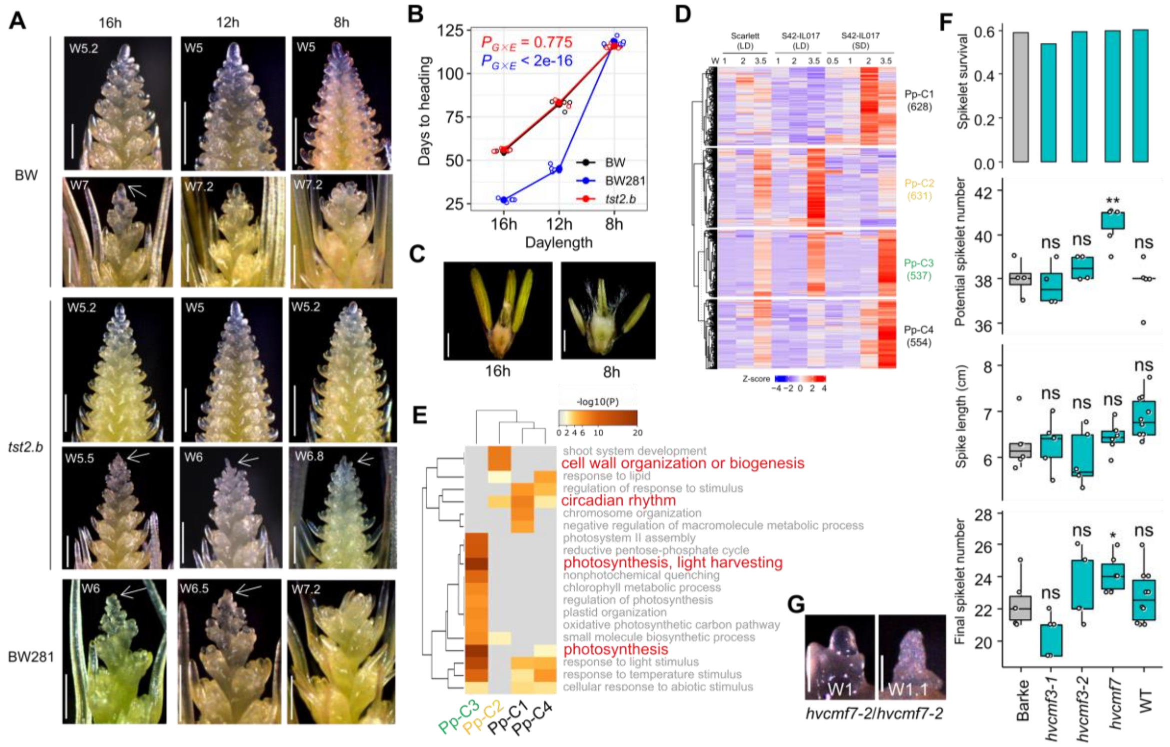

Fig. S19. The effects of different day-length on spikelet initiation and growth. (**A**, **B**) Spikelet growth and days to heading have different day-length sensitivity. Note that the development of distal spikelet from both BW281 and BW, but not *tst2.b*, are promoted under short day conditions (arrows), but days to heading remains the same in *tst2.b* related to BW. The significance of Genotype (G) × Environment (E) interaction in (B) is determined by ANOVA. (**C**) Anther developmental defects in BW under 8h daylength condition (see Figure 5D). (**D**, **E**) Transcriptional response to shorten day-length in the apex of a *PPD-H1* line (S41-IL107) and its wild-type background (Scarlett). Data are from (*36*). The heatmap shows four clusters (Pp-C1 – Pp-C4) with characteristic responses to shorten day-length at W3.5 (D), and the GO enriched biological pathways for each cluster are shown in (E). Either Pp-C2 related to vascularization or Pp-C3 for chloroplast development, are down-regulated or up-regulated under SD condition, which is consistent with both the increased spikelet survival under SD and their dysregulations in *tst2.b*. (**F**, **G**) Spikelet survival is not, or mildly affected in *hvcmf3* and *hvcmf7* mutants. Note that complete *HvCMF7* gene loss of function mutants die before the transition to reproductive growth (G). Significance levels are determined from two-tailed Student's *t*-test. $^*P < 0.05$; $^{**}P < 0.01$ and ns, not significant difference. Scale bars: 1 mm in (A and C); 200 μm in (G).

Table S1. Information of the barley germplasms used in this study

Accessions	Country	Genebank_status	Latitute[a]	Longtitute[a]	S284T	A256P	G128R	Hap_group	SS_GH	PSN_GH	SN_GH	SS_BLUES	PSN_BLUEs	SN_BLUEs
HOR_51	BGR	landrace	42.734	25.486	284S	256A	128G	Group2	0.59	98.39	58.51	0.64	102.73	61.03
HOR_133	GRC	landrace	39.074	21.824	284T	256A	128G	Group3	0.63	96.67	61.09	0.63	101.31	58.93
HOR_148	IND	landrace	20.594	78.963	284T	256A	128G	Group3	0.61	112.04	68.61	-	-	-
HOR_199	RUS	landrace	55.762	37.617	284S	256A	128G	Group2	0.61	125.47	76.55	0.65	117.81	72.11
HOR_264	USA	cultivar	37.090	-95.713	284T	256A	128G	Group3	0.68	106.93	72.95	0.70	103.58	66.81
HOR_448	TUR	landrace	38.964	35.243	284S	256A	128G	Group2	0.57	102.54	58.49	0.58	100.82	55.56
HOR_473	TUR	landrace	38.964	35.243	284S	256A	128G	Group2	0.56	106.93	59.93	0.62	100.41	59.28
HOR_495	TUR	landrace	38.964	35.243	284S	256A	128G	Group2	0.56	111.31	62.83	0.60	104.60	60.04
HOR_528	TUR	landrace	38.964	35.243	284S	256A	128G	Group2	0.57	101.81	57.76	0.66	89.58	56.68
HOR_534	TUR	landrace	38.964	35.243	284S	256A	128G	Group2	0.66	99.33	65.79	0.69	102.72	66.90
HOR_567	TUR	landrace	38.964	35.243	284S	256A	128G	Group2	0.50	92.32	46.19	0.63	92.39	56.78
HOR_693	GRC	landrace	39.074	21.824	284T	256A	128G	Group3	0.48	91.59	44.02	0.56	96.91	51.30
HOR_703	GRC	landrace	39.074	21.824	284T	256A	128G	Group3	0.54	97.43	52.70	0.55	97.19	51.93
HOR_730	GRC	landrace	39.074	21.824	284S	256A	128G	Group2	0.51	107.66	54.87	0.57	99.76	53.16
HOR_760	GRC	landrace	39.074	21.824	284S	256A	128G	Group2	0.58	136.14	78.74	0.59	115.00	63.55
HOR_770	ALB	landrace	41.153	20.168	284S	256A	128G	Group2	0.59	117.88	70.06	0.60	106.49	62.66
HOR_771	GRC	landrace	39.074	21.824	284S	256A	128G	Group2	0.62	135.66	83.87	0.68	114.22	75.44
HOR_772	ALB	landrace	41.153	20.168	284S	256A	128G	Group2	0.57	107.66	61.38	-	-	-
HOR_846	GRC	landrace	39.074	21.824	284S	256P	128R	Group1	0.49	93.78	46.19	0.51	101.36	49.76
HOR_872	IND	landrace	20.594	78.963	284T	256A	128G	Group3	0.65	70.41	45.47	0.67	89.92	56.82
HOR_1133	FRA	landrace	46.228	2.214	284T	256A	128G	Group3	0.62	115.69	72.23	0.54	111.47	58.74
HOR_1158	GRC	landrace	39.074	21.824	284S	256P	128R	Group1	0.47	108.31	50.60	0.58	96.45	52.96
HOR_1191	TUR	landrace	38.964	35.243	284S	256A	128G	Group2	0.64	98.89	63.55	0.66	99.34	63.26
HOR_1205	TUR	landrace	38.964	35.243	284S	256A	128G	Group2	0.55	106.93	59.21	0.66	99.35	62.86
HOR_1234	GRC	landrace	39.074	21.824	284S	256A	128G	Group2	0.53	101.08	53.42	0.59	91.00	51.99
HOR_1251	JPN	landrace	36.205	138.253	284T	256A	128G	Group3	0.65	119.42	77.29	-	-	-
HOR_1267	GRC	landrace	39.074	21.824	284S	256A	128G	Group2	0.51	109.12	55.59	0.60	97.54	55.69
HOR_1284	GRC	landrace	39.074	21.824	284S	256A	128G	Group2	0.70	100.35	70.06	0.62	108.46	65.23
HOR_1384	GRC	landrace	39.074	21.824	284T	256A	128G	Group3	0.60	117.62	70.13	0.64	113.16	70.70
HOR_1398	TUR	landrace	38.964	35.243	284S	256P	128R	Group1	0.58	104.62	60.72	0.60	95.20	55.74
HOR_1621	DEU	cultivar	51.166	10.452	284S	256P	128G	Others	0.54	113.74	61.09	0.54	112.80	59.33
HOR_1704	AFG	landrace	33.939	67.710	284S	256A	128G	Group2	0.64	103.28	66.44	0.62	90.20	57.11
HOR_1780	IRN	landrace	32.428	53.688	284T	256A	128G	Group3	0.61	96.70	59.21	0.57	101.73	56.43
HOR_1816	CHN	landrace	39.914	116.364	284S	256P	128R	Group1	0.50	90.13	44.74	0.72	104.68	75.39
HOR_1858	CHN	landrace	39.914	116.364	284S	256P	128R	Group1	0.53	118.04	62.89	0.57	104.00	59.33
HOR_1860	GRC	landrace	39.074	21.824	284S	256A	128G	Group2	0.49	100.83	49.87	0.59	101.48	57.78
HOR_1861	TUR	landrace	38.964	35.243	284S	256A	128G	Group2	0.61	123.72	75.12	0.61	120.31	72.48
HOR_1949	TUR	landrace	38.964	35.243	284S	256A	128G	Group2	0.54	107.75	58.14	0.69	93.97	63.41
HOR_1954	TUR	landrace	38.964	35.243	284S	256A	128G	Group2	0.64	104.66	66.51	0.64	105.07	64.33
HOR_1972	TUR	landrace	38.964	35.243	284S	256A	128G	Group2	0.58	117.49	67.96	0.62	102.49	60.22
HOR_1977	-	landrace	-	-	284T	256A	128G	Group3	0.65	87.70	57.11	0.64	84.94	53.93
HOR_2024	JPN	cultivar	36.205	138.253	284T	256A	128G	Group3	0.63	110.70	69.92	0.58	99.93	54.99
HOR_2367	CHN	cultivar	39.914	116.364	284T	256A	128G	Group3	0.70	81.08	57.11	0.69	98.59	65.56
HOR_2369	CHN	cultivar	39.914	116.364	284T	256A	128G	Group3	0.73	103.82	75.91	0.67	105.63	68.81

Accessions	Country	Genebank_status	Latitute[a]	Longtitute[a]	S284T	A256P	G128R	Hap_group	SS_GH	PSN_GH	SN_GH	SS_BLUES	PSN_BLUEs	SN_BLUEs
HOR_2383	CHN	landrace	39.914	116.364	284S	256P	128R	Group1	0.60	116.20	69.92	0.68	105.11	69.93
HOR_2403	CAN	cultivar	56.130	-106.347	284S	256A	128G	Group2	0.79	85.22	67.72	0.70	76.79	57.19
HOR_2503	CHN	landrace	39.914	116.364	284T	256A	128G	Group3	0.77	82.59	63.62	0.72	84.12	60.48
HOR_2520	GTM	landrace	15.783	-90.231	284S	256A	128G	Group2	0.56	102.78	57.11	0.58	98.09	55.33
HOR_2747	USA	cultivar	37.090	-95.713	284S	256A	128G	Group2	0.62	108.73	67.23	0.72	106.27	74.96
HOR_2749	USA	cultivar	37.090	-95.713	284S	256A	128G	Group2	0.72	87.94	63.55	0.71	103.07	69.55
HOR_2799	IRN	landrace	32.428	53.688	284S	256A	128G	Group2	0.62	103.28	63.62	0.64	92.67	57.81
HOR_2805	IRN	landrace	32.428	53.688	284S	256A	128G	Group2	0.57	108.31	62.17	0.68	96.36	64.19
HOR_2860	IRN	landrace	32.428	53.688	284S	256P	128R	Group1	0.61	107.55	65.79	0.65	99.20	64.22
HOR_3272	IND	cultivar	20.594	78.963	284T	256A	128G	Group3	0.55	85.75	46.91	0.61	88.76	52.52
HOR_3661	-	landrace	-	-	284T	256A	128G	Group3	0.66	132.61	86.86	0.71	131.26	89.48
HOR_4018	USA	cultivar	37.090	-95.713	284T	256A	128G	Group3	0.56	102.54	57.76	0.60	96.28	56.81
HOR_4058	MNG	landrace	46.862	103.847	284T	256A	128G	Group3	0.66	99.87	65.79	0.61	113.08	65.57
HOR_4061	CHN	cultivar	39.914	116.364	284S	256A	128G	Group2	0.56	91.06	51.32	0.68	88.23	60.41
HOR_4065	USA	cultivar	37.090	-95.713	284S	256A	128G	Group2	0.50	114.49	57.11	0.58	123.99	68.51
HOR_4091	MNG	landrace	46.862	103.847	284T	256A	128G	Group3	0.65	105.24	68.68	0.63	103.85	63.89
HOR_4114	USA	cultivar	37.090	-95.713	284S	256A	128G	Group2	0.56	125.76	70.13	0.56	119.18	62.33
HOR_4146	DZA	landrace	28.034	1.660	284S	256A	128G	Group2	0.61	115.69	70.06	0.62	108.26	63.67
HOR_4420	MNG	landrace	46.862	103.847	284T	256A	128G	Group3	0.62	117.54	73.02	0.65	114.43	70.22
HOR_4421	MNG	landrace	46.862	103.847	284T	256A	128G	Group3	0.72	107.04	77.36	0.74	109.96	75.15
HOR_4588	MNG	landrace	46.862	103.847	284T	256A	128G	Group3	0.66	102.54	67.89	0.65	101.78	67.17
HOR_4815	DNK	landrace	56.264	9.502	284T	256A	128G	Group3	0.65	92.66	60.00	0.59	98.60	57.15
HOR_4829	USA	landrace	37.090	-95.713	284S	256P	128R	Group1	0.60	129.57	78.02	0.66	112.95	70.00
HOR_5185	ETH	landrace	9.145	40.490	284T	256A	128G	Group3	0.64	82.83	52.70	0.62	92.91	54.62
HOR_5220	ETH	landrace	9.145	40.490	284T	256A	128G	Group3	0.60	106.20	63.55	0.61	107.94	63.26
HOR_5876	ETH	landrace	9.145	40.490	284S	256A	128G	Group2	0.57	96.99	54.94	0.66	102.07	62.33
HOR_6876	ETH	landrace	9.145	40.490	284S	256A	128G	Group2	0.63	110.39	69.40	0.62	115.96	70.81
HOR_6908	ETH	landrace	9.145	40.490	284S	256A	128G	Group2	0.70	103.28	72.23	0.66	108.68	69.00
HOR_6939	ARG	cultivar	-38.416	-63.617	284S	256A	128G	Group2	0.56	122.07	67.96	0.60	113.23	67.67
HOR_6954	GRC	landrace	39.074	21.824	284S	256A	128G	Group2	0.51	96.62	49.15	0.54	93.89	49.70
HOR_6962	IND	cultivar	20.594	78.963	284T	256A	128G	Group3	0.60	101.81	60.66	0.69	99.26	60.74
HOR_6999	NPL	landrace	28.395	84.124	284T	256A	128G	Group3	0.61	114.01	69.40	0.66	110.71	69.77
HOR_7067	NPL	landrace	28.395	84.124	284S	256A	128G	Group2	0.56	107.58	60.72	0.64	95.47	58.26
HOR_7116	NPL	landrace	28.395	84.124	284S	256P	128R	Group1	0.61	100.75	61.45	0.69	91.40	59.26
HOR_7134	NPL	landrace	28.395	84.124	284S	256P	128R	Group1	0.68	85.83	58.51	0.67	84.66	55.87
HOR_7142	NPL	landrace	28.395	84.124	284S	256P	128R	Group1	0.62	91.14	56.38	0.64	83.66	52.70
HOR_7164	NPL	landrace	28.395	84.124	284T	256A	128G	Group3	0.58	84.18	48.43	0.59	83.25	47.67
HOR_7171	NPL	landrace	28.395	84.124	284S	256P	128R	Group1	0.73	95.97	70.06	0.74	96.21	67.22
HOR_7230	IND	landrace	20.594	78.963	284S	256P	128R	Group1	0.55	72.31	39.75	0.60	78.20	45.26
HOR_7251	IND	landrace	20.594	78.963	284T	256A	128G	Group3	0.64	104.53	66.51	0.67	106.48	68.56
HOR_7428	RUS	landrace	55.762	37.617	284T	256A	128G	Group3	0.75	123.72	92.48	-	-	-
HOR_7571	PAK	landrace	30.375	69.345	284T	256A	128G	Group3	0.63	91.09	57.83	0.68	99.77	64.15
HOR_7602	PAK	landrace	30.375	69.345	284T	256A	128G	Group3	0.56	107.59	60.00	0.58	123.59	69.48
HOR_7629	PAK	landrace	30.375	69.345	284S	256P	128R	Group1	0.58	110.62	63.62	0.64	108.38	67.48

Accessions	Country	Genebank_status	Latitute[a]	Longtitute[a]	S284T	A256P	G128R	Hap_group	SS_GH	PSN_GH	SN_GH	SS_BLUES	PSN_BLUEs	SN_BLUEs
HOR_7647	PAK	landrace	30.375	69.345	284T	256A	128G	Group3	0.60	97.61	58.55	0.62	104.05	63.74
HOR_7657	PAK	landrace	30.375	69.345	284S	256P	128R	Group1	0.62	80.66	49.87	0.69	90.40	61.84
HOR_7679	PAK	landrace	30.375	69.345	284S	256P	128R	Group1	0.51	80.45	41.19	0.64	92.31	58.11
HOR_7973	TUR	landrace	38.964	35.243	284S	256A	128G	Group2	0.57	113.12	64.34	0.64	104.70	64.63
HOR_8338	IND	landrace	20.594	78.963	284T	256A	128G	Group3	0.59	96.98	57.11	0.67	104.22	64.81
HOR_8347	IND	landrace	20.594	78.963	284T	256A	128G	Group3	0.67	82.16	54.94	0.66	93.63	57.70
HOR_8355	IND	landrace	20.594	78.963	284S	256P	128R	Group1	0.59	113.22	66.51	0.62	111.80	67.37
HOR_8370	IND	landrace	20.594	78.963	284T	256A	128G	Group3	0.60	89.57	53.49	0.64	94.84	59.00
HOR_8391	IND	landrace	20.594	78.963	284T	256A	128G	Group3	0.60	81.71	49.15	0.59	80.82	48.33
HOR_8428	IND	landrace	20.594	78.963	284T	256A	128G	Group3	0.64	109.27	70.13	0.69	102.29	67.26
HOR_8446	IND	landrace	20.594	78.963	284T	256A	128G	Group3	0.67	71.87	48.36	0.66	76.92	50.22
HOR_8451	IND	landrace	20.594	78.963	284T	256A	128G	Group3	0.61	71.87	44.02	-	-	-
HOR_8454	IRQ	landrace	33.223	43.679	284T	256A	128G	Group3	0.58	82.85	48.43	0.72	75.21	53.52
HOR_8711	NPL	landrace	28.395	84.124	284S	256P	128R	Group1	0.68	83.11	56.38	0.71	92.33	62.41
HOR_8751	NPL	landrace	28.395	84.124	284S	256A	128G	Group2	0.62	92.14	57.11	0.63	110.08	65.96
HOR_8787	NPL	landrace	28.395	84.124	284T	256A	128G	Group3	0.68	90.86	62.10	0.71	89.94	64.19
HOR_8879	USA	cultivar	37.090	-95.713	284S	256A	128G	Group2	0.54	109.12	58.49	0.66	100.56	62.52
HOR_9043	ETH	landrace	9.145	40.490	284S	256A	128G	Group2	0.54	88.62	48.20	0.58	88.99	51.11
HOR_9292	RUS	cultivar	55.762	37.617	284S	256A	128G	Group2	0.65	125.66	81.71	0.65	115.41	73.30
HOR_9532	USA	cultivar	37.090	-95.713	284T	256A	128G	Group3	0.60	99.76	60.00	0.69	101.15	67.96
HOR_9558	CHN	landrace	39.914	116.364	284T	256A	128G	Group3	0.65	87.50	57.11	0.63	100.03	60.26
HOR_9559	COL	landrace	4.571	-74.297	284S	256A	128G	Group2	0.56	93.37	52.04	0.64	97.26	58.22
HOR_9573	USA	cultivar	37.090	-95.713	284S	256P	CT	Het	0.56	104.11	58.55	0.62	99.37	59.59
HOR_9709	ITA	landrace	41.872	12.567	284S	256A	128G	Group2	0.59	114.57	67.23	0.60	112.14	64.07
HOR_9720	LBY	landrace	26.335	17.228	284S	256P	128R	Group1	0.55	88.67	49.08	0.65	84.84	50.79
HOR_9841	LBY	landrace	26.335	17.228	284S	256A	128G	Group2	0.69	82.82	57.41	0.69	95.06	64.31
HOR_9876	LBY	landrace	26.335	17.228	284S	256P	128R	Group1	0.51	79.91	41.13	0.58	83.16	45.03
HOR_9891	LBY	landrace	26.335	17.228	284S	256P	128R	Group1	0.54	79.17	42.57	0.59	79.43	45.30
HOR_9911	ITA	landrace	41.872	12.567	284S	256A	128G	Group2	0.57	121.09	68.68	0.60	106.67	62.33
HOR_9930	LBY	landrace	26.335	17.228	284S	256A	128G	Group2	0.57	93.05	52.70	0.58	89.11	48.57
HOR_10004	PAK	landrace	30.375	69.345	284S	256P	128R	Group1	0.58	89.40	51.98	0.61	88.19	52.41
HOR_10014	PAK	landrace	30.375	69.345	284S	256A	128G	Group2	0.59	73.67	43.36	0.61	87.86	51.16
HOR_10018	PAK	landrace	30.375	69.345	284T	256A	128G	Group3	0.51	104.01	53.42	0.62	90.90	54.33
HOR_10074	MNG	landrace	46.862	103.847	284T	256A	128G	Group3	0.59	106.29	62.89	0.60	111.88	64.22
HOR_10083	LBY	landrace	26.335	17.228	284S	256P	128R	Group1	0.51	94.63	48.43	0.58	85.40	47.59
HOR_10087	LBY	landrace	26.335	17.228	284S	256P	128G	Others	0.54	69.96	37.58	0.57	89.36	48.44
HOR_10157	LBY	landrace	26.335	17.228	284T	256A	128G	Group3	0.53	74.06	38.96	0.57	79.33	43.19
HOR_10560	ITA	landrace	41.872	12.567	284S	256A	128G	Group2	0.47	108.58	50.60	0.60	99.18	58.26
HOR_10606	IRQ	landrace	33.223	43.679	284S	256A	128G	Group2	0.62	108.39	67.17	0.63	110.75	67.81
HOR_10787	ITA	landrace	41.872	12.567	284S	256A	128G	Group2	0.56	119.53	67.23	0.57	113.40	63.93
HOR_10793	ITA	landrace	41.872	12.567	284S	256A	128G	Group2	0.54	119.72	65.06	0.57	118.45	66.33
HOR_11056	PRK	cultivar	40.340	127.510	284T	256A	128G	Group3	0.64	102.41	65.79	0.69	105.29	69.22
HOR_11111	NPL	landrace	28.395	84.124	284S	256P	128R	Group1	0.71	87.94	62.10	0.65	99.04	59.48
HOR_11173	TUN	landrace	33.887	9.537	284S	256P	128R	Group1	0.51	96.70	49.08	0.68	96.24	61.03

Accessions	Country	Genebank_status	Latitute[a]	Longtitute[a]	S284T	A256P	G128R	Hap_group	SS_GH	PSN_GH	SN_GH	SS_BLUES	PSN_BLUEs	SN_BLUEs
HOR_11174	TUN	landrace	33.887	9.537	284S	256P	128R	Group1	0.54	97.43	52.70	0.63	95.27	57.44
HOR_11407	CHN	landrace	39.914	116.364	284T	256A	128G	Group3	0.56	131.55	73.74	0.66	120.08	74.35
HOR_11409	KOR	cultivar	35.908	127.767	284T	256A	128G	Group3	0.65	77.05	49.87	0.68	94.38	63.04
HOR_11419	IND	landrace	20.594	78.963	284S	CG	128G	Het	0.66	105.47	70.06	0.68	106.19	69.04
HOR_11420	IND	landrace	20.594	78.963	284S	256P	128R	Group1	0.60	111.62	67.23	0.69	91.58	62.30
HOR_11430	PER	landrace	-9.190	-75.015	284S	256A	128G	Group2	0.59	116.42	68.61	0.63	113.93	68.22
HOR_11431	PER	landrace	-9.190	-75.015	284S	256A	128G	Group2	0.52	133.31	69.40	0.62	124.68	75.15
HOR_11448	USA	landrace	37.090	-95.713	284T	256A	128G	Group3	0.59	119.34	70.78	0.60	110.35	63.44
HOR_11450	USA	landrace	37.090	-95.713	284T	256A	128G	Group3	0.63	123.34	78.08	0.61	119.80	72.81
HOR_11480	ITA	landrace	41.872	12.567	284S	256A	128G	Group2	0.48	95.24	46.19	0.57	98.49	52.74
HOR_12402	ALB	landrace	41.153	20.168	284S	256A	128G	Group2	0.65	108.31	70.85	0.68	115.55	74.93
HOR_12412	ITA	landrace	41.872	12.567	284T	256A	128G	Group3	0.67	113.78	76.64	0.64	121.13	75.56
HOR_12791	ITA	landrace	41.872	12.567	284S	256A	128G	Group2	0.58	109.12	62.83	0.68	98.77	66.59
HOR_12828	SYR	landrace	34.802	38.997	284S	256A	128G	Group2	0.49	87.94	43.30	0.59	95.02	53.13
HOR_13426	MAR	landrace	31.792	-7.093	284S	256A	128G	Group2	0.54	103.52	55.66	0.62	98.24	56.85
HOR_13716	IND	landrace	20.594	78.963	284T	256A	128G	Group3	0.64	74.34	47.46	0.65	95.62	62.07
HOR_13724	IND	landrace	20.594	78.963	284T	256A	128G	Group3	0.68	81.37	55.59	0.67	90.29	57.11
HOR_13987	ETH	landrace	9.145	40.490	284S	256A	128G	Group2	0.56	109.32	61.09	0.64	104.22	63.60
HOR_14235	PAK	landrace	30.375	69.345	284T	256A	128G	Group3	0.54	106.93	57.76	0.63	109.84	64.44
HOR_14342	AFG	landrace	33.939	67.710	284T	256A	128G	Group3	0.52	84.56	44.09	0.58	82.46	46.81
HOR_14382	ESP	landrace	40.464	-3.749	284T	256A	128G	Group3	0.59	126.20	74.47	0.60	112.46	64.44
HOR_14689	USA	landrace	37.090	-95.713	284S	256A	128G	Group2	0.59	91.70	54.21	0.69	92.05	58.78
HOR_14710	-	cultivar	-	-	284S	256P	128R	Group1	0.58	101.76	59.28	0.68	94.32	61.96
HOR_14730	ETH	landrace	9.145	40.490	284S	256A	128G	Group2	0.50	112.04	56.32	0.58	116.06	64.58
HOR_14776	CAN	landrace	56.130	-106.347	284S	256A	128G	Group2	0.54	105.28	56.38	0.64	99.78	60.85
HOR_14817	-	cultivar	-	-	284S	256P	128G	Others	0.44	105.02	46.26	0.58	100.06	56.33
HOR_14909	NPL	landrace	28.395	84.124	284S	256P	128R	Group1	0.58	88.67	51.25	0.67	93.10	60.22
HOR_14943	NPL	landrace	28.395	84.124	284S	256P	128R	Group1	0.60	100.67	60.00	0.68	90.89	62.33
HOR_15119	DEU	cultivar	51.166	10.452	284S	256A	128G	Group2	0.59	111.31	65.72	0.63	102.11	63.11
HOR_15124	DEU	landrace	51.166	10.452	284S	256A	128G	Group2	0.59	103.35	61.45	0.66	103.82	65.96
HOR_15154	FIN	landrace	61.924	25.748	284S	256A	128G	Group2	0.71	98.16	70.06	0.69	111.97	71.78
HOR_15224	ETH	landrace	9.145	40.490	284S	256A	128G	Group2	0.50	100.35	49.81	0.53	109.15	53.31
HOR_15238	IRN	landrace	32.428	53.688	284S	256A	128G	Group2	0.59	109.39	65.06	0.64	107.83	66.96
HOR_15384	CHN	landrace	39.914	116.364	284S	256P	128R	Group1	0.63	128.39	80.98	0.68	112.16	75.37
HOR_15401	PAK	landrace	30.375	69.345	284T	256A	128G	Group3	0.50	103.24	52.04	0.58	94.40	53.74
HOR_15412	PAK	landrace	30.375	69.345	284T	256A	128G	Group3	0.57	105.47	59.93	0.56	93.10	52.89
HOR_15423	PAK	landrace	30.375	69.345	284T	256A	128G	Group3	0.47	116.20	54.21	0.52	111.46	56.89
HOR_15553	TUR	landrace	38.964	35.243	284S	256A	128G	Group2	0.59	104.18	61.45	0.62	104.34	63.89
HOR_15562	PAK	landrace	30.375	69.345	284T	256A	128G	Group3	0.56	85.63	47.70	0.66	85.51	52.78
HOR_15704	AFG	landrace	33.939	67.710	284S	256A	128G	Group2	0.55	111.16	60.72	0.58	107.37	62.48
HOR_15758	IND	landrace	20.594	78.963	284S	256P	128R	Group1	0.55	97.72	53.49	0.70	82.01	52.53
HOR_15760	IND	landrace	20.594	78.963	284S	256P	128R	Group1	0.53	99.62	52.70	0.61	84.62	53.50
HOR_15778	AFG	landrace	33.939	67.710	284S	256A	128G	Group2	0.61	104.01	63.55	0.70	96.86	65.24
HOR_15860	PAK	landrace	30.375	69.345	284T	256A	128G	Group3	0.56	84.57	46.98	0.63	84.82	50.85

Accessions	Country	Genebank_status	Latitute[a]	Longtitute[a]	S284T	A256P	G128R	Hap_group	SS_GH	PSN_GH	SN_GH	SS_BLUES	PSN_BLUEs	SN_BLUEs
HOR_15871	PAK	landrace	30.375	69.345	284T	256A	128G	Group3	0.57	100.94	57.77	0.58	105.23	59.81
HOR_15889	RUS	landrace	55.762	37.617	284S	256A	128G	Group2	0.62	104.36	64.40	0.59	107.29	62.56
HOR_15890	AFG	landrace	33.939	67.710	284S	256P	128R	Group1	0.57	93.46	53.49	0.66	88.10	59.33
HOR_15894	CHE	landrace	46.818	8.228	284S	256P	128G	Others	0.62	118.90	73.74	0.60	131.55	78.16
HOR_15895	USA	landrace	37.090	-95.713	284S	256P	128R	Group1	0.55	82.32	45.62	0.61	91.91	50.74
HOR_15908	GRC	landrace	39.074	21.824	284S	256A	128G	Group2	0.53	108.90	57.83	0.59	98.11	56.89
HOR_15917	GRC	landrace	39.074	21.824	284S	256A	128G	Group2	0.56	80.54	44.81	0.60	88.30	52.96
HOR_15920	-	landrace	-	-	284S	256A	128G	Group2	0.55	97.78	53.72	0.58	97.67	52.59
HOR_15922	AFG	landrace	33.939	67.710	284T	256A	128G	Group3	0.61	115.69	70.06	0.74	106.96	74.70
HOR_16078	AFG	landrace	33.939	67.710	284S	256A	128G	Group2	0.64	111.29	71.57	0.66	121.64	76.26
HOR_16087	ISR	landrace	31.046	34.852	284S	256A	128G	Group2	0.54	98.16	52.70	0.61	100.18	57.78
HOR_16261	IND	landrace	20.594	78.963	284T	256A	128G	Group3	0.54	82.30	44.09	0.63	82.70	48.67
HOR_16569	IND	landrace	20.594	78.963	284T	256A	128G	Group3	0.63	76.03	48.20	0.67	91.50	58.19
HOR_17386	NPL	landrace	28.395	84.124	284S	256A	128G	Group2	0.57	78.70	44.81	0.74	78.60	57.22
HOR_17517	-	cultivar	-	-	284S	256A	128G	Group2	0.53	114.56	61.09	0.57	117.45	65.63
HOR_17574	-	landrace	-	-	284T	256A	128G	Group3	0.63	93.05	58.49	0.57	103.28	56.70
HOR_17654	-	cultivar	-	-	284S	256P	128R	Group1	0.51	98.16	49.81	0.59	103.02	57.04
HOR_17705	-	landrace	-	-	284T	256A	128G	Group3	0.63	94.65	59.28	0.60	98.11	57.56
HOR_18227	JPN	landrace	36.205	138.253	284S	256A	128G	Group2	0.59	114.23	67.17	0.60	108.87	63.40
HOR_18267	GRC	landrace	39.074	21.824	284S	256A	128G	Group2	0.56	118.63	66.61	0.64	110.00	67.17
HOR_18381	MEX	cultivar	23.635	-102.553	284S	256A	128G	Group2	0.70	86.48	60.66	0.70	91.92	61.40
HOR_18385	MEX	cultivar	23.635	-102.553	284S	256A	128G	Group2	0.69	80.64	55.59	0.72	95.45	63.90
HOR_18824	JPN	landrace	36.205	138.253	284T	256A	128G	Group3	0.64	84.29	54.15	0.73	89.36	62.79
HOR_18827	JPN	landrace	36.205	138.253	284T	256A	128G	Group3	0.61	108.67	66.51	0.64	107.27	70.07
HOR_18917	-	landrace	-	-	284S	256P	128R	Group1	0.55	91.59	50.53	0.65	91.38	55.35
HOR_18920	DEU	landrace	51.166	10.452	284T	256A	128G	Group3	0.74	117.37	86.76	0.65	118.57	75.04
HOR_19129	GBR	cultivar	55.378	-3.436	284S	256A	128G	Group2	0.60	106.93	64.27	0.62	115.15	69.08
HOR_19168	IND	landrace	20.594	78.963	284T	256A	128G	Group3	0.51	100.35	51.25	0.58	92.08	52.20
HOR_19184	IND	landrace	20.594	78.963	284T	256A	128G	Group3	0.58	71.14	41.13	-	-	-
HOR_19581	ETH	landrace	9.145	40.490	284S	256A	128G	Group2	0.65	99.62	65.00	0.63	106.36	64.99
HOR_19776	JPN	cultivar	36.205	138.253	284T	256A	128G	Group3	0.63	87.94	55.59	0.67	97.96	58.98
HOR_20710	IRN	landrace	32.428	53.688	284S	256A	128G	Group2	0.57	95.24	54.15	0.63	103.27	57.79
HOR_20714	RUS	landrace	55.762	37.617	284T	256A	128G	Group3	0.72	85.02	61.38	0.67	93.40	58.90
HOR_21087	-	landrace	-	-	284T	256A	128G	Group3	0.66	71.14	46.91	0.58	77.97	47.04
HOR_21250	IRN	landrace	32.428	53.688	284S	256A	128G	Group2	0.55	112.60	61.46	0.59	99.10	56.01
HOR_21344	IRN	landrace	32.428	53.688	284S	256P	128R	Group1	0.50	90.13	45.47	0.49	94.25	46.02
HOR_21436	IRN	landrace	32.428	53.688	284S	256P	128R	Group1	0.53	91.59	48.36	0.60	90.01	50.18
HOR_21439	IRN	landrace	32.428	53.688	284S	256A	128G	Group2	0.62	100.23	62.17	0.63	107.56	63.44
HOR_21641	TCD	landrace	15.454	18.732	284S	256P	128R	Group1	0.56	96.81	54.46	0.56	94.75	51.56
HOR_924	TUR	landrace	38.964	35.243	284S	256A	128G	Group2	0.53	119.34	63.55	0.61	113.12	67.23
HOR_1175	TUR	landrace	38.964	35.243	284S	256P	128R	Group1	0.55	88.67	48.36	0.57	91.68	50.56
HOR_1176	TUR	landrace	38.964	35.243	284S	256A	128G	Group2	0.60	90.13	54.15	0.64	92.24	57.73
HOR_2004	ITA	landrace	41.872	12.567	284S	256P	128R	Group1	0.51	106.93	54.15	0.55	104.38	55.29
HOR_2366	CHN	cultivar	39.914	116.364	284T	256A	128G	Group3	0.67	97.43	65.72	0.64	109.97	68.06

Accessions	Country	Genebank_status	Latitute[a]	Longtitute[a]	S284T	A256P	G128R	Hap_group	SS_GH	PSN_GH	SN_GH	SS_BLUES	PSN_BLUEs	SN_BLUEs
HOR_4413	IND	cultivar	20.594	78.963	284S	256P	128R	Group1	0.50	149.29	74.40	-	-	-
HOR_10778	ITA	landrace	41.872	12.567	284S	256A	128G	Group2	0.57	119.34	68.61	0.60	111.89	64.01
HOR_10843	COL	landrace	4.571	-74.297	284S	256P	128R	Group1	0.57	106.93	61.38	0.60	102.30	59.06
HOR_10844	COL	landrace	4.571	-74.297	284S	256A	128G	Group2	0.58	100.35	57.76	0.65	94.77	58.79
HOR_10845	COL	landrace	4.571	-74.297	284T	256A	128G	Group3	0.61	87.94	53.42	0.66	103.70	66.51
HOR_10847	USA	cultivar	37.090	-95.713	284S	256A	128G	Group2	0.70	117.88	82.15	0.76	112.83	81.92
HOR_10961	USA	cultivar	37.090	-95.713	284S	256A	128G	Group2	0.54	98.16	53.42	0.53	95.24	47.90
HOR_10983	TUN	landrace	33.887	9.537	284S	256A	128G	Group2	0.49	85.75	41.85	0.51	88.55	43.34
HOR_10984	TUN	landrace	33.887	9.537	284S	256A	128G	Group2	0.46	98.16	45.47	0.52	94.08	46.79
HOR_10985	TUN	landrace	33.887	9.537	284S	256A	128G	Group2	0.51	102.54	52.70	0.58	98.63	55.01
HOR_11016	-	cultivar	-	-	284S	256P	128R	Group1	0.47	99.62	46.91	0.57	95.29	51.90
HOR_11053	PRK	cultivar	40.340	127.510	284T	256A	128G	Group3	0.67	82.83	55.59	0.72	87.46	61.06
HOR_11065	PRK	cultivar	40.340	127.510	284S	256A	128G	Group2	0.70	87.94	61.38	0.64	97.29	60.29
HOR_11093	PRK	cultivar	40.340	127.510	284T	256A	128G	Group3	0.71	117.15	83.08	-	-	-
HOR_11124	ITA	landrace	41.872	12.567	284S	256A	128G	Group2	0.51	122.26	62.83	0.57	121.30	66.23
HOR_11154	PRK	landrace	40.340	127.510	284T	256A	128G	Group3	0.67	68.95	46.19	0.67	84.55	54.01
HOR_11176	TUN	landrace	33.887	9.537	284S	256A	128G	Group2	0.48	114.23	54.87	0.55	99.86	52.68
HOR_11258	JPN	cultivar	36.205	138.253	284S	256A	128G	Group2	0.67	104.01	70.06	0.70	97.10	64.95
HOR_11267	JPN	breeding_material	36.205	138.253	284S	256A	128G	Group2	0.67	132.49	88.70	-	-	-
HOR_11268	JPN	breeding_material	36.205	138.253	284S	256A	128G	Group2	0.67	112.77	75.12	-	-	-
HOR_11298	KOR	cultivar	35.908	127.767	284S	256A	128G	Group2	0.73	83.56	60.66	0.63	94.19	57.56
HOR_11301	JPN	cultivar	36.205	138.253	284T	256A	128G	Group3	0.70	121.53	84.53	-	-	-
HOR_11306	KOR	cultivar	35.908	127.767	284S	256A	128G	Group2	0.73	81.37	59.21	0.71	92.80	63.23
HOR_11308	CHN	cultivar	39.914	116.364	284S	256A	128G	Group2	0.70	99.62	70.06	-	-	-
HOR_11318	ISR	landrace	31.046	34.852	284S	256A	128G	Group2	0.51	95.24	48.36	0.57	95.37	51.79
HOR_11319	GRC	landrace	39.074	21.824	284S	256A	128G	Group2	0.52	107.66	55.59	0.54	107.20	55.51
HOR_11321	TUN	landrace	33.887	9.537	284T	256A	128G	Group3	0.52	82.10	42.57	0.59	82.60	47.62
HOR_11322	TUN	landrace	33.887	9.537	284T	256A	128G	Group3	0.54	83.56	44.74	0.58	81.04	44.56
HOR_11324	TUN	landrace	33.887	9.537	284S	256P	128R	Group1	0.48	82.10	39.68	0.59	83.19	46.34
HOR_11325	TUN	landrace	33.887	9.537	284T	256A	128G	Group3	0.45	97.43	44.02	0.62	91.25	54.45
HOR_11326	TUN	landrace	33.887	9.537	284S	256P	128R	Group1	0.55	103.28	56.32	0.59	97.15	54.73
HOR_11329	TUN	landrace	33.887	9.537	284S	256P	128R	Group1	0.47	94.51	44.74	0.59	91.51	51.45
HOR_11331	TUN	landrace	33.887	9.537	284T	256A	128G	Group3	0.51	78.44	39.68	0.56	79.04	43.06
HOR_11332	TUN	landrace	33.887	9.537	284T	256A	128G	Group3	0.46	85.75	39.68	0.59	87.42	49.45
HOR_11333	TUN	landrace	33.887	9.537	284T	256A	128G	Group3	0.53	79.17	41.85	0.61	82.46	47.79
HOR_11334	TUN	landrace	33.887	9.537	284S	256A	128G	Group2	0.55	82.10	45.47	0.59	89.12	49.95
HOR_11346	TUN	landrace	33.887	9.537	284S	256P	128R	Group1	0.62	96.70	59.93	0.66	100.64	63.34
HOR_11349	TUN	landrace	33.887	9.537	284S	256P	128R	Group1	0.63	94.51	59.21	0.57	93.11	51.23
HOR_11351	TUN	landrace	33.887	9.537	284S	256A	128G	Group2	0.54	95.97	51.98	0.61	93.07	54.84
HOR_11381	-	cultivar	-	-	284S	256P	128R	Group1	0.52	93.05	48.36	0.52	94.20	46.90
HOR_11388	-	cultivar	-	-	284S	256A	128G	Group2	0.60	104.01	62.83	0.61	106.01	62.84
HOR_11400	ETH	landrace	9.145	40.490	284S	256P	128R	Group1	0.62	90.86	56.32	0.63	98.58	59.68
HOR_11454	USA	cultivar	37.090	-95.713	284S	256P	128R	Group1	0.77	82.10	63.55	0.66	98.43	62.12
HOR_11489	ITA	landrace	41.872	12.567	284S	256A	128G	Group2	0.55	101.81	56.32	0.62	95.23	56.62

Accessions	Country	Genebank_status	Latitute[a]	Longtitute[a]	S284T	A256P	G128R	Hap_group	SS_GH	PSN_GH	SN_GH	SS_BLUES	PSN_BLUEs	SN_BLUEs
HOR_11490	ITA	landrace	41.872	12.567	284S	256P	128R	Group1	0.51	95.97	49.08	0.56	93.96	49.90
HOR_11491	ITA	landrace	41.872	12.567	284S	256A	128G	Group2	0.56	92.32	51.25	0.60	91.86	53.95
HOR_11492	ITA	landrace	41.872	12.567	284S	256A	128G	Group2	0.50	101.08	50.53	0.53	102.10	52.51
HOR_11494	ITA	landrace	41.872	12.567	284S	256A	128G	Group2	0.55	100.35	54.87	0.61	95.93	56.51
HOR_11496	ITA	landrace	41.872	12.567	284S	256A	128G	Group2	0.62	103.28	64.27	0.63	102.23	61.90
HOR_11498	ITA	landrace	41.872	12.567	284S	256A	128G	Group2	0.52	106.20	55.59	0.57	103.01	55.12
HOR_11503	ITA	landrace	41.872	12.567	284S	256A	128G	Group2	0.49	93.05	45.47	0.63	91.23	54.90
HOR_11504	ITA	landrace	41.872	12.567	284S	256A	128G	Group2	0.51	104.01	53.42	0.62	90.97	53.95
HOR_11517	UZB	landrace	41.377	64.585	284T	256A	128G	Group3	0.61	97.43	59.21	0.63	107.45	64.79
HOR_11933	JPN	cultivar	36.205	138.253	284S	256A	128G	Group2	0.66	100.35	66.44	0.64	106.80	66.23
HOR_12058	COL	cultivar	4.571	-74.297	284T	256A	128G	Group3	0.57	75.52	43.30	0.67	87.47	56.23
HOR_12423	ITA	landrace	41.872	12.567	284S	256P	128R	Group1	0.54	98.16	52.70	0.58	95.48	53.06
HOR_12522	MAR	landrace	31.792	-7.093	284S	256P	128R	Group1	0.58	89.40	51.98	0.60	85.61	48.84
HOR_12711	ITA	landrace	41.872	12.567	284S	256A	128G	Group2	0.48	104.74	49.81	0.54	103.90	53.84
HOR_12745	PRK	landrace	40.340	127.510	284T	256A	128G	Group3	0.73	127.38	92.48	0.78	101.24	77.23
HOR_12777	-	cultivar	-	-	284S	256A	128G	Group2	0.57	100.35	57.04	0.57	123.77	67.12
HOR_12783	TUN	landrace	33.887	9.537	284S	256P	128R	Group1	0.57	95.97	54.87	0.69	90.31	60.01
HOR_12788	JPN	landrace	36.205	138.253	284T	256A	128G	Group3	0.64	109.12	69.34	0.67	101.45	64.45
HOR_12789	TUN	landrace	33.887	9.537	284T	256A	128G	Group3	0.48	79.17	38.23	0.62	79.35	47.06
HOR_12790	TUN	landrace	33.887	9.537	284S	256P	128R	Group1	0.55	90.13	49.81	0.55	92.22	48.51
HOR_12796	CHN	landrace	39.914	116.364	284T	256A	128G	Group3	0.63	133.22	84.53	0.68	114.66	76.51
HOR_12822	ITA	landrace	41.872	12.567	284S	256A	128G	Group2	0.58	123.72	71.59	0.68	113.01	74.12
HOR_13153	ITA	landrace	41.872	12.567	284S	256A	128G	Group2	0.49	93.78	46.19	0.56	88.01	47.68
HOR_13160	MAR	landrace	31.792	-7.093	284S	256P	128R	Group1	0.53	90.13	47.64	0.59	88.87	50.51
HOR_13412	MAR	landrace	31.792	-7.093	284S	256P	128R	Group1	0.54	95.24	51.25	0.56	93.95	51.18
HOR_13420	MAR	landrace	31.792	-7.093	284S	256A	128G	Group2	0.50	95.97	47.64	0.58	95.01	52.84
HOR_13421	MAR	landrace	31.792	-7.093	284S	256P	128R	Group1	0.56	87.94	49.08	0.63	90.54	54.45
HOR_13422	MAR	landrace	31.792	-7.093	284S	256A	128G	Group2	0.58	82.83	48.36	0.64	87.93	53.95
HOR_13423	MAR	landrace	31.792	-7.093	284S	256P	128R	Group1	0.53	110.58	58.49	0.58	102.54	57.40
HOR_13424	MAR	landrace	31.792	-7.093	284S	256A	128G	Group2	0.53	93.78	49.81	0.62	96.02	56.68
HOR_13425	MAR	landrace	31.792	-7.093	284S	256A	CT	Het	0.59	79.17	46.91	0.63	82.95	50.95
HOR_13428	MAR	landrace	31.792	-7.093	284S	256A	128G	Group2	0.56	93.78	52.70	0.65	93.56	59.12
HOR_13430	MAR	landrace	31.792	-7.093	284S	256A	128G	Group2	0.52	98.16	51.25	0.61	97.63	58.34
HOR_13431	MAR	landrace	31.792	-7.093	284S	256P	128R	Group1	0.59	93.78	55.59	0.60	94.90	55.29
HOR_13436	MAR	landrace	31.792	-7.093	284S	256A	128G	Group2	0.54	89.40	48.36	0.59	86.36	49.51
HOR_13439	MAR	landrace	31.792	-7.093	284T	256A	128G	Group3	0.57	87.94	49.81	0.57	91.87	50.01
HOR_13441	MAR	landrace	31.792	-7.093	284S	256A	128G	Group2	0.52	104.74	54.87	0.64	91.38	54.51
HOR_13442	MAR	landrace	31.792	-7.093	284S	256A	128G	Group2	0.54	106.20	57.04	0.55	107.60	56.56
HOR_13443	MAR	landrace	31.792	-7.093	284S	256A	128G	Group2	0.56	105.47	59.21	0.60	97.84	57.06
HOR_13445	MAR	landrace	31.792	-7.093	284S	256P	128R	Group1	0.51	101.08	51.25	0.57	92.82	49.40
HOR_13466	ITA	landrace	41.872	12.567	284T	256A	128G	Group3	0.60	104.01	62.10	0.69	102.36	67.40
HOR_13603	MEX	cultivar	23.635	-102.553	284S	256A	128G	Group2	0.65	90.13	58.49	0.63	99.54	61.23
HOR_13731	IND	landrace	20.594	78.963	284S	256P	128R	Group1	0.74	104.01	77.29	0.77	106.61	79.56
HOR_13807	RUS	landrace	55.762	37.617	284T	256A	128G	Group3	0.73	107.66	78.74	0.76	97.09	71.95

Accessions	Country	Genebank_status	Latitute[a]	Longtitute[a]	S284T	A256P	G128R	Hap_group	SS_GH	PSN_GH	SN_GH	SS_BLUES	PSN_BLUEs	SN_BLUEs
HOR_13869	MAR	landrace	31.792	-7.093	284S	256A	128G	Group2	0.53	105.47	56.32	0.62	95.10	56.34
HOR_13870	IRN	landrace	32.428	53.688	284S	256A	128G	Group2	0.69	68.22	46.91	0.73	79.08	55.95
HOR_13888	ESP	landrace	40.464	-3.749	284S	256A	128G	Group2	0.52	97.43	50.53	0.58	100.69	55.56
HOR_13931	ESP	landrace	40.464	-3.749	284S	256A	128G	Group2	0.56	102.54	57.04	0.61	103.86	61.06
HOR_13940	ESP	landrace	40.464	-3.749	284S	256P	128R	Group1	0.54	102.54	55.59	0.56	103.55	55.62
HOR_13945	ESP	landrace	40.464	-3.749	284S	256A	128G	Group2	0.54	114.96	62.10	0.63	104.52	61.40
HOR_13962	IND	landrace	20.594	78.963	284T	256A	128G	Group3	0.54	69.68	37.51	0.66	85.61	55.29
HOR_13983	IRN	landrace	32.428	53.688	284T	256A	128G	Group3	0.58	92.32	53.42	0.59	88.91	51.18
HOR_14096	ISR	breeding_material	31.046	34.852	284S	256A	128G	Group2	0.52	96.70	49.81	0.56	93.65	51.45
HOR_14156	ESP	landrace	40.464	-3.749	284S	256A	128G	Group2	0.53	125.18	66.44	0.59	110.13	62.90
HOR_14164	-	breeding_material	-	-	284S	256A	128G	Group2	0.51	111.31	57.04	0.64	101.14	62.51
HOR_14196	-	landrace	-	-	284S	256P	128G	Others	0.58	90.13	52.70	0.63	105.69	63.40
HOR_14216	PAK	landrace	30.375	69.345	284T	256A	128G	Group3	0.66	75.52	49.81	0.65	86.09	53.90
HOR_14221	NOR	landrace	60.472	8.469	284T	256A	128G	Group3	0.47	108.39	51.25	0.62	100.28	60.06
HOR_14234	NPL	landrace	28.395	84.124	284S	256A	128G	Group2	0.60	81.37	49.08	0.67	79.73	51.12
HOR_14276	BGR	landrace	42.734	25.486	284T	256A	128G	Group3	0.49	98.16	48.36	0.62	95.45	57.12
HOR_14279	SDN	landrace	12.863	30.218	284S	256P	128R	Group1	0.55	117.88	64.27	0.56	113.46	59.95
HOR_14284	BOL	landrace	-16.290	-63.589	284S	256P	128R	Group1	0.51	117.15	59.93	0.58	103.20	57.79
HOR_14334	AFG	landrace	33.939	67.710	284S	256A	128G	Group2	0.55	130.30	72.23	0.66	104.83	66.68
HOR_14376	-	cultivar	-	-	284S	256A	128G	Group2	0.59	103.28	60.66	0.65	108.24	67.01
HOR_14433	-	cultivar	-	-	284T	256A	128G	Group3	0.93	63.11	58.49	0.87	71.38	59.51
HOR_14443	IRN	landrace	32.428	53.688	284T	256A	128G	Group3	0.64	73.33	46.91	0.57	82.82	44.18
HOR_14450	-	breeding_material	-	-	284S	256A	128G	Group2	0.62	99.62	62.10	0.67	100.10	64.18
HOR_14451	-	cultivar	-	-	284S	256A	128G	Group2	0.71	126.65	89.59	0.75	99.66	71.90
HOR_14463	-	cultivar	-	-	284T	256A	128G	Group3	0.73	120.07	88.14	0.73	106.66	76.56
HOR_14476	ESP	landrace	40.464	-3.749	284S	256P	128R	Group1	0.52	106.20	54.87	0.55	105.21	55.01
HOR_14479	-	breeding_material	-	-	284S	256A	128G	Group2	0.71	114.23	81.63	0.71	103.49	70.40
HOR_14480	-	breeding_material	-	-	284S	256A	128G	Group2	0.51	100.35	51.25	0.56	103.97	56.18
HOR_14504	IRN	landrace	32.428	53.688	284S	256A	128G	Group2	0.65	104.01	67.17	0.63	89.37	54.01
HOR_14516	IRN	landrace	32.428	53.688	284S	256A	128G	Group2	0.54	98.16	53.42	0.65	103.79	65.45
HOR_15214	ETH	landrace	9.145	40.490	284S	256A	128G	Group2	0.64	105.47	67.17	0.65	116.58	72.90
HOR_15239	ETH	landrace	9.145	40.490	284S	256A	128G	Group2	0.72	113.50	81.63	0.68	112.71	74.62
HOR_15644	GRC	landrace	39.074	21.824	284S	256P	128R	Group1	0.50	93.78	46.91	0.54	92.88	47.40
HOR_15675	CAN	landrace	56.130	-106.347	284S	256A	128G	Group2	0.65	99.62	65.00	0.63	102.21	61.95
HOR_15698	FRA	landrace	46.228	2.214	284T	256A	128G	Group3	0.76	117.88	89.59	0.81	106.42	83.56
HOR_15699	GRC	landrace	39.074	21.824	284S	256A	128G	Group2	0.52	95.24	49.08	0.58	88.10	49.68
HOR_15708	ROU	landrace	45.943	24.967	284S	256A	128G	Group2	0.66	106.93	70.78	0.66	107.81	69.23
HOR_15735	IND	landrace	20.594	78.963	284S	256P	128G	Others	0.57	101.81	57.76	0.59	86.32	48.23
HOR_15738	IND	landrace	20.594	78.963	284S	256P	128G	Others	0.67	93.05	62.10	0.70	87.32	58.12
HOR_15794	PAK	landrace	30.375	69.345	284T	256A	128G	Group3	0.58	86.48	50.53	0.61	87.50	51.84
HOR_15933	ESP	landrace	40.464	-3.749	284S	256A	128G	Group2	0.53	109.12	57.76	0.57	108.69	58.84
HOR_16935	IND	landrace	20.594	78.963	284S	256A	128G	Group2	0.62	99.62	61.38	0.64	107.31	66.68
HOR_17209	IND	landrace	20.594	78.963	284S	256P	128R	Group1	0.61	97.43	59.21	0.63	93.32	57.56
HOR_17245	-	landrace	-	-	284S	256A	128G	Group2	0.60	99.62	59.93	0.60	96.98	54.79

Accessions	Country	Genebank_status	Latitute[a]	Longtitute[a]	S284T	A256P	G128R	Hap_group	SS_GH	PSN_GH	SN_GH	SS_BLUES	PSN_BLUEs	SN_BLUEs
HOR_17512	-	landrace	-	-	284T	256A	128G	Group3	0.51	106.93	54.15	0.62	97.68	57.06
HOR_17713	AFG	landrace	33.939	67.710	284T	256A	128G	Group3	0.56	122.26	68.61	0.64	103.53	64.68
HOR_17717	-	landrace	-	-	284S	256P	128R	Group1	0.68	116.42	78.74	0.68	119.68	79.51
HOR_17811	CHN	landrace	39.914	116.364	284S	256P	128G	Others	0.69	110.58	75.85	0.65	114.50	71.90
HOR_17832	AFG	landrace	33.939	67.710	284T	256A	128G	Group3	0.64	93.05	59.21	0.57	95.54	52.15
HOR_17839	CHN	landrace	39.914	116.364	284S	256P	128R	Group1	0.68	100.35	67.89	0.70	104.74	70.23

[a]Latitute and longitute are based on country capital

SS: spikelet survival; PSN: potential spiekelt number; SN: final spikelet number; GH: glasshouse; BLUEs: best linear unbiased estimates from three years of field data.

Three amino acid substitutions (S284T, A256P and G128R) observed between BCC149 and BCC719 are listed.

Table S2. Barley mutants used in this study

Name	Line ID	Gene and location	Background	Mutagen and original author
short spike1	sp1	*HvCMF4*, 4HL	Bonus	Gamma-rays induced mutant in Bonus by U. Lundqvist
short spike20	sp20	*HvCMF4*, 4HL	Foma	N-ethyl-N-nitrosourethane induced mutant in Foma by U. Lundqvist
short spike28	sp28	*HvCMF4*, 4HL	Kristina	Gamma-rays induced mutant in Kristina by U. Lundqvist
BW-NIL *tst2.b*	BW883	*HvCMF4*, 4HL	Bowman ($BC_{5\text{-}6}$)	An X-ray induced mutant in Ackermann's Donaria isolated by U. Lundquvist
BW-NIL *Eam1.d*	BW281	*PPD*-H1, 2HS	Bowman (BC_7)	Natural occurrence in Hordeum vulgare subsp. spontaneum and cultivated winter barleys
BM- NIL *eam10.m*	BW284	*HvLUX*	Bowman (BC_4)	An X-ray induced mutant in a mutant stock isolated by E.A. Favret
BM- NIL *eam5.x*	BW286	*HvPhyC*	Bowman (BC_6)	Natural occurrence in Indian cultivars and isolated from a line derived from Higuerilla by H.E. Vivar
BM- NIL *eam-7.g*	BW288	Unknown, 6HS	Bowman (BC_2)	A spontaneous mutant in Atlas (PI 539108) identified as Atsel (CIho 6250) isolated by C.A. Suneson
BM- NIL *mat-b.7*	BW507	Unknown, 7H	Bowman (BC_6)	An X-ray induced mutant in Bonus (PI 189763) isolated by U. Lundqvist
BM- NIL *eam9.l*	BW291	Unknown, 4HL	Bowman (BC_4)	Natural occurrence in Chinese cultivars from the lower basin of the Yangtze River isolated by S. Yasuda
BM- NIL *mat-c.19*	BW508	*HvCEN*	Bowman (BC_5)	A neutron induced mutant in Bonus (PI 189763) isolated by U. Lundqvist
BM- NIL *mat-f.23*	BW511	Unknown, 1H	Bowman (BC_4)	An X-ray induced mutant in Bonus (PI 189763) isolated by U. Lundqvist
BM- NIL *mat-h.36 nl*	BW513	Unknown, 4HL	Bowman (BC_6)	An ethylene imine induced mutant in Bonus (PI 189763) isolated by U. Lundqvist
BM- NIL *mat-d.14*	BW509	Unknown, 4HL or 6HL	Bowman (BC_5)	An X-ray induced mutant in Bonus (PI 189763) isolated by U. Lundqvist
BM- NIL *mat-e.18*	BW510	Unknown	Bowman (BC_4)	A neutron induced mutant in Bonus (PI 189763) isolated by U. Lundqvist
BM- NIL *mat-g.30*	BW512	Unknown	Bowman (BC_4)	An ethylene imine induced mutant in Bonus (PI 189763) isolated by U. Lundqvist
hvcmf7	M4205	*HvCMF7*, 7HL	Barke	EMS-induced mutant in Barke, Li et al., 2019. The Plant Cell. 31: 1430–1445
hvcmf7_2	6460-1	*HvCMF7*, 7HL	Barke	EMS-induced mutant in Barke, Li et al., 2019. The Plant Cell. 31: 1430–1445
hvcmf3_1	4383-1	*HvCMF3*, 6HL	Barke	EMS-induced mutant in Barke, Li et al., 2021. Front. Plant Sci. 12:732608
hvcmf3_2	13082-1	*HvCMF3*, 6HL	Barke	EMS-induced mutant in Barke, Li et al., 2021. Front. Plant Sci. 12:732608

Table S4. QTLs for spike morphology detected in the 130 DH lines

Traits	QTL	Chr	Peak position[a] (cM)	Support interval[b] (cM)	LOD	PVE(%)	Additive effect[c]	Nearby gene
Spikelet surivival	*qSS-2H.1*	2H	193.45	98.78 - 194.96	3.68	9.17	-0.03	
	qSS-4H.1	4H	126.26	94.34 - 141.41	5.93	15.27	0.04	*HvCMF4*
	qSS-7H.1	7H	49.85	49.01 - 57.27	4.56	11.44	0.03	
Days to heading	*qDTH-4H.1*	4H	149.81	120.19 - 153.62	31.94	55.31	-15.23	*VRN-H2*
	qDTH-5H.1	5H	163.35	151.85 - 180.02	15.36	18.78	9.07	*VRN-H1*
Potential spikelet number	*qPSN-4H.1*	4H	149.81	120.19 - 153.62	34.64	59.31	-5.39	*VRN-H2*
	qPSN-5H.1	5H	170.93	155.2 - 216.64	15.52	17.78	2.97	*VRN-H1*
Final spikelet number	*qSN-2H.1*	2H	190.66	167.85 - 206.39	3.91	5.62	-0.86	
	qSN-4H.1	4H	51.10	14.98 - 53.66	4.37	6.33	0.92	
	qSN-4H.2	4H	149.81	66.03 - 153.62	16.24	29.53	-1.90	*VRN-H2*
	qSN-5H.1	5H	170.93	159.54 - 176.23	6.39	9.75	1.11	*VRN-H1*
Degenerated spikelet number	*qSN-2H.1*	2H	183.68	N.S.	6.00	13.70	1.92	
	qSN-3H.1	3H	43.50	N.S.	3.36	4.44	-1.10	
	qSN-4H.1	4H	126.26	94.34 - 153.62	5.48	6.39	-1.31	*HvCMF4*
	qSN-4H.2	4H	149.81		17.44	27.06	-2.68	*VRN-H2*
	qSN-5H.1	5H	169.41	159.54 - 180.02	14.34	22.84	2.47	*VRN-H1*
	qSN-7H.1	7H	50.98	N.S.	6.61	7.89	-1.49	

[a]Peak position was determined based on the ICIM-ADD method;

[b]LOD supported interval determined based on a single-marker analysis;

[c]Positive and negative additive values represent alleles from BCC719 increase or decrease the phenotypic values relative to the BCC149 alleles;

N.S., not significant. Note that QTLs were detected with ICIM-ADD, but were not detected with a signal-marker analysis

Table S5. Phenotypes of key recombinants from $F_{2\text{-}3}$ generation of BW × *tst2.b* mapping population

Plant ID	Spike length (cm)	Spikelet number	F_2										F_3
			MAP4H_39 605090607	MAP4H_48 605680388	MAP4H_46 605770453	MAP4H_54 605826323	MAP4H_36.1 606289468	MAP4H_45 606576904	MAP4H_49 606756865	MAP4H_47 606800935	MAP4H_51 607105868	MAP4H_42 607879736	
BW	8.4	24	A	A	A	A	A	A	A	A	A	A	-
tst2.b	4.6	13	B	B	B	B	B	B	B	B	B	B	-
p.108	8	22	A	A	A	A	A	H	H	H	H	H	Wild type
p.10	8.5	24	A	A	A	A	A	H	H	H	H	H	Wild type
p.12	4.7	12	B	B	B	B	B	H	H	H	H	H	Mutant type
p.14	4.3	12	H	H	H	H	B	B	B	B	B	B	Mutant type
p.48	8.1	24	B	B	B	B	H	H	H	H	H	H	Segregating
p.66	8.2	23	H	H	H	H	H	A	A	A	A	A	Segregating
p.77	4.3	12	H	H	H	B	B	B	B	B	B	B	Mutant type
p.71	4.3	12	A	A	A	B	B	B	B	B	B	B	Mutant type
p.18	7.5	22	B	H	H	H	H	H	H	H	H	H	Segregating
p.25	7.5	22	H	H	H	H	H	H	H	H	A	A	Segregating
p.27	7.8	23	H	H	H	H	A	A	A	A	A	A	Wild type
p.43	4.5	12	H	B	B	B	B	B	B	B	B	B	Mutant type
p.56	8.6	25	H	H	H	H	H	H	H	H	H	A	Segregating
p.74	8.3	25	H	A	A	A	A	A	A	A	A	A	Wild type

Marker MAP4H_36.1 (highlight in red) was designed based on the 4-bp deletion found in HvCMF4, and is co-segregating with the mutant phenotype. Numbers below each marker represent physical position on chr4H based on Morex reference V2;

H: Heterozygote; A: Bowman genotype; B: tst2.b genotype;

Numbers below each marker represent physical position from barley chromosome 4H based on Morex v2.

Table S6. Phenotypes of the short spike mutants and allelism test

Name	N	PSN	SL (cm)	SN	GN	Description
F1: *tst2.b×sp20*	4	-	4.18 ± 0.06	11.25 ± 0.63	1 ± 0.41	F1 for allelism test
short spike20	12	43.83 ± 0.48, *	5.23 ± 0.28, ***	13.92 ± 0.43, ***	1.92 ± 0.51, ***	TAT - TAA, stop gain in HvCMF4
tst2.b	13	36.13 ± 0.35	4.56 ± 0.23	12.05 ± 0.46	3 ± 0.87	4-bp deletion in HvCMF4
Bowman	13	36.17 ± 0.29	8.57 ± 0.15	24.23 ± 0.24	20.26 ± 0.27	WT background of tst2.b
short spike1	12	44.33 ± 0.33, ns	5.77 ± 0.36, ***	14.61 ± 0.67, ***	1.92 ± 0.36, ***	1-bp deletion, frame shift in HvCMF4
short spike28	24	44.5 ± 0.33, *	5.2 ± 0.2, ***	14.49 ± 0.39, ***	2.06 ± 0.23, ***	Gene deletion in HvCMF4
Bonus	14	44.88 ± 0.4	12.02 ± 0.21	33.41 ± 0.36	20.21 ± 0.64	WT of short spike1
Foma	13	45.38 ± 0.26	10.3 ± 0.21	32.59 ± 0.37	23.9 ± 0.68	WT of short spike20
Kristina	13	43.14 ± 0.34	10.39 ± 0.2	32.36 ± 0.45	17.95 ± 1.15	WT of short spike28

N: sample number; PSN: potential spikelet number; SL: spike length; SN: final spikelet number; GN: grain number;

Significance levels are determined from two-tailed Student's *t*-test. *$P < 0.05$; **$P < 0.01$; ***$P < 0.001$, ****$P < 0.0001$ and ns, not significant. Each paire of mutant and the corresponding wild-type (WT) is used for comparison.

Table S7. Raw data of chlorophyll, phytohormone and carbohydrate concentrations

Sample information				Chlorophyll (mg/g F.W.)		Phytohormone (pg/mg F.W.)									Carbohydrate (µmol/g F.W.)			
Geno	Stage	Position	Rep	Chl a	Chl b	GA15	GA19	GA44	IAA	IPR	CZR	ABA	PA	DHPA	Starch	Sucrose	Fructose	Glucose
BW	W4.5	Apical	1	0.04	0.07	24.97	48.39	20.58	52.82	4.44	4.65	10.88	145.41	14.07	5.04	3.45	0.24	n.d.
BW	W4.5	Apical	2	0.04	0.07	20.87	44.36	18.15	60.14	1.36	2.85	8.93	121.92	-	6.33	2.62	0.30	n.d.
BW	W4.5	Apical	3	0.04	0.08	27.93	51.39	20.74	161.24	2.62	4.39	9.91	130.93	10.99	4.92	2.02	-	n.d.
BW	W4.5	Apical	4	0.05	0.08	24.13	49.07	21.11	156.91	3.45	4.65	11.27	157.73	17.38	5.65	2.44	0.30	n.d.
BW	W4.5	Apical	5	0.07	0.12	-	-	-	-	-	-	-	-	-	4.59	1.78	0.53	n.d.
BW	W4.5	Central	1	0.06	0.11	6.00	21.69	8.44	48.62	-	-	14.65	210.67	12.76	16.65	10.84	0.81	n.d.
BW	W4.5	Central	2	0.07	0.10	6.68	22.16	9.63	82.23	2.40	17.42	17.77	193.40	11.35	16.37	7.17	0.75	n.d.
BW	W4.5	Central	3	0.07	0.11	6.23	21.66	9.85	72.29	2.18	20.79	21.69	244.13	7.52	13.03	7.08	0.61	n.d.
BW	W4.5	Central	4	0.10	0.15	-	-	-	-	-	-	-	-	-	14.11	8.99	-	n.d.
BW	W4.5	Central	5	0.10	0.16	-	-	-	-	-	-	-	-	-	-	-	0.12	n.d.
BW	W4.5	Basal	1	0.09	0.15	4.67	20.78	5.90	64.41	2.86	25.35	17.19	157.83	27.73	7.34	8.58	0.47	n.d.
BW	W4.5	Basal	2	0.11	0.17	4.79	20.55	6.08	40.07	3.28	34.73	20.66	157.31	24.66	8.57	9.85	0.44	n.d.
BW	W4.5	Basal	3	0.11	0.18	4.46	20.44	5.71	56.54	-	-	18.77	150.61	28.37	8.56	10.78	0.40	n.d.
BW	W4.5	Basal	4	0.12	0.16	3.68	21.02	5.74	58.29	6.26	-	24.69	182.09	26.97	8.18	9.83	0.45	n.d.
BW	W4.5	Basal	5	0.14	0.22	-	-	-	-	-	-	-	-	-	8.70	8.67	0.45	n.d.
BW	W7	Apical	1	0.09	0.15	15.85	37.13	16.62	60.27	1.01	10.19	75.33	257.80	9.28	13.63	6.21	0.28	n.d.
BW	W7	Apical	2	0.10	0.16	9.00	29.64	13.45	53.56	6.23	77.55	45.12	339.56	8.84	15.70	7.26	0.53	n.d.
BW	W7	Apical	3	0.10	0.14	12.34	30.82	14.10	65.90	1.03	10.00	50.01	224.52	11.25	15.57	6.21	0.46	n.d.
BW	W7	Apical	4	0.15	0.25	13.32	32.76	16.24	53.83	6.49	80.19	49.45	318.50	7.94	11.91	5.03	-	n.d.
BW	W7	Apical	5	0.17	0.30	-	-	-	-	-	-	-	-	-	17.87	8.20	0.33	n.d.
BW	W7	Central	1	0.17	0.20	7.68	30.15	17.85	147.97	27.51	369.63	75.73	297.43	26.09	18.15	14.92	0.97	n.d.
BW	W7	Central	2	0.17	0.23	7.87	30.47	17.58	91.04	26.75	373.94	58.56	398.56	28.79	30.97	15.78	0.42	n.d.
BW	W7	Central	3	0.22	0.27	7.10	28.05	16.05	151.02	30.62	425.09	50.09	287.47	16.33	26.82	13.92	0.75	n.d.
BW	W7	Central	4	0.24	0.27	6.45	27.98	17.39	79.96	27.02	388.43	56.53	344.73	19.39	27.40	12.71	0.30	n.d.
BW	W7	Central	5	0.27	0.31	-	-	-	-	-	-	-	-	-	31.58	15.44	0.54	n.d.
BW	W7	Basal	1	0.23	0.25	6.77	28.18	11.15	107.71	22.03	290.16	46.32	251.79	30.39	17.36	15.65	0.71	0.29
BW	W7	Basal	2	0.28	0.29	8.56	34.33	12.99	145.31	9.95	104.26	37.78	359.84	52.18	17.73	15.91	0.44	0.53
BW	W7	Basal	3	0.33	0.34	6.47	28.12	11.22	73.29	31.39	405.92	36.45	218.74	33.16	19.63	16.44	0.70	0.16
BW	W7	Basal	4	0.38	0.47	7.60	29.82	12.14	111.86	22.52	229.02	39.39	253.23	32.09	17.52	15.38	1.23	-
BW	W7	Basal	5	0.39	0.47	-	-	-	-	-	-	-	-	-	22.25	15.44	1.19	-
BW	W5.5	Apical	1	0.08	0.12	10.34	23.37	8.27	58.54	1.18	18.54	215.03	241.00	19.46	15.05	10.42	2.42	0.25
BW	W5.5	Apical	2	0.09	0.16	10.51	24.90	9.52	29.24	1.14	35.66	154.66	179.23	6.32	9.06	8.12	2.05	0.60
BW	W5.5	Apical	3	0.11	0.18	7.86	21.72	8.27	42.43	3.07	63.73	199.86	215.12	8.33	15.18	9.18	1.86	0.59
BW	W5.5	Apical	4	0.11	0.18	9.63	25.59	9.57	38.65	1.93	39.47	235.98	277.54	12.15	12.28	10.30	1.76	0.38
BW	W5.5	Apical	5	0.11	0.17	-	-	-	-	-	-	-	-	-	11.66	10.53	2.65	0.80
BW	W5.5	Central	1	0.14	0.16	7.35	27.16	13.13	111.33	12.62	193.55	52.29	135.60	50.79	25.14	14.75	2.27	1.53
BW	W5.5	Central	2	0.14	0.18	6.12	25.94	12.11	77.24	13.81	186.86	39.37	88.64	30.53	26.63	14.17	2.23	1.67
BW	W5.5	Central	3	0.18	0.21	6.83	25.53	11.84	-	25.68	372.88	59.39	133.54	41.09	34.31	18.34	2.83	1.94
BW	W5.5	Central	4	0.18	0.23	7.02	28.01	12.68	116.66	15.26	210.77	52.99	114.02	36.47	28.15	14.50	1.74	1.06
BW	W5.5	Central	5	0.22	0.27	-	-	-	-	-	-	-	-	-	30.79	-	-	-
BW	W5.5	Basal	1	0.18	0.20	7.15	27.50	11.67	115.52	9.29	102.74	45.73	131.33	76.04	15.24	12.32	2.30	1.61
BW	W5.5	Basal	2	0.21	0.25	7.34	27.10	11.00	103.06	12.33	163.53	33.15	91.57	45.75	16.75	15.38	2.73	1.83
BW	W5.5	Basal	3	0.22	0.27	6.15	27.31	11.13	67.61	22.88	320.31	41.98	110.56	45.20	16.42	13.63	3.18	1.98

Sample information				Chlorophyll (mg/g F.W.)		Phytohormone (pg/mg F.W.)									Carbohydrate (µmol/g F.W.)			
**Geno**	**Stage**	**Position**	**Rep**	**Chl a**	**Chl b**	**GA15**	**GA19**	**GA44**	**IAA**	**IPR**	**CZR**	**ABA**	**PA**	**DHPA**	**Starch**	**Sucrose**	**Fructose**	**Glucose**
BW	W5.5	Basal	4	0.22	0.29	7.57	27.34	11.41	72.94	11.88	166.66	38.39	110.59	50.58	13.79	14.18	2.09	1.22
BW	W5.5	Basal	5	0.31	0.38	-	-	-	-	-	-	-	-	-	16.95	15.50	2.76	1.84
tst2.b	W4.5	Apical	1	0.02	0.04	15.39	27.50	10.76	7.30	0.00	2.28	5.63	26.12	0.00	6.09	4.12	0.46	n.d.
tst2.b	W4.5	Apical	2	0.03	0.06	16.19	31.26	12.68	37.44	0.66	6.55	5.70	58.05	0.00	4.36	6.66	-	n.d.
tst2.b	W4.5	Apical	3	0.03	0.05	16.77	27.91	11.90	48.62	1.12	6.15	4.63	49.11	0.00	3.42	2.21	0.17	n.d.
tst2.b	W4.5	Apical	4	0.03	0.06	13.47	25.51	9.94	42.53	0.54	7.37	5.97	44.60	0.00	5.12	2.92	0.16	n.d.
tst2.b	W4.5	Apical	5	0.04	0.08	-	-	-	-	-	-	-	-	-	5.51	5.58	0.56	n.d.
tst2.b	W4.5	Central	1	0.04	0.07	4.75	19.96	7.77	34.53	1.17	20.94	13.75	188.05	7.36	40.28	16.10	0.47	-
tst2.b	W4.5	Central	2	0.06	0.09	5.31	20.21	7.26	36.66	1.11	23.04	12.34	201.34	5.22	27.48	10.58	0.20	0.12
tst2.b	W4.5	Central	3	0.06	0.11	4.85	19.19	7.78	48.44	1.08	19.02	8.16	137.35	7.20	19.71	8.64	-	0.46
tst2.b	W4.5	Central	4	0.06	0.11	4.07	20.08	6.83	37.28	1.43	18.10	10.88	138.80	6.26	-	8.05	0.09	-
tst2.b	W4.5	Central	5	0.06	0.11	-	-	-	-	-	-	-	-	-	30.59	10.75	-	0.47
tst2.b	W4.5	Basal	1	0.07	0.12	6.99	26.21	8.41	63.64	1.55	23.90	17.05	167.07	15.11	24.50	15.14	0.26	-
tst2.b	W4.5	Basal	2	0.07	0.11	7.24	28.47	8.32	82.39	1.64	26.21	17.37	187.65	24.24	29.42	18.52	1.96	1.85
tst2.b	W4.5	Basal	3	0.08	0.12	7.92	30.09	9.83	89.75	2.06	28.06	18.49	150.48	15.69	10.72	9.94	-	-
tst2.b	W4.5	Basal	4	0.08	0.13	7.61	28.78	8.41	35.47	1.36	27.25	18.35	134.37	18.01	10.09	12.20	0.54	0.17
tst2.b	W4.5	Basal	5	0.10	0.16	-	-	-	-	-	-	-	-	-	21.61	13.45	0.35	-
tst2.b	W7	Apical	1	0.03	0.06	39.27	63.32	0.00	157.33	1.34	94.17	88.28	225.68	0.00	6.70	5.55	-	-
tst2.b	W7	Apical	2	0.04	0.07	105.60	135.45	0.00	260.51	2.92	134.44	189.61	469.97	0.00	3.51	5.54	0.88	0.33
tst2.b	W7	Apical	3	0.04	0.09	43.46	75.11	0.00	365.41	1.55	95.16	226.98	329.09	0.00	2.52	3.02	1.48	0.20
tst2.b	W7	Apical	4	0.05	0.11	34.38	60.23	0.00	52.63	1.33	75.00	103.42	188.78	0.00	5.35	4.71	0.80	-
tst2.b	W7	Apical	5	0.07	0.13	-	-	-	-	-	-	-	-	-	-	4.62	-	-
tst2.b	W7	Central	1	0.07	0.10	8.30	31.34	15.52	66.99	3.31	70.87	43.96	350.17	20.22	34.64	15.89	0.91	0.38
tst2.b	W7	Central	2	0.07	0.11	10.64	34.84	15.87	58.60	2.14	74.01	56.88	364.02	19.56	37.55	17.56	-	-
tst2.b	W7	Central	3	0.08	0.11	7.55	30.82	15.61	77.60	4.07	96.45	46.42	312.06	18.07	23.14	17.70	0.88	0.13
tst2.b	W7	Central	4	0.08	0.13	8.18	30.92	15.17	67.44	2.93	87.50	50.68	372.05	19.67	42.55	19.57	0.57	0.18
tst2.b	W7	Central	5	0.09	0.13	-	-	-	-	-	-	-	-	-	-	25.25	0.95	0.40
tst2.b	W7	Basal	1	0.11	0.15	8.02	33.33	14.09	79.66	5.62	107.45	32.56	227.24	24.92	32.28	24.18	1.14	0.76
tst2.b	W7	Basal	2	0.13	0.18	8.78	35.12	15.52	100.16	4.28	102.68	38.29	293.58	31.64	22.27	23.69	0.98	0.41
tst2.b	W7	Basal	3	0.15	0.23	5.93	34.14	14.44	117.58	4.22	58.03	42.26	302.77	46.17	43.38	26.94	2.14	1.44
tst2.b	W7	Basal	4	0.18	0.25	8.27	34.50	15.52	85.23	3.53	31.19	25.58	215.02	20.46	29.38	25.20	1.61	1.12
tst2.b	W7	Basal	5	0.24	0.32	-	-	-	-	-	-	-	-	-	24.78	-	1.18	0.65
tst2.b	W5.5	Apical	1	0.03	0.06	28.21	28.25	0.00	150.81	0.66	9.30	157.03	158.82	0.00	2.42	2.23	1.22	0.49
tst2.b	W5.5	Apical	2	0.04	0.08	13.20	25.93	0.00	53.93	0.58	8.56	103.65	134.31	0.00	1.25	2.62	-	-
tst2.b	W5.5	Apical	3	0.04	0.06	15.28	28.18	0.00	48.82	0.65	2.39	51.32	50.71	0.00	1.54	2.21	2.46	0.63
tst2.b	W5.5	Apical	4	0.06	0.11	14.37	27.27	0.00	144.67	0.31	9.30	146.86	197.50	0.00	1.61	2.97	-	0.55
tst2.b	W5.5	Apical	5	0.07	0.11	-	-	-	-	-	-	-	-	-	2.45	2.54	2.34	0.78
tst2.b	W5.5	Central	1	0.07	0.13	7.21	30.52	13.69	78.30	3.56	42.14	125.77	380.58	98.01	38.92	20.28	1.65	0.57
tst2.b	W5.5	Central	2	0.07	0.13	8.53	34.78	15.10	67.63	4.36	66.76	119.46	348.02	89.46	47.85	19.15	-	-
tst2.b	W5.5	Central	3	0.07	0.12	5.95	27.97	13.00	48.05	3.08	59.68	108.73	307.24	57.92	47.84	-	0.83	0.50
tst2.b	W5.5	Central	4	0.10	0.17	6.74	33.57	15.36	74.61	3.21	39.97	109.46	312.25	88.03	59.27	23.56	1.06	0.27
tst2.b	W5.5	Central	5	0.11	0.19	-	-	-	-	-	-	-	-	-	49.03	22.58	1.86	0.59
tst2.b	W5.5	Basal	1	0.10	0.14	7.03	33.03	18.50	195.29	2.94	27.95	32.43	193.71	98.57	37.79	22.46	1.47	0.64
tst2.b	W5.5	Basal	2	0.10	0.15	6.83	38.11	18.94	105.19	4.46	70.13	53.07	163.76	131.14	39.20	23.45	1.08	0.64

Sample information				Chlorophyll (mg/g F.W.)		Phytohormone (pg/mg F.W.)									Carbohydrate (µmol/g F.W.)			
**Geno**	**Stage**	**Position**	**Rep**	**Chl a**	**Chl b**	**GA15**	**GA19**	**GA44**	**IAA**	**IPR**	**CZR**	**ABA**	**PA**	**DHPA**	**Starch**	**Sucrose**	**Fructose**	**Glucose**
tst2.b	W5.5	Basal	3	0.11	0.17	6.15	33.14	18.49	130.22	2.98	19.84	31.25	150.86	82.52	31.41	20.10	1.36	0.51
tst2.b	W5.5	Basal	4	0.16	0.21	7.77	41.52	21.50	203.09	3.07	20.06	33.04	149.31	115.46	36.76	25.68	0.95	0.79
tst2.b	W5.5	Basal	5	0.21	0.30	-	-	-	-	-	-	-	-	-	40.69	22.37	1.06	0.48

W4.5: awn primordium stage; W5.5: white anther stage; W7: green anther stage;
GA15, GA19 and GA44: gibberellins; IAA: indole-3-acetic acid for auxin; IPR and CZR: N(6)-isopentenyladenosine and *cis*-zeatine riboside, respectively, for cytokinins; ABA: abscisic acid; PA and DHPA: phaseic acid and dihydrophaseic acid, respectively, catabolic products of ABA;
n.d.: undetectable.

Table S15. Primers used in this study

Purpose	Name	Forward (5' – 3')	Reverse (5' – 3')	Note
Gene mapping and sequence	MAP4H_39	TCCCGGGTCTTTTTATGATG	TCTCGATAGTCGTGCAAACG	Recombinant screening, after XhoI digested: 520/280+240 bp
	MAP4H_48	TCTCGTGCTTCGCCTGAAC	CCTGAAGCTTGTTGCAGAA	Recombinant screening, after Eco130I digested: 367/174+193 bp
	MAP4H_46	AGTACTTGGACAATCTGAGCA	CATTCCTGCAGAGAAGACACA	Recombinant screening, after BseJI digested: 327/107+220 bp
	Map4H_54	AAAGGAACCAATGGTCAAATG	ATTTTTGCGCCAAATCAATTA	Left franking marker, after BsrI digested: 388/249+139 bp
	MAP4H_36.1	TACAGGAGGGGATCTCAAGC	TTACGAGTGCTGCATGGAAG	Co-segregating marker, after HaeIII digested: 798/350+448 bp
	Map4H_45	GATTTAAGGAATGGAACGACT	GGTTTGCTAAAGCATAGATGA	Right franking marker, after Alw21I digested: 388/108+280 bp
	MAP4H_49	AAACCCCAGTGACCCGTTA	GAATGGACCAAGTTTGTGAT	Recombinant screening, after Eco130I digested: 412/219+193 bp
	MAP4H_47	GAGGGATCATCAAGGTCCTG	TCCTTGTCGTGGAAGTACCAG	Recombinant screening, after Bsh1285I digested: 385/170+215 bp
	MAP4H_51	TTGCATCCAGAATGTAAGTTT	GACAACGTGATGCTTAGAGAG	Recombinant screening, after HhaI digested: 500/183+317 bp
	MAP4H_42	TTATGCGTCATGAACCCTGT	GTTATAGCCCACGCAAATGG	Recombinant screening, after HinfI digested: 449/130 + 319 bp
	CMF4_Seq	GACCCACCCTCTAACTCCAT	TGGCATCAAGTGAGAACAGA	HvCMF4 sequencing primers
qRT-PCR	qRT_HvCMF4	CCCAGCCCATTTCATACCTA	AGGCCGTCATCACAGAAATC	Morex V2 id: HORVU.MOREX.r2.4HG0343050
	qRT-HvTOC1	GAGCATAGCATGGCACTTCA	TGTCTTTCCTCGGAAATTGG	Morex V2 id: HORVU.MOREX.r2.6HG0493270
	qRT-HvLHCB2.3	CACACACCATCCTCCAGTTG	CAGGTACTTGGGACGGTCAG	Morex V2 id: HORVU.MOREX.r2.5HG0414440
	qRT-HvPIF3.1	GTCGGGGCGGTCTAACTACT	AGCGCCAAGGACTCTGACT	Morex V2 id: HORVU.MOREX.r2.5HG0422460
	qRT-HvCCA1	CAGGTCACCAGCACTCAGAA	GTTCAATTCCCCCAGAGACA	Morex V2 id: HORVU.MOREX.r2.7HG0579870
	qRT-HvActin	AAGTACAGTGTCTGGATTGGAGGG	TCGCAACTTAGAAGCACTTCCG	Morex V2 id: HORVU.MOREX.r2.5HG0378970
CIRSPR/Cas9 and subcellular localization	CMF4_Guide1	TGGCGCAAACCTTCCACGGACACG	TTTGCGTGTCCGTGGAAGGTTTGC	gRNA#1 for HvCMF4
	CMF4_Guide2	TGGCATCGCTGTAGCTGCAGAG	TTTGCTCTGCAGCTACAGCGAT	gRNA#2 for HvCMF4
	CMF4_Guide3	TGGCGAGAGAGTAGAGTCAAAGCA	TTTGTGCTTTGACTCTACTCTCTC	gRNA#3 for HvCMF4
	CMF4L1_Guide1	TGGCGTGGATACAGGGCAATATCC	TTTGGGATATTGCCCTGTATCCAC	gRNA#1 for HvCMF4L1
	CMF4_GFP	CACCATGATGAAGATGTTCTTCGA	GAAGGTGAAGGGGTTGTGTA	CMF4-YFP construct
mRNA in situ hybridization	*HvCMF4*	CCCAGCCCATTTCATACCTA	CAGTGGAGGAGGATGAAACAT	Morex V2 id: HORVU.MOREX.r2.4HG0343050
	HvLHCB2.3	GGACATCTCCACCCAAACAC	ACTTGATGCCGTTCTTGGAC	Morex V2 id: HORVU.MOREX.r2.5HG0414440
	HvPIF3.1	TCGACAAGGACATCTTCACG	GCGCCGAAGCAGTAGTTAGA	Morex V2 id: HORVU.MOREX.r2.5HG0422460
	HvCCA1	ATGCTTGCTGTGTCTTGTCG	CAAGCATGGCTTCTGTGGTA	Morex V2 id: HORVU.MOREX.r2.7HG0579870
	PPD-H1	GATGAAGCAGGGCTCTAACG	GCTCCCGTTATTGGTGTTGT	Morex V2 id: HORVU.MOREX.r2.2HG0088300
	HvWOX4	GGCCTCCTACCTGGAGAAAG	GAAGAGTCGAGGGTGCTGAG	Morex V2 id: HORVU.MOREX.r2.2HG0170340
	HvVND1	GATGGGGCTCAAACTACTCC	TCCACCTCTCCATCTCATCC	Morex V2 id: HORVU.MOREX.r2.4HG0342310
	HvAPL-like	AGTCCATCCTGGAGAAAGCA	CCTCTGCTCCCAGTTGAGTC	Morex V2 id: HORVU.MOREX.r2.6HG0473270
	HvSCPL48	ACGACGCCAGGATGTTCTAC	TGGTGGGATGAATTTGTTGA	Morex V2 id: HORVU.MOREX.r2.6HG0452780
	HvGLK1	CTAGTCCGGCCATATTCAGC	GAAGAATCGTCCGTCGTCAC	Morex V2 id: HORVU.MOREX.r2.3HG0207800
	HvHISTONE4	AACTCGTTACAAGCACCAGCA	ACATCAATTCCCCAATCCCCT	Morex V2 id: HORVU.MOREX.r2.1HG0042800

Legends for Table S3, S8 to S14

Table S3. Genotype and phenotype of the 130 DH lines.

Table S8. DYGs identified from rachis (R1) and spikelet (R2) comparison across six developmental stages.

Table S9. DYGs identified from spike sections of BW and tst2.b across three developmental stages.

Table S10. DYGs identified from BW and tst2.b anthers across two developmental stages.

Table S11. Summary of enriched GO terms from the transcriptomic studies.

Table S12. Summary of upstream TFs enriched from each of the ten spike sections clusters predicted.

Table S13. Full gene lists for cell cycle, programmed cell death, floral meristem development, stress associaetd TFs, photosynthesis and energy metabolism.

Table S14. Curated gene lists for cell cycle, programmed cell death, chloroplast development, vascular patterning and photomorphogenesis.
